# Profile Screening of Differentially Expressed lncRNAs of Circulating Leukocytes in Type 2 Diabetes Patients and Differences From Type 1 Diabetes

**DOI:** 10.3389/fendo.2021.690555

**Published:** 2022-01-10

**Authors:** Jianyi Lv, Yihan Liu, Jia Cui, Hongjuan Fang, Ying Wu, Xiao Zhu, Meng Guo, Changlong Li, Jingtao Dou, Zhenwen Chen, Xiaoyan Du

**Affiliations:** ^1^ School of Basic Medical Sciences, Capital Medical University, Beijing, China; ^2^ Department of Endocrinology, Chinese PLA General Hospital, Beijing, China; ^3^ Department of Endocrinology, Beijing TianTan Hospital, Capital Medical University, Beijing, China

**Keywords:** circulating leukocyte, distinguishing diagnosis, lncRNA, RNA sequencing, type 2 diabetes, type 1 diabetes

## Abstract

Long noncoding RNAs (lncRNAs) have been reported to have multiple functions and can be used as markers of various diseases, including diabetes. This study was conducted to determine the lncRNA profile in leukocytes from patients with type 2 diabetes (T2D). Differential expression of lncRNAs in T2D and type 1 diabetes (T1D) was also examined. RNA sequencing was performed in a critically grouped sample of leukocytes from T2D patients and healthy persons. A total of 845 significantly differentially expressed lncRNAs were identified, with 260 downregulated and 585 upregulated lncRNAs in T2D. The analysis of functions of DE-lncRNA and constructed co-expression networks (CNC) showed that 21 lncRNAs and 117 mRNAs harbored more than 10 related genes in CNC. Fourteen of 21 lncRNAs were confirmed to be significantly differentially expressed was detected by qPCR between the T2D and control validation cohorts. We also identified a panel of 4 lncRNAs showing significant differences in expression between T1D and T2D. Collectively, hundreds of novel DE-lncRNAs we identified in leukocytes from T2D patients will aid in epigenetic mechanism studies. Fourteen confirmed DE-lncRNAs can be regarded as diagnostic markers or regulators of T2D, including 4 lncRNAs that chould distinguish T1D and T2D in clinical practice to avoid misdiagnosis.

## Introduction

Diabetes mellitus (DM) is one of the most widespread chronic diseases, creating a burden for both patients and their families. DM is always divided into four main types, among which patients with type 2 diabetes (T2D) account for over 90% and type 1 diabetes (T1D) with approximately 10% of all cases (1). Genetic and environmental factors are important causes of T2D. Although the characteristics of type 1 and 2 diabetes differ, quickly or easily distinguishing these types is difficult in clinical practice. Additionally, as the age of T2D patients decreases, it becomes difficult to distinguish type 1 and 2 diabetes in patients 20–30 years old (1). Thus, T1D patients may be misdiagnosed as having T2D, resulting in incorrect treatment. Diagnosing different types of diabetes mellitus still remains a challenge. Therefore, new markers or therapy targets are urgently needed for T2D. Also, simple, convenient, and efficient molecular technological methods that aid in the diagnoses of different types of diabetes are needed as well.

Long noncoding RNAs (lncRNAs) are a class of RNAs with transcripts longer than 200 nucleotides and limited protein coding potential (2). Studies have demonstrated that lncRNAs are involved in a wide range of biological processes (3). A growing mass of literature has revealed that lncRNAs are useful markers of disease status and in diagnosis (4). Few previous studies have investigated lncRNAs in diabetes (5). Most reports of lncRNAs in diabetes have mainly focused on the lncRNA profile in pancreatic beta cells and regulation of glucose homeostasis (6), diabetes with vascular complications (7), nephropathy (8), obesity (9), and retinopathy (10). Researchers have established a connection between lincRNA transcriptome of human monocyte-derived macrophages and cardiometabolic disorders (11). However, studies aimed at identifying lncRNAs involved in T2D in circulating leukocytes are rare. Systemic low-grade inflammation is a hallmark of T2D and contributes to the pathogenesis of several associated complications (12). Activation of monocytes and macrophages plays an important role in inflammatory processes needed for the protection against invading pathogens or toxins (13). We predicted that lncRNA in peripheral blood cells may play a regulatory role in the pathogenesis of T2D. Wang et al. performed microarray analysis to screen out differential expressed mRNAs and lncRNAs between T2D patients and healthy control (14). Marpadga et al. revealed downregulation of anti-inflammatory cytokines and antiproliferative genes, along with several lncRNAs, may promote chronic inflammation in T2D with RNA sequencing (15). Whether lncRNAs are involved blood cells or if they can be used to distinguish type 1 and 2 diabetes has not been explored. Therefore, we identified differentially expressed lncRNAs in leukocytes, the most nucleated cells in circulation, and explored novel lncRNAs as diagnostic markers and potential regulators for T2D. We also evaluated lncRNAs that can discriminate type 2 and type 1 diabetes by analyzing the present data and our previous data (GEO accession number GSE130279).

## Materials and Methods

### Subjects

Human blood samples collected from the Department of Endocrinology, Chinese PLA General Hospital, were grouped into healthy control (CTR, n = 5), and T2D (n = 11, 6 with family history and 5 without family history) as discovery cohort by clinical examination. The expanded validation cohort were grouped by healthy control (CTR, n = 36), T2D (n = 56), and T1D (n = 11). The information of these T2D participants in discovery cohort and validation cohort are showed in **Table S1**. All consenting adult subjects (18–65 years old) with no past medical history were consecutively enrolled between March 2017 and January 2018. Blood samples were subsequently collected in the morning after an overnight fast of 10 to 12 h. The study was approved by the Ethics Committee of the Chinese PLA General Hospital (Permitted No. S2016-147-03) and all patients gave informed consent before participation in the study.

### Detailed Information of Type 2 Diabetes Diagnosed Criteria and Excluded Requirements

The individuals in this study either suffered from T2D, T1D or were healthy controls, as part of discovery or validation cohorts. The subjects in the T2D or T1D group were involved based on criteria including: 1) diagnosed with T2D or T1D according the World Health Organization (WHO) screening criteria; 2) aged 18–65 years without a gender limit; 3) with normal triglyceride serum level; 4) no complications occurred; and 5) with the approval of the participants themselves or their guardian. Patients with family history were defined as such by the existence of a parent and grandparent also diagnosed as having T2D. The subjects in the healthy control group were included based on the following criteria: 1) healthy with a negative diagnosis of T2D and T1D according the World Health Organization (WHO) screening diabetes criteria and confirmed with normal blood biochemical indexes, free from all endocrine disease and infectious disease by consulting; 2) aged 18–65 years without a gender limit; 3) with the approval of the participants themselves or their guardian. Exclusion criteria were: 1) severe disease or tumors in the heart, brain, liver, kidney currently or previously; 2) severe gastrointestinal disease; 3) presenting with other conditions such as severe infection or active tuberculosis and applying multiple antibiotics; 4) pregnant or lactating women; 5) with a history of or alcohol and/or drug abuse currently; 6) with a history of mental illness or with family history; and 7) stressful life incidents having occurred within one year. The information of healthy control was gained by consulting.

### Total RNA Extraction and Purification From Leukocytes

Total RNA was extracted from leukocytes isolated from peripheral blood. Briefly, approximately 2.5 ml of blood from each subject was static settlement for less than 4 h, followed by centrifugation for 10 min at 3,000×*g*. The cell pellets were then incubated with 1 ml red blood cell lysate (Solarbio Technology Co., Ltd.) and the resultant lysate was centrifuged for 3 min at 3,000×*g*. After that, the supernatant was removed and the above steps were repeated three times. Total RNA was extracted using TRIzol, as per the manufacturer’s instructions (Invitrogen). Ribosomal RNA was removed using the Ribo Zero Magnetic Gold kit (MRZG126, Illumina). Quality and integrity of the isolated RNA was verified by NanoDrop (Thermo scientific) and Bioanalyzer (Agilent). OD260/280 ratio ranged between 1.9 and 2.1 and RIN >7.0.

### RNA-Sequencing and Analysis

RNA sequencing was performed by Annoroad Gene Tech. Co., Ltd. Sequence libraries were prepared using the NEB Next Ultra Directional RNA LibraryPrep Kit for Illumina (NEB, Ispawich, USA) selection in order to include all the lncRNA transcripts that were not polyadenylated. Libraries were sequenced on the Illumina HiSeq X Ten with 150 bp paired-end reads. The paired-end reads from the samples were mapped to the hg19 reference genome by HISAT (v2.0.5). *Ab initio* transcript reconstruction was performed using String Tie, version 1.3.2d, with the reference genome download from the ENSEMBL. Novel transcripts were filtered for having at least 2 exons. Read counts were then calculated per gene from the alignment bam files using HTSeq (v0.6.0) and FPKM (Fragments Per Kilobase Millon Mapped Reads) were then calculated to represent the expression level of genes in each sample. The protein-coding potential of transcripts was evaluated using the CNCI, CPC, PFAM, and CPAT analysis. Novel lncRNAs were identified as non-coding RNA in all four analyses. Conservative analysis of the identified novel lncRNAs was performed by PhastCons. RefSeq gene counts were normalized by trimmed mean of M value (TMM) method. DEGs were identified using Bioconductor package edgeR using criteria of fold change ≥2, FDR <0.05, and average coverage ≥1 in at least 1 sample. Empirical Bayes moderated statistics and corresponding *p* values were computed for comparisons and *p*-values were adjusted for multiple comparisons using the Benjamini–Hochberg procedure. Genes with an adjusted *p*-value of <0.05 were considered differentially expressed and defined as optimized data. The DE-lncRNA and mRNA between T1D and T2D were analyzed by the present data and our previous data.

### Construction of the Coding Non-Coding Gene Co-Expression Network and Correlation Analysis

To explore the association between lncRNAs and target mRNAs, a coding non-coding co-expression (CNC) network was constructed based on correlation analysis between DE-lncRNAs and mRNAs. Target mRNAs were selected with High Spearman correlation Coefficient (*p* ≥0.9) or with distance less than 50 kb. The network was constructed with Cytoscape (http://www.cytoscape.org) and STRING (https://string-db.org).

To obtain DE-lncRNA associated protein-coding genes, correlation analysis was performed by R studio (v1.2.1335). The expression level of fourteen DE-lncRNAs and all mRNAs were calculated the correlation co-efficient. Pearson’s correlations ranging from 0.3 to 0.5, 0.5 to 0.7 or >0.7 are classified as weak, moderate, and strong, respectively. P-value is less than 0.05.

### GO Term and KEGG Pathway Analysis

Differentially expression genes (DEGs) between T2D and healthy control were set to the Gene Ontology (GO) database to analyze the potential functions of lncRNAs and their associated mRNAs. The terms were supplied as annotation to genes and gene products. In this study, we mainly focused on the biological process (BP), cellular component (CC), and molecular function (MF) domains and a *P*-value of <0.05 was considered statistically significant. KEGG pathway analysis of DEGs was performed using the KOBAS online analysis database (available online: http://kobas.cbi.pku.edu.cn/). We analyzed the significantly differentially expressed co-expression mRNAs of lncRNAs as determined from CNC network.

### qPCR for Validation of Differential lncRNA in Expanded Cohort

A total of 21 lncRNAs were chosen to be validated further by qPCR in independently expanded samples of CTL (n = 36), T2D (n = 56), respectively. These 21 candidate lncRNAs were chosen based on the following criteria: 1) the biotype of both the lncRNA or antisense-lncRNA was included; 2) lncRNAs with no significant different expression between T2D and control were randomly selected; 3) both novel and known lncRNAs were included; 4) both up and downregulated DE-lncRNAs were included; and 5) the lncRNAs in the CNC network were chosen. After primer design and optimization of PCR conditions, 21 lncRNAs were tested and the information is presented in **Table S2**. The 9 DE-lncRNA between T1D and T2D were tested by 56 T2D and 11 T1D samples as well. cDNA was reverse transcribed by 5× All-In-One MasterMix (with AccuRT Genomic DNA Removal Kit) (Applied Biological Materials Inc., Canada) according to the manufacturer’s instructions. The qPCR was performed using EvaGreen 2× qPCR MasteMix—No dye (SYBR Green) (Applied Biological Materials Inc., Canada) and samples were amplified using the CFX Connect qPCR System (Biorad, Hercules, CA, USA). All experiments were conducted in triplicate and these triplicates were repeated three times. The 2^−ΔΔCT^ method was used to quantify the relative expression of each lncRNA, and β-actin was used as an internal control.

### Statistical Analysis

Statistical analysis was performed using SPSS 19.0 software (SPSS Inc., USA). All values were expressed as the mean ± SEM. Differences were considered statistically significant at *p <*0.05. Differential expression levels of lncRNA in the expanded samples were evaluated with the Mann–Whitney U test. Differences were considered statistically significant at *p <*0.05.

## Results

### Transcriptomic Profiles in Patients With Type 2 Diabetes

**Figure 1:**
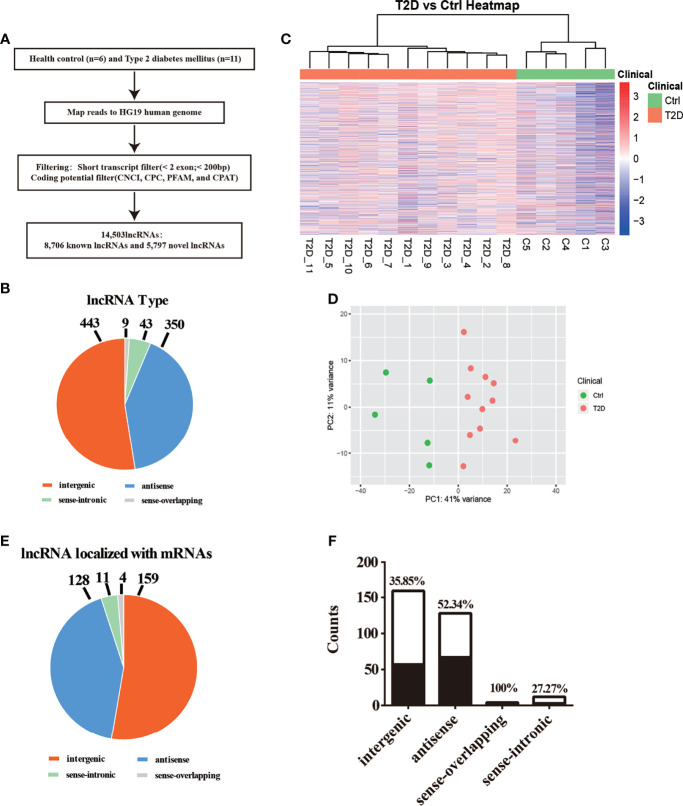
Transcriptomic landscape of T2D-lncRNAs. **(A)** A schematic illustration of the procedure used to identify and define lncRNAs in the leukocytes of patients with type 2 diabetes and to discover lncRNAs different between type 1 diabetes and type 2 diabetes. **(B)** Pie chart representations show the proportion of type 2 diabetes associated lncRNAs that are transcribed as antisense, intergenic, sense-overlapping, and sense-intronic lncRNAs, analyzed post-optimization. **(C)** Differential lncRNA expression profiles were hierarchical cluster analyzed and shown as a heatmap. **(D)** Principal component analysis shows similar results also presented as a heatmap. **(E)** The different classes of T2D-lncRNAs with significant differential expression derived from coding genes classified into intergenic, antisense, sense-overlapping, and sense-intronic lncRNAs according biotype. **(F)** The ratio of different classes T2D-lncRNA with significant co-expression mRNAs.

To determine the transcriptomic profiles of T2D patients, we evaluated 11 patients diagnosed T2D and 5 healthy volunteers as controls. The data process flow chart shown in **Figure 1A**. The information about the T2D participants is shown in **Table 1**, which demonstrates that most clinical characteristics in the discovery cohort and expanded cohort were not significantly different. Thus, the lncRNAs selected in the discovery cohort could be reliably validated in the expanded cohort.

**Table 1:** Clinical characteristics of included patients of type 2 diabetes in discovery cohorts (n=11) and in validation cohorts (n=56).

Items	Type 2 diabetes in discovery cohorts (n=11)	Type 2 diabetes in validation cohorts (n=56)	t-test/chi-square test (p value)
Age (years)	40.91±11.77	47.55±12.12	0.1098
Sex (male%)	90.91%	39.13%	0.1078
BMI (kg/m2)	25.63±4.26	27.68±5.50	0.1822
Fasting glucose (mmo/L)	7.55±1.98	8.08±3.08	0.4762
OGTT insulin (0h,μU/mL)	17.98±29.23	20.20±46.10	0.8628
OGTT insulin (2h,μU/mL)	57.24±75.06	61.52±78.30	0.8869
HbA1c	8.46±1.97	8.82±1.95	0.5904
Total cholesterol (mg/dL)	5.05±3.01	4.46±1.21	0.5375
Fasting triglycerides (mg/dL)	2.53±3.09	2.66±2.56	0.9021
HDL (mg/dL)	1.04±0.22	1.01±0.39	0.7092
LDL (mg/dL)	2.66±0.66	2.65±0.92	0.9663

We first described the transcriptomic profiles of T2D to identify critical genes and lncRNAs in the T2D patients. In total, 14,503 lncRNAs and 17,487 mRNAs (8,331 upregulated and 9,156 downregulated) were detected in leukocytes from T2D patients. Of these, 8,706 lncRNAs have been registered in databases (defined as known), namely, 4,821 upregulated and 3,885 downregulated lncRNAs. Additionally, 5,797 lncRNAs were identified for the first time (defined as novel) with 3,663 upregulated and 2,134 downregulated. After optimization using an adjusted *p*-value (threshold of *p <*0.05), we identified 845 significantly differentially expressed (DE) lncRNAs (T2D-lncRNAs) (**Table S3**), among which 260 were downregulated and 585 were upregulated (**Figure S1A**). The top 20 lncRNAs are shown in **Table 2**. Further, 1,305 mRNAs (T2D-mRNAs) were found in T2D by similar optimization, with 559 downregulated and 746 upregulated (**Figure S1A**). Of the DE-lncRNAs, 350 (41.4%) were antisense, 443 (52.4%) were intergenic (lincRNA), and 52 belonged to sense-overlapping (5.09%), and sense-intronic (1.06%) lncRNAs (**Figure 1B** and **Table S3**). Both T2D-lncRNA and T2D-mRNA were clearly distinguished in T2D patients and healthy controls by hierarchical clustering (**Figure 1C** and **Figure S1A**) and principal content analysis (**Figure 1D** and **Figure S1B**). Taken together, the difference between healthy controls and T2D patients was significantly reflected by both the expression of protein coding genes and hundreds of lncRNAs in the leukocytes.

**Table 2:** The top 20 lncRNA with significantly differential expression in white blood cell from type 2 diabetes (T2D) and health control (C).

Gene	C normalize	T2D normalize	Log2 Fold Change	FDR	Up/Down	Biotype
ENSG00000267257	19.44	0.55	-5.13	8.5E-07	down	antisense
ENSG00000276107	39.88	3.37	-3.56	1.79E-06	down	sense_intronic
MSTRG.103146	598.79	214.23	-1.48	1.74E-05	down	antisense
MSTRG.19495	0.65	12.42	4.26	5.77E-05	up	linc
ENSG00000273338	104.62	15.05	-2.8	6.36E-05	down	antisense
MSTRG.180057	0.83	13.65	4.04	7.37E-05	up	linc
MSTRG.22781	3.1	26.47	3.1	8.32E-05	up	antisense
MSTRG.161229	27.29	128.52	2.24	8.34E-05	up	antisense
MSTRG.180437	201.79	585.68	1.54	9.34E-05	up	linc
MSTRG.180334	0.6	11.82	4.31	9.76E-05	up	linc
MSTRG.182419	88.48	13.76	-2.69	9.78E-05	down	linc
MSTRG.106807	2940.94	1210	-1.28	0.000101	down	antisense
MSTRG.195300	54.62	0.59	-6.53	0.000105	down	linc
MSTRG.62902	0.64	11.46	4.17	0.000105	up	antisense
MSTRG.125714	8.73	47.42	2.44	0.000106	up	antisense
MSTRG.10587	4.64	28.06	2.59	0.000123	up	antisense
MSTRG.80841	1.05	12.84	3.61	0.000139	up	linc
MSTRG.181523	1.29	18	3.8	0.000185	up	antisense
ENSG00000260244	281.53	76.87	-1.87	0.000222	down	sense_overlapping
MSTRG.183281	335.1	117.4	-1.51	0.000315	down	antisense

We further analyzed the different classes of T2D-lncRNAs derived from coding genes. In the T2D-lncRNAs dataset, 302 lncRNAs were localized with their mRNAs, but only 131 of lncRNAs showed significantly altered expression levels of their localized mRNA. The biotype of the 302 lncRNA including intergenic (159 lncRNAs), antisense (128 lncRNAs), sense-overlapping (4 lncRNAs), and sense-intronic (11 lncRNAs) are shown in **Figure 1E**. Sense-overlapping lncRNAs showed the highest ratio (100%, 4/4) of significant co-expression in each biotype group (**Figure 1F**).

### Features of Novel lncRNAs Identified in Patients With Type 2 Diabetes

**Figure 2:**
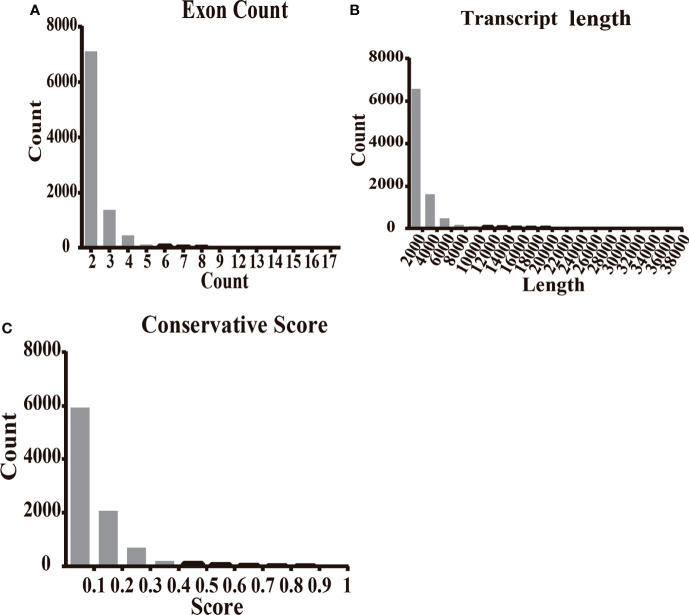
The features of novel lncRNAs in T2D patients compared with healthy controls. **(A)** The transcripts of novel lncRNAs were mainly distributed in 2, 3, and 4 exons. **(B)** The largest amount of novel lncRNA transcripts distributed as the length less than 2,500 nt. **(C)** The transcripts of novel lncRNAs distributed at the conservation score range. The ratio of transcript with conservation scores less than 0.1 was 61.3%.

The characteristics of 9,114 transcripts (some lncRNAs have more than one transcript) identified in T2D patients were first disentangled as novel lncRNAs. Most novel lncRNA transcripts harbored 2 exons (7,106/9,114, 77.97%) (**Figure 2A**). The lengths of most novel lncRNAs (6,570/9,114, 72.09%) were less than 2,000 bp (**Figure 2B**). The results of conservation analysis of the novel lncRNAs in humans indicated that more than half (5,587/9,114, 61.3%) had low conservation scores (CS less than 0.1) (**Figure 2C**) and 6.77% of lncRNA transcripts (617/9,114) had CS less than 0.01 between human and other species. Analysis of the distribution of transcripts on chromosomes demonstrated that novel lncRNAs were mainly distributed on chr1, chr2, chr3, chr5, chr6, and chr7 and that most lncRNAs with low CS in humans were from the same chromosome (**Figure S2**).

### CNC Network of lncRNAs and mRNAs in Type 2 Diabetes

**Figure 3:**
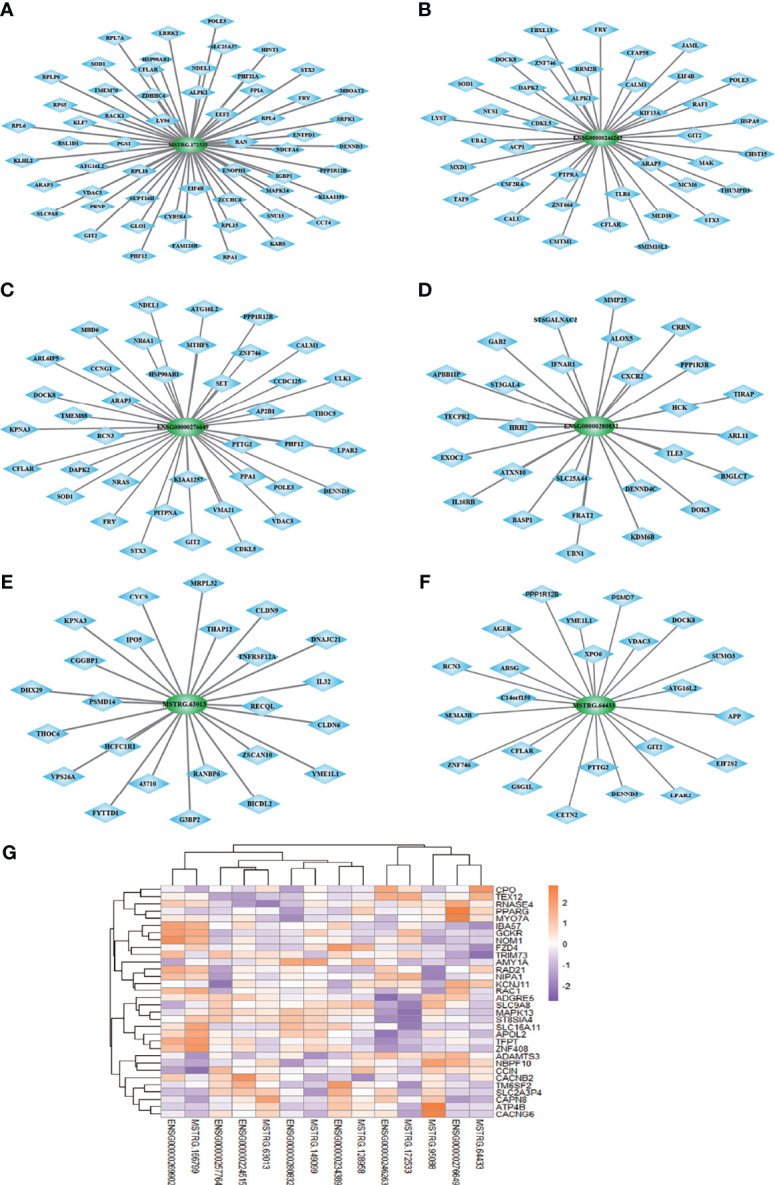
**(A–F)** The co-expression network of 6 lncRNAs validated in expanded cohort which selected from top 10 lncRNA with most related mRNA. **(G)** Correlation analysis was performed to obtain DE-lncRNA associated protein-coding genes. Pearson’s correlations ranging from 0.3 to 0.5, 0.5 to 0.7 or >0.7 are classified as weak, moderate and strong, respectively. P-value is less than 0.05.

We structured co-expression networks to determine if lncRNAs are associated with one to dozens of lncRNAs and mRNAs. There were 1,076 genes involved in our co-expression networks consisting of 618 lncRNAs and 458 mRNAs (**Figure S3A**). In total, 21 lncRNAs and 117 mRNAs harbored more than 10 related genes in the CNC network. The top 10 lncRNAs and mRNAs with the number of genes to which they were related are shown in **Table 3**. The lncRNAs used for further validation and their CNC network are shown in **Figures 3A**–**F**. LncRNA MSTRG.172533 was the top-ranking lncRNA harboring 57 genes in its network, among which RACK1 (16), SLC9A8 (17), and others have been reported as related to diabetes. The mRNA RAC1 harbored 37 genes and ranked first in CNC analysis, which has been explored in diabetes (18), diabetic retinopathy (19), and so on. We also performed correlation analysis to obtain DE-lncRNA associated protein-coding genes. As shown in **Figure 3G**, Pearson’s correlations ranging from 0.3 to 0.5, 0.5 to 0.7 or >0.7 are classified as weak, moderate, and strong, respectively. P-value is less than 0.05.

**Table 3:** The top 10 lncRNAs and mRNAs with numbers of related genes in co-expression network.

Gene	Biotype	Target gene counts
MSTRG.172533	lncRNA	57
MSTRG.118864	lncRNA	52
ENSG00000276649	lncRNA	39
ENSG00000246263	lncRNA	39
RAC1	mRNA	37
ENSG00000279463	lncRNA	35
POLR2K	mRNA	32
APP	mRNA	32
MAPK3	mRNA	32
RHOA	mRNA	30

Taken together, these results confirm that lncRNA and mRNA in leukocytes are important in type 2 diabetes and these association between lncRNAs and their related genes should be further investigated.

### GO and KEGG Pathway Analyses

**Figure 4:**
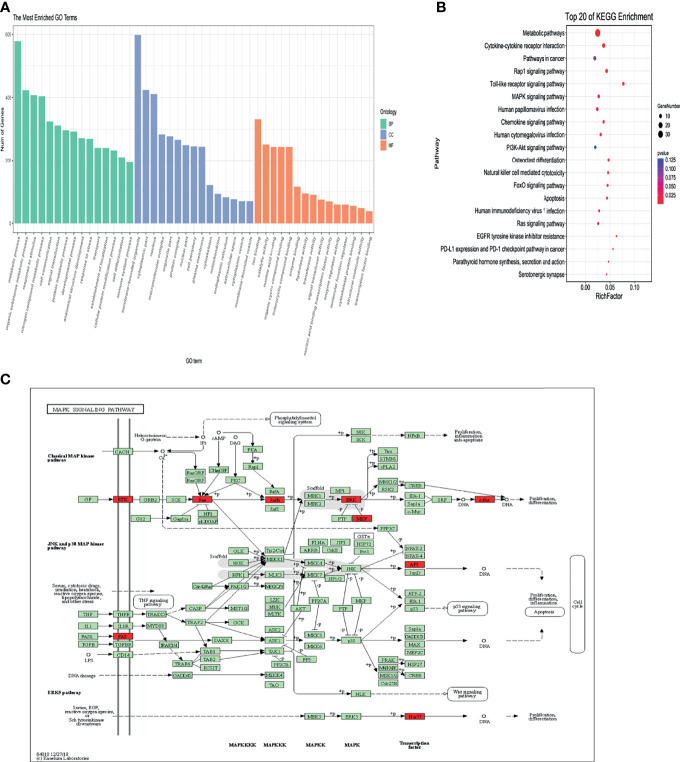
Bioinformation analysis by enrichment analysis of pathways and GO terms for T2D-lncRNA and T2D-mRNA using optimized data. **(A)** Gene ontology analysis of lncRNA-associated mRNAs of T2D in biological processes, molecular functions, and cellular components. **(B)** KEGG pathway analysis of lncRNA-associated mRNAs of T2D. **(C)** Differentially expressed DEGs enriched in MAPK signaling pathway.

We further applied the Gene Ontology (GO) enrichment analysis to classify the DE-lncRNAs associated-mRNAs of T2D into three main categories, namely, biological process, molecular function, and cellular component (**Figures 4A, B**). In the biological process group, the DEGs were mainly enriched in metabolic process, response to stimulus, cell communication, nitrogen compound metabolic process and immune system process are among the top 15 items (**Figure 4A**). Under the molecular function category, ion binding, catalytic activity, nucleic acid binding, and organic cyclic compound binding showed the highest percentages (**Figure 4A**). Next, we compared 6 T2D patients with family histories and 5 samples without family histories to 5 healthy controls, respectively. Their overlapped genes were undertaken GO analysis. It was very similar to the previous one (**Figure S3**). These data indicate that genetic factors (e.g., family history) may not have important impacts on lncRNA functions in T2D. We further performed KEGG pathway analysis. As shown in **Figure 4B**, the DEgene-enriched pathway included the metabolic pathways, Rap1 signaling pathway, Toll-like receptor signaling pathway, MAPK pathway and PI3K-Akt signaling pathway. Among them, 9 DEGs were enriched in MAPK pathway, which participate in the development of pathological traits resulting from excessive caloric intake and obesity that cause metabolic syndrome and type 2 diabetes (**Figure 4C**).

### Measurement of Chosen lncRNAs in Validation Groups

**Figure 5:**
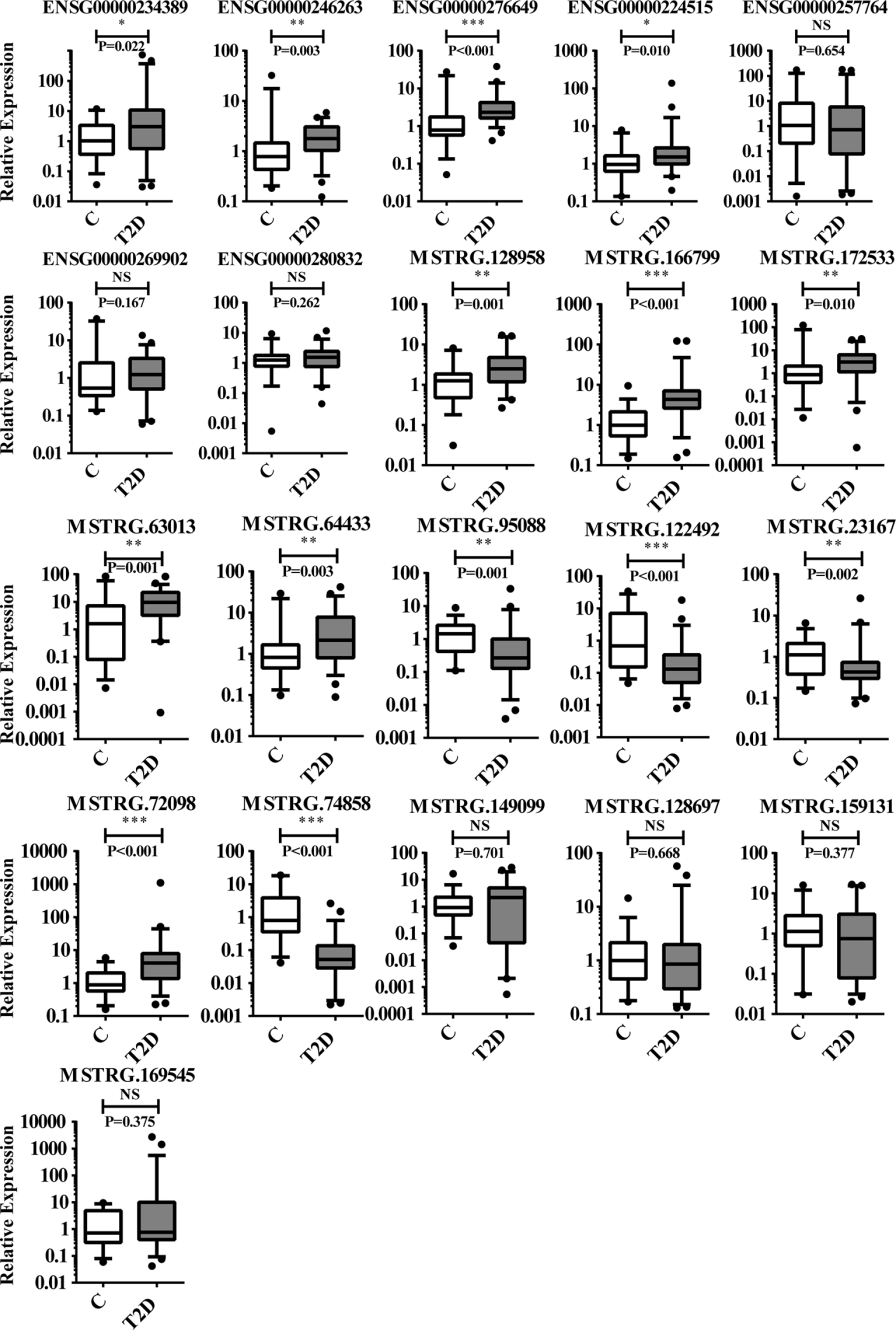
Results of 21 lncRNAs further confirmed in the validation cohorts by qPCR. There are 14 lncRNAs that showed significant difference in expression levels between T2D patients (n = 56) and healthy controls (n = 36). NS, no significant difference; “*” indicated significant difference with *p <* 0.05, “**” indicated *p < *0.01 and "***" indicated *p <* 0.001.

**Figure 6:**
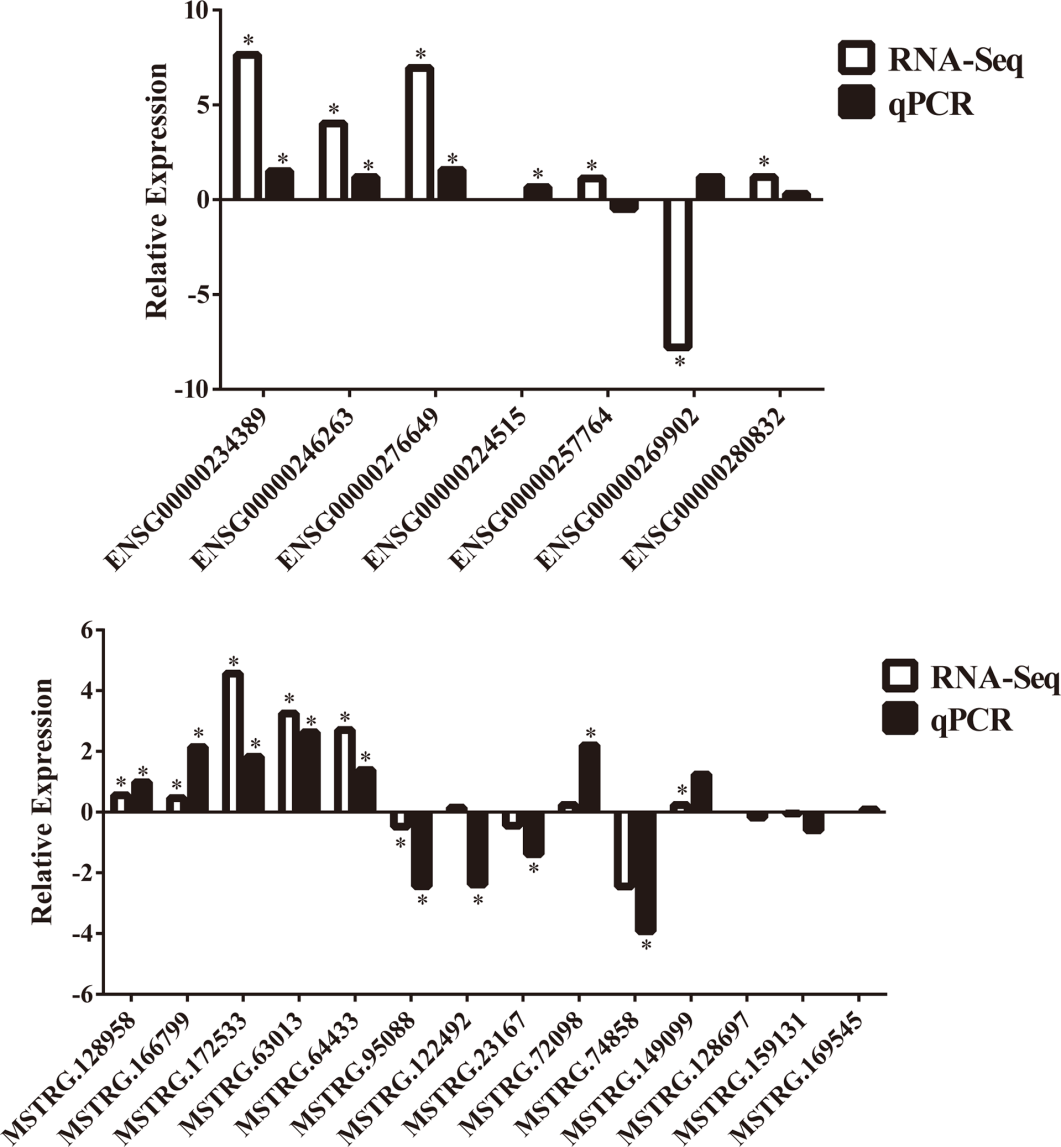
Comparisons of trends between RNA sequencing data and further confirmation of 21 lncRNAs in the validation cohorts by qPCR. “*” indicated significant difference in RNA sequencing data or validation cohorts by qPCR.

To further confirm the DE-lncRNAs, we independently measured 21 lncRNAs in the validation groups of T2D patients (n = 56) and healthy controls (n = 36) by qPCR. The expression levels of these lncRNAs in the validation cohort are shown in **Figure 5**. Fourteen of 21 (66.67%) lncRNAs displayed obviously different expression levels between T2D and healthy control groups; 4 lncRNAs were downregulated and the others were upregulated in T2D. Ten novel lncRNAs and 4 known lncRNAs were confirmed as positive in the expanded cohort. Among them, 8 lncRNAs belonged to the lincRNA, whereas 5 lncRNAs belonged to the antisense (**Table 4**). We then compared the validation results and sequencing data, which showed that most lncRNAs (85.71%, 18/21) in the expanded group displayed similar trends as the sequencing data (**Figure 6**). Particularly, 9 lncRNAs exhibited the same significantly positive results as observed in the sequencing data. Furthermore, we compared the validation positive ratio by categorizing these lncRNAs with their features (**Table 4**). The results demonstrated that 4 of 8 lncRNAs (50%) without significant differences in the sequenced data were significantly different in the validation test. The validation-positive ratios of the known- and novel-lncRNAs were 57.14 and 71.43%, respectively, in the validation cohorts (**Table 4**). Six known and 7 novel lncRNAs with significant differences in the sequencing data demonstrated positive ratios of 50 and 85.71%, respectively (**Table 4**). Only 1 of 2 lncRNAs with no expression in either the control or T2D group in the sequencing data displayed significant differences in the expanded cohort (**Table 4**). When validation-positive lncRNAs were analyzed by biotype, the results showed that the positive ratios in lincRNA and antisense lncRNA were 61.54% (8/13) vs. 71.43% (5/7). The positive ratios in the lncRNAs with and without predicted mRNAs were 88.89% (8/9) and 50% (6/12), respectively (**Table 4**).

**Table 4:** The results of analysis by the categorical features of 21 lncRNAs which were chosen in validation cohorts. “*” marked the validation positive lncRNA.

Categorical features	LncRNA	Ratio of lncRNA with significant difference
No significant difference in sequencing cohorts	MSTRG.169545, MSTRG.23167*, MSTRG.72098*, MSTRG.74858*, ENSG00000224515*, MSTRG.128697, MSTRG.122492*, MSTRG.159131	4/8 (50.0%)
No expression in either group of sequencing cohorts	ENSG00000224515*, MSTRG.128697	1/2 (50.0%)
Known lncRNA with significant difference in sequencing cohorts	ENSG00000234389*, ENSG00000246263*, ENSG00000257764, ENSG00000269902, ENSG00000276649*, ENSG00000280832	3/6 (50.0%)
Novel lncRNA with significant difference in sequencing cohorts	MSTRG.128958*, MSTRG.149099, MSTRG.166799*, MSTRG.172533*, MSTRG.63013*, MSTRG.64433*, MSTRG.95088*	6/7 (85.71%)
Known lncRNA	ENSG00000234389*, ENSG00000246263*, ENSG00000257764, ENSG00000269902, ENSG00000276649*, ENSG00000280832, ENSG00000224515*	4/7 (57.14%)
Novel lncRNA	MSTRG.128958*, MSTRG.149099, MSTRG.166799*, MSTRG.172533*, MSTRG.63013*, MSTRG.64433*, MSTRG.95088*, MSTRG.169545, MSTRG.23167*, MSTRG.72098*, MSTRG.74858*, MSTRG.128697, MSTRG.122492*, MSTRG.159131	10/14 (71.43%)
LincRNA	ENSG00000269902, MSTRG.149099, MSTRG.169545, MSTRG.172533*, MSTRG.23167*, MSTRG.63013*, MSTRG.64433*, MSTRG.72098*, MSTRG.74858*, MSTRG.95088*, MSTRG.128697, MSTRG.122492*, MSTRG.159131	8/13 (61.54%)
Antisense lncRNA	ENSG00000246263*, ENSG00000257764, ENSG00000276649*, ENSG00000280832, ENSG00000224515*, MSTRG.128958*, MSTRG.166799*	5/7(71.43%)
LncRNA with predicted mRNA	ENSG00000234389*, ENSG00000246263*, ENSG00000276649*, ENSG00000280832, MSTRG.149099, MSTRG.166799*, MSTRG.172533*, MSTRG.63013*, MSTRG.64433*	7/9 (77.78%)
LncRNA without predicted mRNA	ENSG00000257764, ENSG00000269902, ENSG00000224515*, MSTRG.128958*, MSTRG.95088*, MSTRG.169545, MSTRG.23167*, MSTRG.72098*, MSTRG.74858*, MSTRG.128697, MSTRG.122492*, MSTRG.159131	7/12 (58.33%)

We also sought to identify potential orthologs of 21 lncRNAs by comparing their sequences with previously identified murine lncRNAs. Based on pairwise genomic alignments, we found that 11 lncRNA sequences (52.38%) harbored orthologs in mouse genomic sequences without annotation, whereas the other 10 lncRNAs were had no orthologs in mice (**Table 5**). Of the 14 validation-positive lncRNA, 5 (35.71%) exhibited orthologous sequences in the mouse genome (**Table 5**). Thus, the validated lncRNAs exhibited evolutionary conservation in humans and should be further investigated to determine their relationship with the human T2D epigenetic mechanism or as biomarkers.

**Table 5:** The information of 14 lncRNAs positively validated in the expanding cohort in present study and their potential orthologous sequences comparing with mouse data.

LncRNAs	Novel/known	The log2 value in the RNA-seq data	Significant difference in validation cohort	Orthologous sequence with mouse
ENSG00000224515	known	–	yes	Not found
ENSG00000234389	known	1.79	yes	Not found
ENSG00000246263	known	1.03	yes	Not found
ENSG00000257764	known	3.55	no	Not found
ENSG00000269902	known	-1.6	no	Not found
ENSG00000276649	known	1.77	yes	Not found
ENSG00000280832	known	1.63	no	Not found
MSTRG.122492	novel	0.6	yes	chr3:58,478,307-58,478,532
MSTRG.128697	novel	–	no	Not found
MSTRG.128958	novel	5.21	yes	chr5:66,016,825-66,017,169
MSTRG.149099	novel	5.32	no	Not found
MSTRG.159131	novel	-0.65	no	Not found
MSTRG.166799	novel	3.88	yes	chr6:39,016,934-39,045,231
MSTRG.169545	novel	1.44	no	chr14:70,790,722-70,791,594
MSTRG.172533	novel	1.99	yes	Not found
MSTRG.23167	novel	-1.12	yes	chr19:35,211,979-35,262,091
MSTRG.63013	novel	2.66	yes	chr17:23,644,824-23,660,240
MSTRG.64433	novel	2.11	yes	chr7:126,093,191-126,095,342
MSTRG.72098	novel	2.06	yes	chr11:95,397,976-95,398,838
MSTRG.74858	novel	-1.51	yes	chr11:121,806,938-121,808,036
MSTRG.95088	novel	-2.66	yes	chr2:45,237,395-45,239,069

### DE-lncRNA Between Type 1 and 2 Diabetes

**Figure 7:**
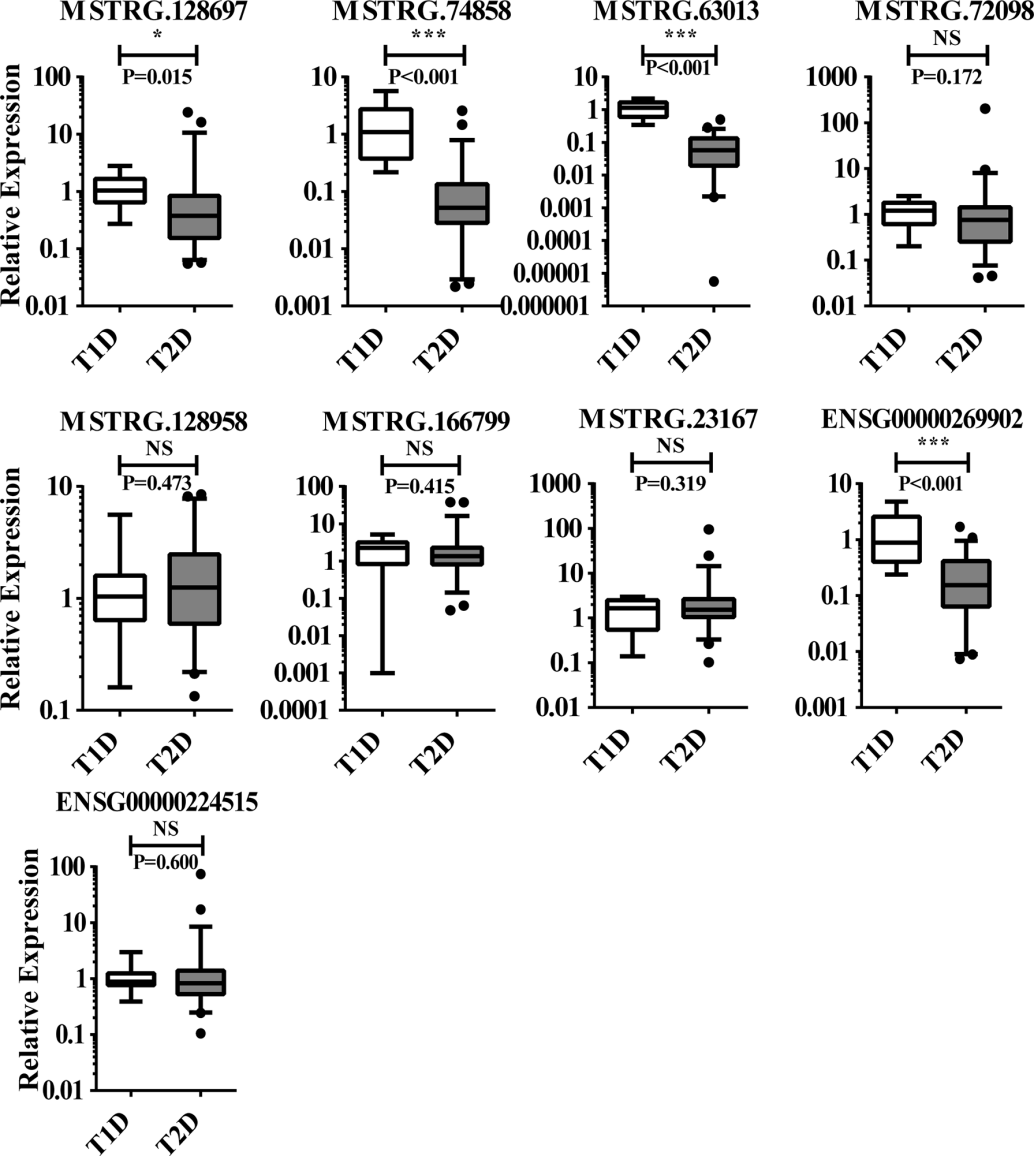
The 9 lncRNAs tested in T1D (n = 11), and T2D (n = 56) using qPCR, of which 4 lncRNAs with significant difference and 6 with non-significant difference. NS, no significant difference; “*” indicated significant difference with *p* < 0.05 and “***” indicated *p* < 0.001.

To further screen for lncRNAs differentially expressed in T1D and T2D with potential as diagnostic markers for distinguishing T1D and T2D, the transcriptome sequencing cohort comprised of 11 patients diagnosed with T2D and 6 patients diagnosed with T1D (Accession number: GSE130279) was analyzed. Only 10 lncRNAs showed significant difference between the two groups, with 7 upregulated and 3 downregulated lncRNAs (**Table S5**); 15 mRNAs with 6 upregulated and 9 downregulated mRNAs (**Table S6**). We independently measured 9 lncRNAs (4 showed significant differences in sequencing data, **Table S7**) by qPCR in validating T1D (n = 11), and T2D (n = 56). We found 4 lncRNAs (MSTRG.128697, MSTRG.74858, MSTRG.63013, and ENSG00000269902) showed significantly differential expression between T1D and T2D. Their expression levels in the discovery cohort and validation cohort are shown in **Figure 7**. Thus, this panel of 4 lncRNAs may be valuable as diagnostically distinguishable markers in T1D and T2D.

## Discussion

LncRNAs participate in the epigenetic regulation of various diseases by altering the expression of lncRNA target genes and displaying clear clinical significance (20). An increasing number of studies have demonstrated that causal variants of diabetes-related lncRNA are significantly enriched in the islet regions and transcription factor-binding sites (21). However, studies aimed at identifying lncRNAs involved in T2D in circulating leukocytes are very limited. A very recent report showed that circulating lncRNAs were aberrantly expressed in T2D by microarray analysis, indicating their potential roles in chronic inflammation and insulin resistance (14). We predicted that in addition to islet beta cells, lncRNAs may play a regulatory role in peripheral blood cells such as leukocytes and alter cellular phenotypes and disease risk. In the present study, we determined the full profile of circulating lncRNA and mRNA in T2D patients to describe their characteristics and differential expression compared to healthy controls. In total, 14,503 lncRNAs were identified in T2D leukocytes and 5,797 lncRNAs were defined as novel lncRNAs. Adjusted data showed that 845 lncRNAs were significantly different between T2D patients and healthy controls. We report more data than in previous studies in which the peripheral blood of patients with diabetes was screened for associated lncRNAs by microarrays (14), but fewer than those reported by transcriptomic sequencing or microarrays in beta cells (6). Here, we constructed DE-lncRNA and DE-mRNA networks to explore the biological functions of lncRNAs during the development of diabetes mellitus. There were 1,076 genes involved in our CNC results, among which 21 lncRNAs and 117 mRNAs harbored more than 10 related genes. Further analysis indicated that the top lncRNA (MSTRG.172533) harbored 57 genes in its network. Genes in this network such as RACK1 (16), SLC9A8 (17), PPIA (22), and others have been explored in diabetes. These results strongly support that circulating leukocytes from T2D harbor enriched lncRNAs and may represent important targets for further diabetes research. Newly obtained data of lncRNAs and mRNAs in our study may extend the knowledge of molecular alterations in the T2D transcriptome.

Murine disease models are often chosen as the first alternative to avoid highly heterogeneous human genetic backgrounds and low RNA integrity from cadaveric islets in exploring the role of lncRNAs (21). However, considering that the evolutional conservation of lncRNA is much lower than that of mRNA between mice and humans, it is important to evaluate whether mouse models are useful in this case. In fact, notable species differences in lncRNA expression have been reported in comparisons of the transcriptional landscape of mouse and human beta cells (23). It was reported that most adipose-enriched lincRNAs (~85%) were not conserved in mice, and as few as 15% of human lincRNA loci contain syntenic non-coding transcripts expressed in mouse (24). It has putatively been confirmed that there are mouse orthologues for 70% of human lncRNAs but only 47% have been confirmed by RNA-seq (6). Approximately 85% of human macrophage lincRNAs are not expressed in mice, and only 24% show significant sequence conservation between humans and mice (11). These studies indicate that although mouse diabetic models and their lncRNAs are convenient for research, there are some clear barriers and limitations to the conclusions that can be drawn from these data because of the lack of sequence conservation. In the current study, we analyzed the evolutionary conservation of novel lncRNAs and 14 positively validation lncRNAs. Among the novel lncRNAs, 61.3% had a conservation score of less than 0.1, indicating that they exhibited high evolutionary conservation in humans. Of the set of validated 14 lncRNAs, only 35.71% (5 lncRNAs) had orthologs in mice with no further annotation. Thus, these validated 14 lncRNAs can be used directly in clinical research without considering the conservation problem. Therefore, the large amounts of lncRNAs and mRNAs obtained from convenient circulating leukocytes of T2D patients in the present study contribute valuable information based on an urgent need for human data.

To confirm and understand lncRNAs in T2D more detail, we validated 21 lncRNAs, 14 lncRNAs (14/21, 66.67%) displayed expression levels that were significantly different between T2D patients and healthy controls. Compared to the identification of lncRNAs in osteoarthritis chondrocytes (11/16, 68.75%) (25), in human coronary artery smooth muscle and endothelial cells (21/31, 67.74%) (26), and in blood samples from patients with diabetic neuropathy (2/6, 33.33%) (27), the detection rate we describe here is comparable. We found that the positive validation ratio of lncRNAs with predicted mRNAs (77.78%) in CNC was higher than those without predicted mRNAs (58.33%). The novel lncRNAs showed a higher positive validation ratio than known lncRNAs. These results indicate that candidate lncRNAs for validation can be chosen from a wider range, particularly from lncRNAs in the CNC.

LncRNAs can be classified into two large groups: lincRNAs with no direct relationship with protein-coding genes or those partially overlapping the protein coding sequences as sense- or antisense transcripts (28). When choosing candidate lncRNAs, the analysis of antisense-lncRNAs in peripheral blood mononuclear cells and the spinal cord have been prioritized (29). LincRNAs were first characterized to play important roles in macrophage activation in cardiometabolic diseases in human genetic studies (11). We found that lincRNAs exhibited lower validation positive ratios (61.54%) than antisense lncRNAs (71.43%). Nevertheless, both were comparable. Thus, our work is a reminder that it would be a better choice to investigate T2D in terms of both lincRNA and antisense-lncRNAs, as T2D has been considerably understudied compared to lncRNAs in general. In the 14 positively validated lncRNAs, 7 lncRNAs had predicted mRNA targets related to 144 genes (**Table S8**); of these, many genes have been confirmed as related to diabetes. For instance, in the network of lncRNA MSTRG.63013, 24 related genes, including G3BP2 (30), and others may affect diabetes development. The rest 7 lncRNAs lacked predicted mRNA targets, which may be because of our currently limited understanding of the human genome (or those of other animals). As a pioneering study, our results can be used to investigate the regulatory function of these lncRNAs in T2D in the future.

LncRNAs have been reported as disease biomarkers in diagnosis, prognosis, or therapeutic targeting because of their sensitivity and convenience (31). As the most common diabetes subtypes, type 1 and 2 diabetes are difficult to diagnose in many cases. Current methods include evaluation of clinical symptoms, C-peptide, and antibodies or other auto-immunobiomarkers related T1D. However, these indicators are not sufficiently specific and sensitive to meet the diagnosis requirement. Therefore, exploring new molecular biomarkers for accurately diagnosing type 1 and 2 diabetes is urgent. In fact, it was reported that lncRNAs are modulated during the development of T1D (32). Thus, we identified lncRNAs showing differential expression between type 1 and 2 diabetes which may be useful as distinguishing markers. We screened 4 lncRNAs that were significantly different expressed in circulating leukocytes from patients with type 1 and 2 diabetes. This panel may be used to develop a convenient and efficient molecular method for distinguishing type 1 and 2 diabetes in clinical practice.

There were several limitations to this study. First, the expanded cohort number was limited, particularly the number of T1D patients. This is because we grouped the samples very critically and collected samples without any treatment over a short term. Second, leukocytes may not completely represent the circulating conditions. Therefore, we plan to screen lncRNAs in circulating exosomes, which are extracellular vesicles that may play more important roles in regulating diabetes; these results will be compared to the lncRNAs identified in leukocytes in the current study. Finally, we did not investigate the relationship of lncRNAs and their regulatory or predicted mRNAs, although some lncRNAs and mRNAs were in the CNC. These regulatory relationships should be further analyzed.

In conclusion, the present RNA sequencing work identified thousands of lncRNAs and mapped their expression profiles in T2D. The many novel non-coding RNAs identified may function as key modulators of T2D. Importantly, the leukocyte specificity found for the 14 lncRNAs identified (together with other data presented) may be useful for future functional studies, and a set of 4 DE-lncRNAs may aid in accurately diagnosing type 1 and 2 diabetes.

## Data Availability Statement

The datasets presented in this study can be found in online repositories. The names of the repository/repositories and accession number(s) can be found below: NCBI Gene Expression Omnibus (https://www.ncbi.nlm.nih.gov/geo): GEO accession, GSE134594.

## Ethics Statement

The studies involving human participants were reviewed and approved by the Ethics Committee of the Chinese PLA General Hospital (Permitted No. S2016-147-03). The patients/participants provided their written informed consent to participate in this study.

## Author Contributions

All authors listed have made a substantial, direct, and intellectual contribution to the work and approved it for publication.

## Funding

This study was supported by the National Natural Science Foundation of China (Nos. 31970512, 32070531 and 31872308).

## Conflict of Interest

The authors declare that the research was conducted in the absence of any commercial or financial relationships that could be construed as a potential conflict of interest.

## Publisher’s Note

All claims expressed in this article are solely those of the authors and do not necessarily represent those of their affiliated organizations, or those of the publisher, the editors and the reviewers. Any product that may be evaluated in this article, or claim that may be made by its manufacturer, is not guaranteed or endorsed by the publisher.

## Supplementary Material

Supplementary Figure 1
**(A)** Differentially expressed lncRNAs (T2D-lncRNAs) and type 2 diabetes mRNA were identified from a Volcano plot showing data from type 2 diabetes patient relative to healthy controls. The vertical black lines correspond to 2-fold up and downregulations, respectively; and the horizontal black line represents a *p-*value of 0.05. The red and green points in the plots represent the differentially expressed genes with statistical significance for upregulation and downregulation of lncRNA and mRNA, respectively. **(B)** Differential mRNA expression profiles were hierarchical cluster analyzed and shown as a heatmap. **(C)** Principal component analysis showed similar results as presented in heatmap.

Supplementary Figure 2 The conservation of novel lncRNAs on chromosomes.

Supplementary Figure 3
**(A)** Co-expression networks of lncRNA-mRNA involved 1076 genes, consisted by 618 lncRNAs and 458 mRNAs. Venn plot for the differentially expressed lncRNAs **(B)** and mRNAs **(C)** between T2DM with- and without- family history samples. **(D)** Go analysis for the overlapped genes of T2DM with- and without family history samples.
