# Supplementary figures and images for: Profile Screening of Differentially Expressed lncRNAs of Circulating Leukocytes in Type 2 Diabetes Patients and Differences From Type 1 Diabetes
Source: Front Endocrinol (Lausanne). 2022 Jan 10;12:690555. doi: 10.3389/fendo.2021.690555 (PMC8786112; doi:10.3389/fendo.2021.690555)

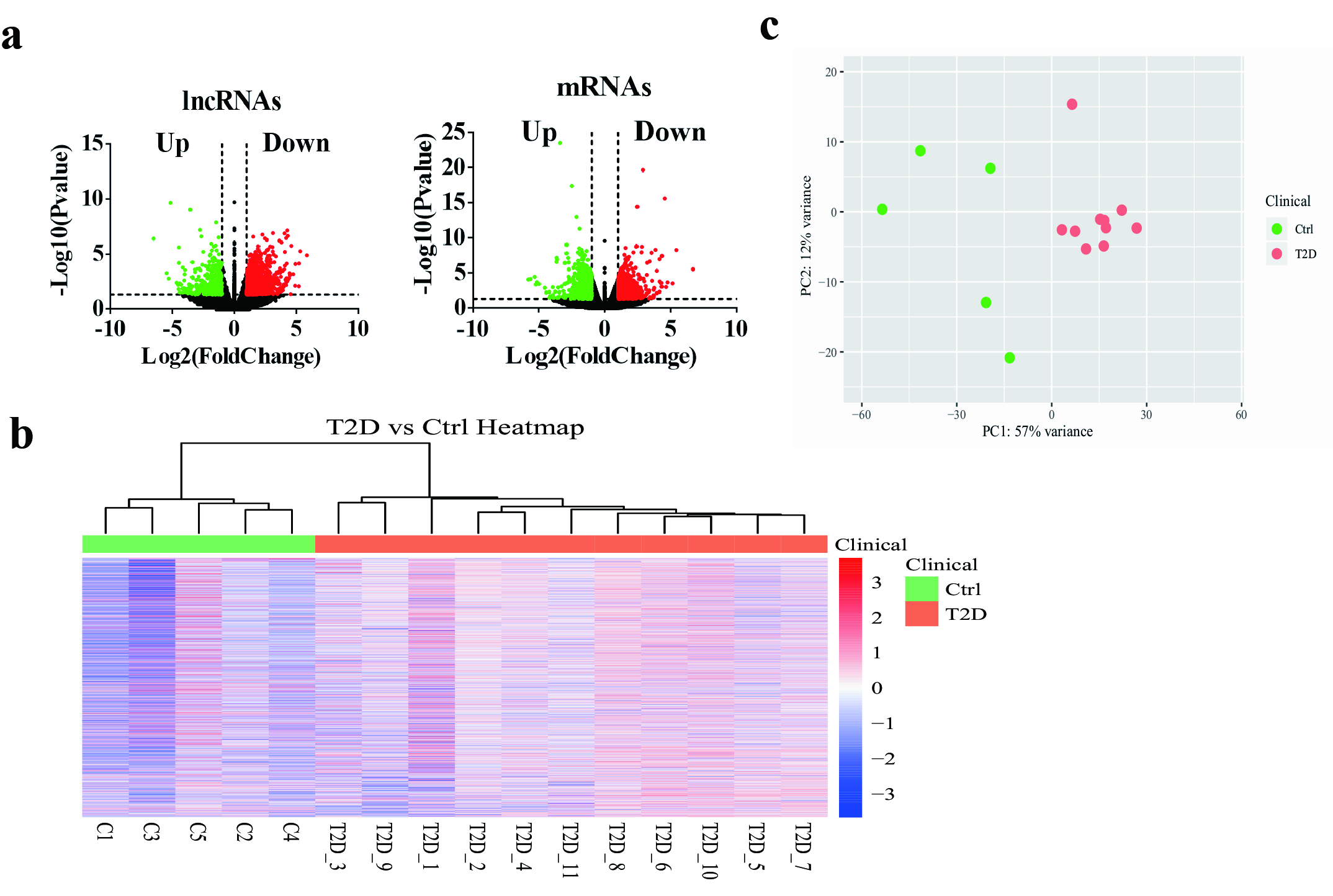

Supplement: Supplementary Figure 1 — (A) Differentially expressed lncRNAs (T2D-lncRNAs) and type 2 diabetes mRNA were identified from a Volcano plot showing data from type 2 diabetes patient relative to healthy controls. The vertical black lines correspond to 2-fold up and downregulations, respectively; and the horizontal black line represents a p-value of 0.05. The red and green points in the plots represent the differentially expressed genes with statistical significance for upregulation and downregulation of lncRNA and mRNA, respectively. (B) Differential mRNA expression profiles were hierarchical cluster analyzed and shown as a heatmap. (C) Principal component analysis showed similar results as presented in heatmap. [file Image_1.tif]

IncRNA mRNA

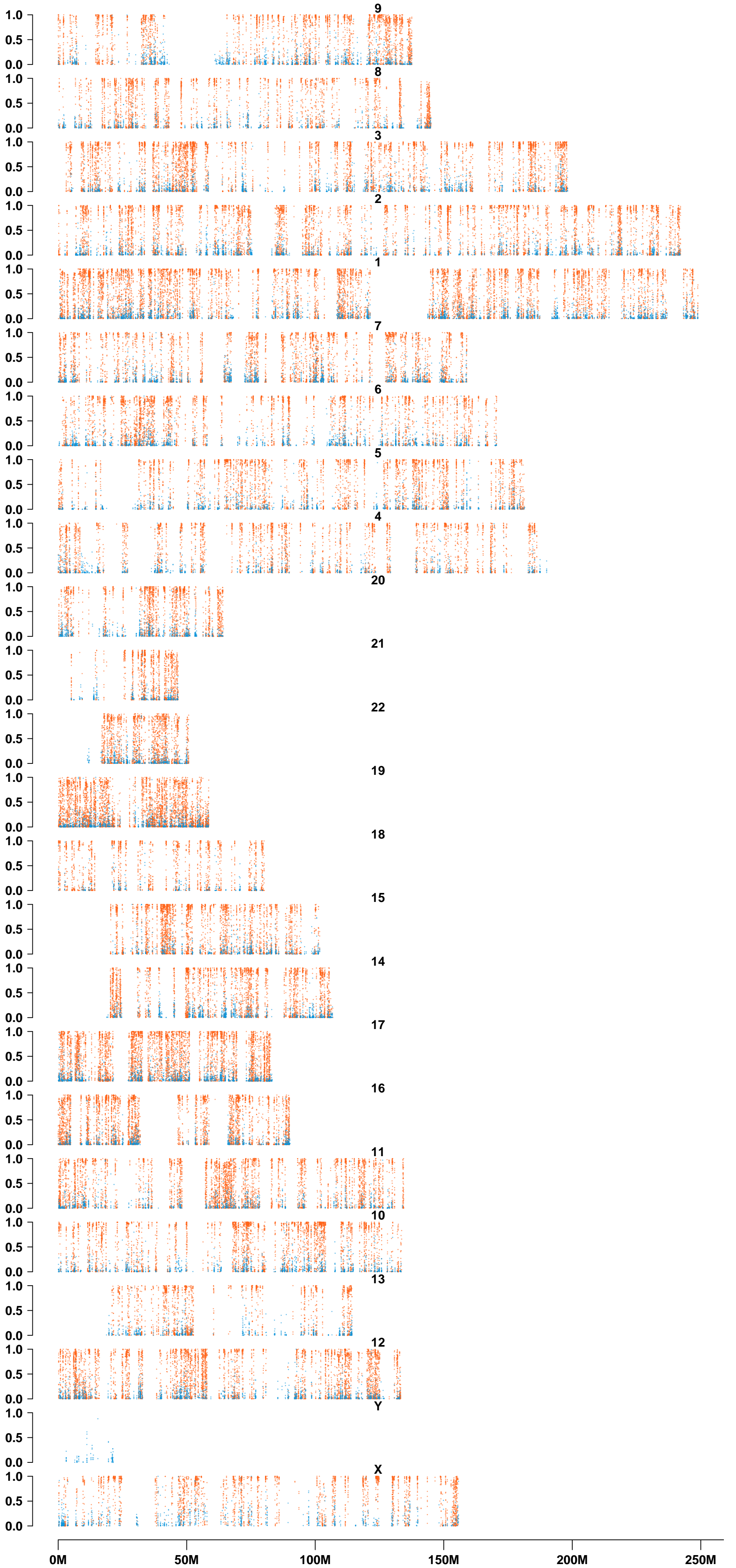

Supplement: Supplementary Figure 2 — The conservation of novel lncRNAs on chromosomes. [file Image_2.pdf]

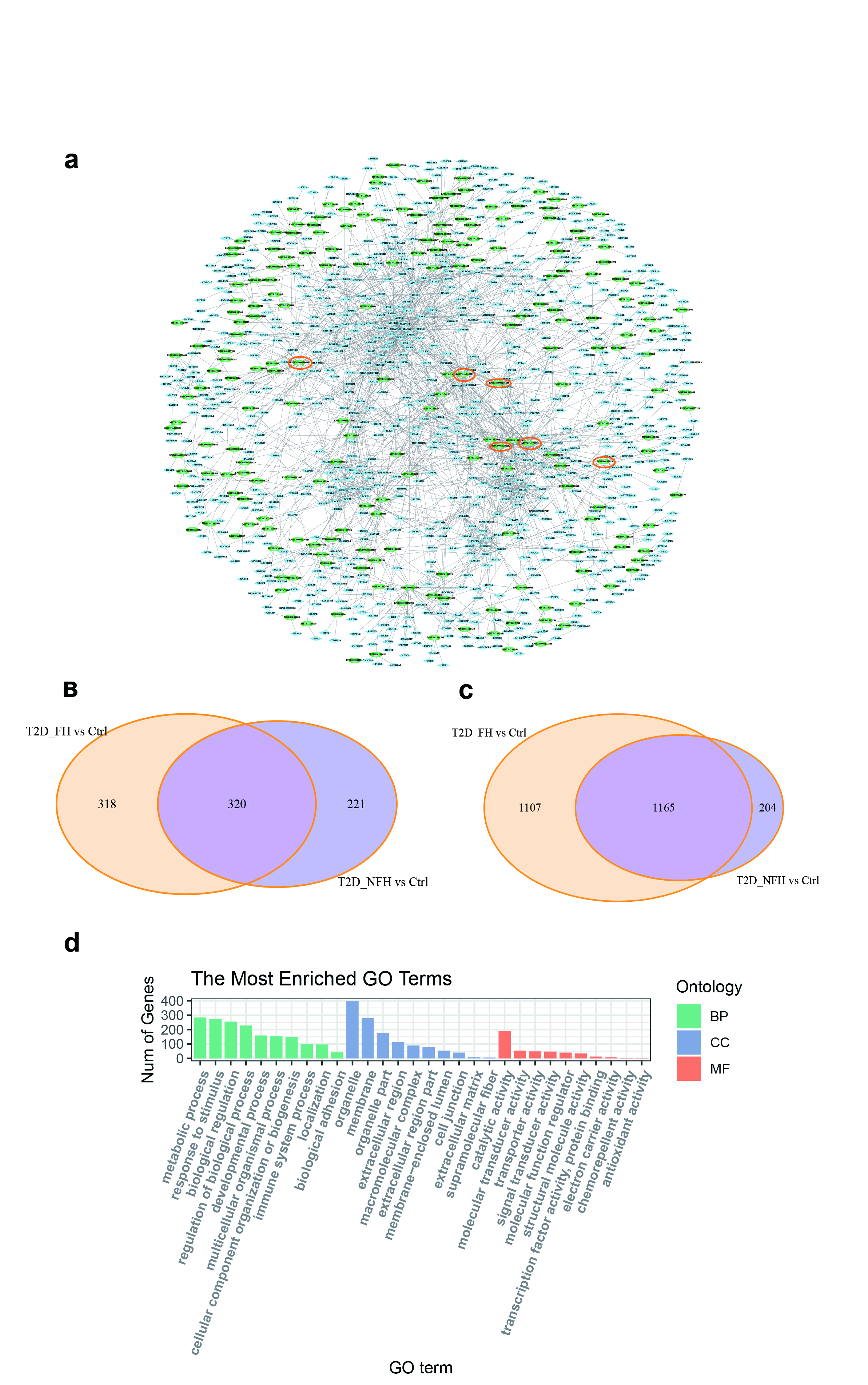

Supplement: Supplementary Figure 3 — (A) Co-expression networks of lncRNA-mRNA involved 1076 genes, consisted by 618 lncRNAs and 458 mRNAs. Venn plot for the differentially expressed lncRNAs (B) and mRNAs (C) between T2DM with- and without- family history samples. (D) Go analysis for the overlapped genes of T2DM with- and without family history samples. [file Image_3.tif]
