# Supplementary material for: Profile Screening of Differentially Expressed lncRNAs of Circulating Leukocytes in Type 2 Diabetes Patients and Differences From Type 1 Diabetes
Source: Front Endocrinol (Lausanne). 2022 Jan 10;12:690555. doi: 10.3389/fendo.2021.690555 (PMC8786112; doi:10.3389/fendo.2021.690555)
Supplement: Supplementary file 5 [file DataSheet_2.docx]

Supplemental Tables

Table S1 Clinical characteristics of included patients of type 2 diabetes in discovery cohorts (n=11) and in validation cohorts (n=56)

Table S2 The information of 21 candidate lncRNAs which were successfully established PCR condition and further detected in the validation cohorts, including the name, lncRNAs with significant difference in RNA-sequencing data or not, novel/known, up/down, biotype, position description, and primers used in validation by quantitative PCR.

| **No.** | **Name** | **Novel/**  **Known** | **Up/**  **Down** | **Biotype** | **With significant difference in RNA-seq or not** | **Forward primer** | **Reverse primer** |
| --- | --- | --- | --- | --- | --- | --- | --- |
| 1 | ENSG00000224515 | Known | up | antisense | no | ACCTCCAAAGCAGCTCAACC | AGCTCACTGAAAGCCCACAT |
| 2 | ENSG00000234389 | Known | up | sense_intronic | yes | ACAATGGGGAGGCATTGAGG | ACTTCCGACCCAGCAAACAA |
| 3 | ENSG00000246263 | Known | up | antisense | yes | TGTTTGGGGGCAGCTACTAA | GGATAACTGGGGCAGACTTGA |
| 4 | ENSG00000257764 | Known | up | antisense | yes | ACCAGCTGATGAAGGCATCT | TCTCAATTCAGGCAGTCTGTTTC |
| 5 | ENSG00000269902 | Known | down | lincRNA | yes | AGGTTTAACAGGTCTTACTCCCAA | TTCAGGAGGGGAAATTGAGCC |
| 6 | ENSG00000276649 | Known | up | antisense | yes | TCTGGGCAAGAAGAGCCATT | AGCAACTCTTGGGGCAGTTG |
| 7 | ENSG00000280832 | Known | up | antisense | yes | TGCGTCACTGTCACCAAACT | CCATGCCCTATTCTGTGCCA |
| 8 | MSTRG.122492 | Novel | up | linc | no | GCCCAAGGGAGGATATTCCG | ATATTTCCACCGTGTCCCCC |
| 9 | MSTRG.128697 | Novel | up | linc | no | TTATCAACTCCGGCGAGCCA | GTCCCCATTTTACAGGCAAGGA |
| 10 | MSTRG.128958 | Novel | up | antisense | yes | ACACAGTCAGGGGATGAGGA | TGTACCCCACAAGCTGCATT |
| 11 | MSTRG.149099 | Novel | up | linc | yes | TAGGCTACACATGGGTCAGG | TGAGTAGCTGAACAAAAGGCAG |
| 12 | MSTRG.159131 | Novel | down | linc | no | TTGTCTTCAGGGAAACGGCT | GGTTTTGGCAAATGCCCTCC |
| 13 | MSTRG.166799 | Novel | up | antisense | yes | CCCAGATTCCGCATAGCGTT | ACCTCTTCATCTGCAGCCAAT |
| 14 | MSTRG.169545 | Novel | up | linc | no | GGAGCAGACCTTCCAGTTGT | GAAGGGGATGGGCAAACTCA |
| 15 | MSTRG.172533 | Novel | up | linc | yes | CCCTGTCATTCACCAACGCT | CCCCTGTTCCTGTATTGCCC |
| 16 | MSTRG.23167 | Novel | down | linc | no | TGAGCCTGATCATGTTGTACCC | AGGCAGGGGTGGTTTTAGAC |
| 17 | MSTRG.63013 | Novel | up | linc | yes | AGGGCCAATACTCAGTCCCT | ATGTCCGGCACTTAGGGAGA |
| 18 | MSTRG.64433 | Novel | up | linc | yes | AATGGTCGCTTTAAGCCCCT | GAGGTGCTCTGACCACACAA |
| 19 | MSTRG.72098 | Novel | up | linc | no | GAAAATCGACTCCGGTGGGA | ACCTTCTTAGCGCAGCACAA |
| 20 | MSTRG.74858 | Novel | down | linc | no | CCTGGTTAGGGCTGGTTGAA | CCTTCCTTCCTCCACCGTTC |
| 21 | MSTRG.95088 | Novel | down | linc | yes | GACCCATGAGAGCCCAAAGT | CCTGCCTGTTACCTCAGAGAG |

Table S3 A total of 845 significantly differentially expressed lncRNAs (T2D-lncRNAs) obtained from adjusted data.

| **Gene** | **Biotype** | **Position** | **C_normalize** | **T2D_normalize** | **log2 Fold Change** | ***p*-val** | **FDR** |
| --- | --- | --- | --- | --- | --- | --- | --- |
| ENSG00000267257 | antisense | chr18:58535415-58538552:+ | 19.44 | 0.55 | -5.13 | 0.0000 | 0.0000 |
| ENSG00000267258 | sense_intronic | chr15:39586561-39587293:+ | 39.88 | 3.37 | -3.56 | 0.0000 | 0.0000 |
| MSTRG.103146 | antisense | chr20:17570371-17583003:+ | 598.79 | 214.23 | -1.48 | 0.0000 | 0.0000 |
| MSTRG.19495 | linc | chr10:27465297-27468217:+ | 0.65 | 12.42 | 4.26 | 0.0000 | 0.0001 |
| ENSG00000273338 | antisense | chr1:78004346-78004554:- | 104.62 | 15.05 | -2.80 | 0.0000 | 0.0001 |
| MSTRG.180057 | linc | chr9:41229380-41230042:+ | 0.83 | 13.65 | 4.04 | 0.0000 | 0.0001 |
| MSTRG.22781 | antisense | chr10:87864018-87864678:- | 3.10 | 26.47 | 3.10 | 0.0000 | 0.0001 |
| MSTRG.161229 | antisense | chr7:27102269-27107589:+ | 27.29 | 128.52 | 2.24 | 0.0000 | 0.0001 |
| MSTRG.180437 | linc | chr9:62798833-62801387:+ | 201.79 | 585.68 | 1.54 | 0.0000 | 0.0001 |
| MSTRG.180334 | linc | chr9:61465105-61467945:+ | 0.60 | 11.82 | 4.31 | 0.0000 | 0.0001 |
| MSTRG.182419 | linc | chr9:91193131-91198833:+ | 88.48 | 13.76 | -2.69 | 0.0000 | 0.0001 |
| MSTRG.106807 | antisense | chr21:15047465-15064494:- | 2940.94 | 1210.00 | -1.28 | 0.0000 | 0.0001 |
| MSTRG.195300 | linc | chrY:8002712-8007593:- | 54.62 | 0.59 | -6.53 | 0.0000 | 0.0001 |
| MSTRG.62902 | antisense | chr16:2785942-2791742:+ | 0.64 | 11.46 | 4.17 | 0.0000 | 0.0001 |
| MSTRG.125714 | antisense | chr3:196215032-196228373:- | 8.73 | 47.42 | 2.44 | 0.0000 | 0.0001 |
| MSTRG.10587 | antisense | chr1:156211503-156218677:- | 4.64 | 28.06 | 2.59 | 0.0000 | 0.0001 |
| MSTRG.80841 | linc | chr19:10959257-10960669:- | 1.05 | 12.84 | 3.61 | 0.0000 | 0.0001 |
| MSTRG.181523 | antisense | chr9:78695042-78853673:+ | 1.29 | 18.00 | 3.80 | 0.0000 | 0.0002 |
| ENSG00000260244 | sense_overlapping | chr4:155734448-155737062:+ | 281.53 | 76.87 | -1.87 | 0.0000 | 0.0002 |
| MSTRG.183281 | antisense | chr9:105707806-105713053:+ | 335.10 | 117.40 | -1.51 | 0.0000 | 0.0003 |
| MSTRG.93076 | linc | chr2:111187202-111187998:+ | 0.33 | 7.89 | 4.60 | 0.0000 | 0.0003 |
| MSTRG.116382 | linc | chr3:49355335-49356922:- | 309.53 | 97.42 | -1.67 | 0.0000 | 0.0003 |
| MSTRG.83970 | antisense | chr19:46013762-46019049:- | 21.16 | 80.62 | 1.93 | 0.0000 | 0.0003 |
| ENSG00000215533 | sense_overlapping | chr21:29193480-29288205:+ | 6.24 | 45.86 | 2.88 | 0.0000 | 0.0003 |
| MSTRG.180056 | linc | chr9:41229379-41230044:- | 5.75 | 47.92 | 3.06 | 0.0000 | 0.0003 |
| ENSG00000272967 | antisense | chr3:119579212-119579650:- | 7.14 | 0.33 | -4.46 | 0.0000 | 0.0004 |
| MSTRG.66083 | antisense | chr16:67998699-68001317:+ | 146.86 | 554.00 | 1.92 | 0.0000 | 0.0005 |
| ENSG00000253552 | antisense | chr7:27107777-27134302:+ | 3.25 | 21.23 | 2.71 | 0.0000 | 0.0005 |
| MSTRG.179846 | antisense | chr9:40059342-40106069:- | 1089.68 | 403.07 | -1.43 | 0.0000 | 0.0005 |
| ENSG00000276216 | lincRNA | chr1:145281116-145281462:+ | 104.74 | 29.43 | -1.83 | 0.0000 | 0.0006 |
| MSTRG.63013 | linc | chr16:3037243-3046951:- | 44.37 | 280.10 | 2.66 | 0.0000 | 0.0006 |
| MSTRG.16103 | linc | chr1:230552207-230556500:- | 3.76 | 22.43 | 2.58 | 0.0000 | 0.0006 |
| ENSG00000224307 | lincRNA | chr9:129282458-129285728:+ | 36.28 | 142.19 | 1.97 | 0.0000 | 0.0006 |
| MSTRG.149099 | linc | chr6:6668529-6668938:- | 0.16 | 6.55 | 5.32 | 0.0000 | 0.0006 |
| ENSG00000223387 | lincRNA | chr3:172560888-172595607:- | 2.02 | 17.17 | 3.09 | 0.0000 | 0.0006 |
| MSTRG.180491 | linc | chr9:63814492-63817650:- | 30.40 | 99.72 | 1.71 | 0.0000 | 0.0006 |
| MSTRG.150535 | antisense | chr6:32473789-32531328:+ | 40.05 | 6.96 | -2.53 | 0.0000 | 0.0006 |
| ENSG00000263590 | lincRNA | chr1:143790010-143797108:+ | 0.50 | 8.41 | 4.08 | 0.0000 | 0.0006 |
| MSTRG.5412 | linc | chr1:65034964-65038215:- | 48.67 | 265.28 | 2.45 | 0.0000 | 0.0007 |
| MSTRG.126704 | linc | chr4:6886155-6888188:+ | 9.20 | 49.66 | 2.43 | 0.0000 | 0.0007 |
| MSTRG.138541 | linc | chr5:39074483-39077107:+ | 37.48 | 166.43 | 2.15 | 0.0000 | 0.0008 |
| MSTRG.187859 | linc | chrX:20117156-20123616:- | 176.86 | 46.14 | -1.94 | 0.0000 | 0.0009 |
| MSTRG.90046 | linc | chr2:57614497-57646095:- | 415.05 | 172.88 | -1.26 | 0.0000 | 0.0009 |
| MSTRG.55835 | linc | chr14:98973454-99107682:- | 243.41 | 614.66 | 1.34 | 0.0000 | 0.0009 |
| MSTRG.60140 | linc | chr15:69971436-69975793:- | 1.83 | 16.27 | 3.15 | 0.0000 | 0.0009 |
| ENSG00000261218 | lincRNA | chr16:81738248-81767868:+ | 31.08 | 96.89 | 1.64 | 0.0000 | 0.0009 |
| MSTRG.122910 | antisense | chr3:151229627-151231683:- | 26.97 | 96.92 | 1.85 | 0.0000 | 0.0009 |
| ENSG00000261512 | lincRNA | chr16:46622861-46624451:- | 53.34 | 145.57 | 1.45 | 0.0000 | 0.0010 |
| ENSG00000248455 | lincRNA | chr5:17404019-17441694:+ | 1.95 | 13.93 | 2.84 | 0.0000 | 0.0010 |
| MSTRG.80933 | antisense | chr19:11315420-11320164:- | 57.30 | 366.94 | 2.68 | 0.0000 | 0.0010 |
| MSTRG.825 | antisense | chr1:9627245-9742715:+ | 1004.47 | 2320.36 | 1.21 | 0.0000 | 0.0010 |
| MSTRG.93732 | linc | chr2:120223519-120232968:- | 149.95 | 507.00 | 1.76 | 0.0000 | 0.0010 |
| MSTRG.31213 | antisense | chr11:73418913-73426723:+ | 220.58 | 583.18 | 1.40 | 0.0000 | 0.0010 |
| MSTRG.179077 | linc | chr9:27589073-27591369:- | 89.03 | 22.18 | -2.00 | 0.0000 | 0.0010 |
| MSTRG.64433 | linc | chr16:28076685-28097390:- | 192.26 | 828.74 | 2.11 | 0.0000 | 0.0010 |
| MSTRG.147880 | linc | chr5:178650106-178668781:- | 0.18 | 10.69 | 5.86 | 0.0000 | 0.0010 |
| MSTRG.159327 | antisense | chr7:6405349-6450822:+ | 98.43 | 362.70 | 1.88 | 0.0000 | 0.0010 |
| MSTRG.21857 | linc | chr10:72328686-72366452:- | 32.64 | 110.62 | 1.76 | 0.0000 | 0.0010 |
| MSTRG.185495 | linc | chr9:128668273-128670326:- | 1.22 | 12.32 | 3.34 | 0.0000 | 0.0011 |
| MSTRG.81088 | antisense | chr19:13172244-13218650:+ | 22.94 | 82.57 | 1.85 | 0.0000 | 0.0012 |
| MSTRG.144110 | linc | chr5:123721820-124204494:+ | 228.59 | 71.16 | -1.68 | 0.0000 | 0.0013 |
| MSTRG.21653 | antisense | chr10:63161563-63182887:+ | 10.82 | 43.55 | 2.01 | 0.0000 | 0.0013 |
| MSTRG.31454 | linc | chr11:72793790-72810827:+ | 10.35 | 40.69 | 1.98 | 0.0000 | 0.0013 |
| MSTRG.164855 | antisense | chr7:100156429-100158162:+ | 46.88 | 135.62 | 1.53 | 0.0000 | 0.0013 |
| MSTRG.30578 | antisense | chr11:65768007-65772614:- | 24.03 | 146.85 | 2.61 | 0.0000 | 0.0014 |
| ENSG00000269902 | lincRNA | chrX:45764772-45765299:- | 83.88 | 27.63 | -1.60 | 0.0000 | 0.0014 |
| MSTRG.125600 | antisense | chr3:193644046-193683209:- | 4176.06 | 1206.37 | -1.79 | 0.0000 | 0.0015 |
| MSTRG.39530 | linc | chr12:56197580-56199781:- | 6.69 | 28.99 | 2.12 | 0.0000 | 0.0015 |
| MSTRG.65777 | linc | chr16:56984544-56989411:- | 76.61 | 201.67 | 1.40 | 0.0000 | 0.0016 |
| MSTRG.183248 | linc | chr9:105025478-105029529:- | 5.13 | 37.71 | 2.88 | 0.0000 | 0.0017 |
| MSTRG.90359 | linc | chr2:65272261-65285448:+ | 454.73 | 1092.22 | 1.26 | 0.0000 | 0.0019 |
| MSTRG.38119 | antisense | chr12:32691425-32696218:+ | 79.54 | 26.63 | -1.58 | 0.0000 | 0.0019 |
| MSTRG.71335 | linc | chr17:42195078-42198266:- | 22.71 | 76.38 | 1.75 | 0.0000 | 0.0019 |
| MSTRG.111791 | linc | chr22:41419090-41428731:+ | 27.96 | 86.42 | 1.63 | 0.0000 | 0.0019 |
| ENSG00000279463 | sense_intronic | chr13:73844683-73845130:- | 119.50 | 43.46 | -1.46 | 0.0000 | 0.0019 |
| ENSG00000270022 | lincRNA | chr22:42615244-42615907:+ | 83.56 | 24.74 | -1.76 | 0.0000 | 0.0019 |
| MSTRG.110125 | linc | chr22:24531136-24539457:+ | 7.94 | 47.11 | 2.57 | 0.0000 | 0.0020 |
| MSTRG.30482 | antisense | chr11:65264541-65267962:- | 5.60 | 37.60 | 2.75 | 0.0000 | 0.0022 |
| ENSG00000235609 | lincRNA | chr21:14818843-15014430:- | 82.80 | 24.89 | -1.73 | 0.0000 | 0.0022 |
| MSTRG.46436 | antisense | chr13:31189540-31200026:- | 170.12 | 72.19 | -1.24 | 0.0000 | 0.0022 |
| MSTRG.147114 | linc | chr5:155744475-155752268:+ | 226.96 | 69.71 | -1.70 | 0.0000 | 0.0022 |
| MSTRG.171692 | antisense | chr8:61828165-61875588:- | 140.67 | 34.17 | -2.04 | 0.0000 | 0.0022 |
| ENSG00000256433 | lincRNA | chr12:6393905-6396148:+ | 10.22 | 38.30 | 1.91 | 0.0000 | 0.0022 |
| MSTRG.23568 | antisense | chr10:97761089-97766900:+ | 58.45 | 158.42 | 1.44 | 0.0000 | 0.0022 |
| ENSG00000267512 | antisense | chr19:13139617-13141147:- | 0.91 | 8.37 | 3.20 | 0.0001 | 0.0023 |
| ENSG00000224091 | antisense | chr11:5205041-5207308:+ | 6.82 | 0.65 | -3.39 | 0.0001 | 0.0024 |
| MSTRG.184237 | linc | chr9:112193628-112195696:- | 5.07 | 22.26 | 2.13 | 0.0001 | 0.0024 |
| MSTRG.128652 | linc | chr4:37877914-37888331:+ | 34.50 | 157.81 | 2.19 | 0.0001 | 0.0024 |
| ENSG00000237491 | lincRNA | chr1:778770-810060:+ | 198.80 | 80.80 | -1.30 | 0.0001 | 0.0024 |
| MSTRG.12801 | antisense | chr1:182586698-182599563:+ | 2.47 | 19.29 | 2.97 | 0.0001 | 0.0025 |
| MSTRG.154388 | antisense | chr6:106297287-106304946:- | 579.27 | 287.03 | -1.01 | 0.0001 | 0.0025 |
| MSTRG.183247 | linc | chr9:105023405-105063275:- | 25.05 | 171.68 | 2.78 | 0.0001 | 0.0025 |
| MSTRG.59262 | antisense | chr15:58446518-58451430:- | 3.61 | 22.54 | 2.64 | 0.0001 | 0.0025 |
| ENSG00000271761 | lincRNA | chr6:57902609-57903148:- | 6.48 | 0.29 | -4.46 | 0.0001 | 0.0025 |
| MSTRG.31440 | antisense | chr11:72759869-72814494:- | 630.21 | 2144.73 | 1.77 | 0.0001 | 0.0026 |
| MSTRG.3691 | antisense | chr1:45399609-45419514:- | 84.08 | 215.21 | 1.36 | 0.0001 | 0.0027 |
| ENSG00000261367 | antisense | chr16:30107675-30110541:+ | 0.18 | 6.83 | 5.21 | 0.0001 | 0.0027 |
| MSTRG.187195 | linc | chrX:1919802-1920351:- | 0.31 | 5.85 | 4.22 | 0.0001 | 0.0027 |
| MSTRG.44307 | antisense | chr12:120578330-120583128:+ | 79.71 | 230.67 | 1.53 | 0.0001 | 0.0027 |
| ENSG00000276136 | lincRNA | chr12:32000375-32001222:+ | 62.11 | 186.24 | 1.58 | 0.0001 | 0.0027 |
| ENSG00000270055 | sense_intronic | chr15:30487963-30490313:+ | 491.64 | 238.84 | -1.04 | 0.0001 | 0.0028 |
| MSTRG.116694 | linc | chr3:52251062-52260792:+ | 37.96 | 135.10 | 1.83 | 0.0001 | 0.0028 |
| ENSG00000231826 | lincRNA | chr2:43027853-43039547:- | 4.78 | 21.47 | 2.17 | 0.0001 | 0.0028 |
| MSTRG.2188 | linc | chr1:27044913-27064389:+ | 0.16 | 4.82 | 4.93 | 0.0001 | 0.0028 |
| MSTRG.122909 | antisense | chr3:151262011-151273554:- | 310.89 | 717.70 | 1.21 | 0.0001 | 0.0028 |
| MSTRG.116307 | antisense | chr3:48458614-48467681:- | 115.71 | 269.33 | 1.22 | 0.0001 | 0.0028 |
| MSTRG.99109 | antisense | chr2:201090426-201116833:- | 279.87 | 719.78 | 1.36 | 0.0001 | 0.0029 |
| MSTRG.165064 | antisense | chr7:103328437-103334335:- | 201.87 | 90.70 | -1.15 | 0.0001 | 0.0029 |
| MSTRG.33360 | linc | chr11:109859109-109860317:- | 20.70 | 3.40 | -2.61 | 0.0001 | 0.0031 |
| MSTRG.53949 | linc | chr14:68856109-68859581:- | 9.97 | 50.60 | 2.34 | 0.0001 | 0.0031 |
| MSTRG.38036 | linc | chr12:31995136-32037418:+ | 485.61 | 1706.05 | 1.81 | 0.0001 | 0.0032 |
| MSTRG.172268 | antisense | chr8:67077666-67080602:+ | 60.92 | 21.13 | -1.53 | 0.0001 | 0.0033 |
| ENSG00000254810 | lincRNA | chr11:76654169-76656712:- | 133.45 | 39.33 | -1.76 | 0.0001 | 0.0034 |
| MSTRG.139337 | linc | chr5:56453084-56457025:- | 62.35 | 163.34 | 1.39 | 0.0001 | 0.0034 |
| ENSG00000262202 | lincRNA | chr17:19112000-19112636:- | 42.10 | 10.20 | -2.05 | 0.0001 | 0.0035 |
| MSTRG.111888 | linc | chr22:42446987-42450777:+ | 3.15 | 15.99 | 2.34 | 0.0001 | 0.0035 |
| ENSG00000277825 | lincRNA | chr19:16352462-16353182:- | 0.33 | 5.50 | 4.07 | 0.0001 | 0.0035 |
| MSTRG.108623 | linc | chr21:42784049-42792158:+ | 5.38 | 35.11 | 2.71 | 0.0001 | 0.0036 |
| MSTRG.172438 | antisense | chr8:73014128-73019595:+ | 230.21 | 102.95 | -1.16 | 0.0001 | 0.0036 |
| MSTRG.154957 | linc | chr6:113050454-113084199:+ | 22.00 | 4.35 | -2.34 | 0.0001 | 0.0036 |
| MSTRG.2982 | antisense | chr1:38417066-38475152:- | 9.43 | 54.12 | 2.52 | 0.0001 | 0.0036 |
| ENSG00000272341 | lincRNA | chr6:16764346-16766883:+ | 104.64 | 40.86 | -1.36 | 0.0001 | 0.0036 |
| MSTRG.167609 | antisense | chr7:150450476-150451324:- | 4.54 | 19.76 | 2.12 | 0.0001 | 0.0036 |
| MSTRG.23200 | linc | chr10:91153418-91163210:- | 273.92 | 129.54 | -1.08 | 0.0001 | 0.0037 |
| ENSG00000251661 | antisense | chr11:318640-325631:+ | 67.47 | 161.58 | 1.26 | 0.0001 | 0.0037 |
| MSTRG.117056 | linc | chr3:57937488-57946101:+ | 23.45 | 69.60 | 1.57 | 0.0001 | 0.0039 |
| ENSG00000276704 | sense_intronic | chr13:97437268-97437630:+ | 54.50 | 19.30 | -1.50 | 0.0001 | 0.0039 |
| ENSG00000225963 | antisense | chr2:230121370-230174223:+ | 1.22 | 8.81 | 2.85 | 0.0001 | 0.0039 |
| MSTRG.20446 | linc | chr10:12270186-12271320:+ | 2.81 | 14.58 | 2.37 | 0.0001 | 0.0039 |
| MSTRG.43453 | antisense | chr12:107757251-107758229:- | 112.19 | 45.75 | -1.29 | 0.0001 | 0.0039 |
| MSTRG.59140 | antisense | chr15:55169618-55354124:- | 165.38 | 377.41 | 1.19 | 0.0001 | 0.0039 |
| ENSG00000255240 | antisense | chr11:58933643-59058659:- | 247.14 | 97.76 | -1.34 | 0.0001 | 0.0041 |
| MSTRG.76257 | linc | chr18:21637627-21648684:- | 47.99 | 165.39 | 1.79 | 0.0001 | 0.0041 |
| ENSG00000260401 | sense_overlapping | chr11:73238975-73242335:+ | 26.33 | 108.66 | 2.04 | 0.0001 | 0.0042 |
| MSTRG.20283 | antisense | chr10:43731087-43735478:- | 89.70 | 5.05 | -4.15 | 0.0002 | 0.0044 |
| MSTRG.145568 | linc | chr5:140578035-140629799:- | 222.37 | 803.13 | 1.85 | 0.0002 | 0.0044 |
| MSTRG.139451 | linc | chr5:57169734-57173724:- | 118.36 | 46.55 | -1.35 | 0.0002 | 0.0046 |
| MSTRG.134161 | antisense | chr4:146277703-146337828:+ | 29.79 | 6.63 | -2.17 | 0.0002 | 0.0046 |
| ENSG00000225342 | antisense | chr12:40186009-40224915:- | 110.19 | 312.53 | 1.50 | 0.0002 | 0.0046 |
| ENSG00000232891 | sense_intronic | chr6:150600834-150605850:+ | 49.29 | 15.08 | -1.71 | 0.0002 | 0.0046 |
| MSTRG.96702 | antisense | chr2:171928087-171934889:+ | 270.07 | 122.17 | -1.14 | 0.0002 | 0.0048 |
| ENSG00000254789 | lincRNA | chr11:15571819-15622403:- | 6.70 | 85.52 | 3.67 | 0.0002 | 0.0051 |
| MSTRG.20040 | linc | chr10:35581361-35587348:+ | 15.92 | 50.13 | 1.65 | 0.0002 | 0.0051 |
| MSTRG.173929 | linc | chr8:90198580-90221372:- | 35.66 | 4.97 | -2.84 | 0.0002 | 0.0051 |
| MSTRG.103519 | linc | chr20:23085665-23095514:+ | 3.81 | 16.67 | 2.13 | 0.0002 | 0.0051 |
| ENSG00000233214 | lincRNA | chr19:35424062-35424652:- | 3.93 | 20.40 | 2.38 | 0.0002 | 0.0051 |
| ENSG00000280832 | antisense | chr11:126340889-126355587:- | 59.06 | 182.35 | 1.63 | 0.0002 | 0.0051 |
| ENSG00000225889 | antisense | chr2:64143239-64252859:+ | 281.75 | 991.08 | 1.81 | 0.0002 | 0.0052 |
| MSTRG.5497 | antisense | chr1:67754737-67829642:+ | 98.28 | 27.38 | -1.84 | 0.0002 | 0.0052 |
| ENSG00000261416 | antisense | chr16:30183505-30184957:- | 27.13 | 74.02 | 1.45 | 0.0002 | 0.0052 |
| MSTRG.108059 | antisense | chr21:34517762-34538256:+ | 14.58 | 45.65 | 1.65 | 0.0002 | 0.0052 |
| MSTRG.47505 | antisense | chr13:47111320-47419215:- | 9.55 | 1.19 | -3.01 | 0.0002 | 0.0052 |
| MSTRG.187021 | antisense | chrX:10018875-10037317:+ | 25.96 | 126.62 | 2.29 | 0.0002 | 0.0053 |
| ENSG00000273989 | sense_intronic | chr12:28236227-28236828:+ | 60.88 | 21.01 | -1.53 | 0.0002 | 0.0055 |
| ENSG00000269246 | lincRNA | chr19:39341773-39341945:- | 7.26 | 34.03 | 2.23 | 0.0002 | 0.0055 |
| MSTRG.47689 | antisense | chr13:48480787-48485893:- | 50.32 | 129.00 | 1.36 | 0.0002 | 0.0055 |
| ENSG00000270640 | sense_intronic | chr2:28396815-28397110:+ | 159.91 | 59.42 | -1.43 | 0.0002 | 0.0056 |
| MSTRG.180662 | linc | chr9:65811285-65814847:- | 70.07 | 15.76 | -2.15 | 0.0002 | 0.0057 |
| ENSG00000248559 | antisense | chr5:134399495-134401921:+ | 41.98 | 106.86 | 1.35 | 0.0002 | 0.0057 |
| ENSG00000231485 | lincRNA | chr1:65066627-65067737:- | 5.30 | 20.72 | 1.97 | 0.0002 | 0.0057 |
| MSTRG.80397 | linc | chr19:6884012-6885654:+ | 0.32 | 5.16 | 4.02 | 0.0002 | 0.0058 |
| ENSG00000272501 | antisense | chr6:31195200-31198037:- | 250.17 | 520.54 | 1.06 | 0.0002 | 0.0058 |
| ENSG00000248727 | lincRNA | chr5:56457795-56481946:- | 33.18 | 91.35 | 1.46 | 0.0002 | 0.0058 |
| MSTRG.49388 | linc | chr13:88709424-88744650:+ | 14.94 | 1.60 | -3.22 | 0.0002 | 0.0058 |
| MSTRG.46819 | linc | chr13:37089912-37091146:- | 6.68 | 41.08 | 2.62 | 0.0002 | 0.0058 |
| ENSG00000235499 | lincRNA | chr2:73985132-73986343:+ | 20.07 | 56.69 | 1.50 | 0.0003 | 0.0059 |
| ENSG00000253394 | lincRNA | chr8:90221488-90569318:+ | 73.33 | 21.75 | -1.75 | 0.0003 | 0.0061 |
| MSTRG.182532 | linc | chr9:93044764-93049979:+ | 5.51 | 25.58 | 2.21 | 0.0003 | 0.0061 |
| ENSG00000278330 | antisense | chr18:77112602-77115726:+ | 22.50 | 5.87 | -1.94 | 0.0003 | 0.0062 |
| ENSG00000253174 | antisense | chr8:41540381-41545044:- | 4.40 | 0.28 | -3.97 | 0.0003 | 0.0062 |
| MSTRG.39819 | antisense | chr12:57466810-57471270:- | 101.90 | 372.16 | 1.87 | 0.0003 | 0.0063 |
| MSTRG.154953 | antisense | chr6:112910851-113065948:- | 31.43 | 7.84 | -2.00 | 0.0003 | 0.0063 |
| MSTRG.47717 | linc | chr13:49639058-49671747:+ | 181.00 | 568.69 | 1.65 | 0.0003 | 0.0063 |
| ENSG00000270069 | lincRNA | chrX:45745211-45770274:- | 420.25 | 133.73 | -1.65 | 0.0003 | 0.0063 |
| ENSG00000272506 | lincRNA | chr1:65003470-65004087:- | 11.17 | 37.45 | 1.75 | 0.0003 | 0.0065 |
| ENSG00000226091 | lincRNA | chr12:8295986-8396803:- | 152.92 | 343.69 | 1.17 | 0.0003 | 0.0065 |
| MSTRG.30000 | linc | chr11:47452518-47462678:- | 71.35 | 175.49 | 1.30 | 0.0003 | 0.0065 |
| ENSG00000235728 | lincRNA | chr7:36781008-36782789:+ | 11.79 | 1.76 | -2.75 | 0.0003 | 0.0066 |
| MSTRG.82703 | linc | chr19:35455075-35456852:+ | 2.26 | 33.10 | 3.87 | 0.0003 | 0.0068 |
| MSTRG.141046 | linc | chr5:70156735-70179224:- | 214.62 | 92.05 | -1.22 | 0.0003 | 0.0068 |
| MSTRG.121315 | linc | chr3:129080251-129082986:- | 0.34 | 4.71 | 3.78 | 0.0003 | 0.0069 |
| MSTRG.195803 | linc | chrY:20345605-20349603:+ | 0.32 | 4.80 | 3.89 | 0.0003 | 0.0071 |
| ENSG00000228063 | antisense | chr1:219086602-219173961:- | 97.83 | 31.30 | -1.64 | 0.0003 | 0.0072 |
| MSTRG.179970 | linc | chr9:41452326-41453266:+ | 45.03 | 14.12 | -1.67 | 0.0004 | 0.0073 |
| MSTRG.166799 | antisense | chr7:139951772-139984965:- | 1.55 | 22.77 | 3.88 | 0.0003 | 0.0073 |
| MSTRG.133801 | antisense | chr4:139033397-139042812:- | 106.40 | 334.13 | 1.65 | 0.0004 | 0.0075 |
| MSTRG.38692 | antisense | chr12:47742772-47747243:+ | 23.45 | 68.94 | 1.56 | 0.0004 | 0.0075 |
| MSTRG.90735 | linc | chr2:64267944-64277593:- | 217.30 | 794.31 | 1.87 | 0.0004 | 0.0075 |
| MSTRG.11371 | linc | chr1:161634393-161637895:- | 3.08 | 36.71 | 3.58 | 0.0004 | 0.0076 |
| MSTRG.82708 | antisense | chr19:35323892-35419611:- | 18.11 | 109.97 | 2.60 | 0.0004 | 0.0076 |
| MSTRG.154192 | linc | chr6:104338448-104688481:- | 20.67 | 5.79 | -1.84 | 0.0004 | 0.0076 |
| MSTRG.153103 | antisense | chr6:77148321-77369160:- | 105.16 | 44.18 | -1.25 | 0.0004 | 0.0076 |
| ENSG00000248773 | sense_intronic | chr3:140972744-140973255:+ | 47.73 | 17.53 | -1.44 | 0.0004 | 0.0076 |
| MSTRG.128662 | linc | chr4:38139377-38140379:+ | 20.79 | 60.77 | 1.55 | 0.0004 | 0.0076 |
| ENSG00000230470 | sense_intronic | chr1:184408337-184412360:+ | 38.08 | 13.60 | -1.49 | 0.0004 | 0.0076 |
| MSTRG.30136 | linc | chr11:62025111-62029411:+ | 266.29 | 90.83 | -1.55 | 0.0004 | 0.0076 |
| ENSG00000274767 | antisense | chr17:36183235-36196471:+ | 114.09 | 241.95 | 1.08 | 0.0004 | 0.0076 |
| ENSG00000238005 | lincRNA | chr1:234957342-234970062:- | 49.22 | 159.40 | 1.70 | 0.0004 | 0.0076 |
| ENSG00000237886 | antisense | chr9:136546212-136549893:+ | 10.58 | 34.53 | 1.71 | 0.0004 | 0.0076 |
| MSTRG.107964 | antisense | chr21:33323679-33325423:- | 26.23 | 73.89 | 1.49 | 0.0004 | 0.0076 |
| MSTRG.141026 | linc | chr5:81818225-81845739:+ | 3.97 | 27.45 | 2.79 | 0.0004 | 0.0076 |
| MSTRG.65145 | linc | chr16:50658948-50663258:- | 57.90 | 180.80 | 1.64 | 0.0004 | 0.0077 |
| ENSG00000230107 | lincRNA | chr22:42438023-42446195:+ | 2.30 | 11.32 | 2.30 | 0.0004 | 0.0081 |
| MSTRG.128957 | linc | chr4:40409396-40416959:- | 23.34 | 65.93 | 1.50 | 0.0004 | 0.0081 |
| MSTRG.1963 | linc | chr1:21210733-21217072:- | 27.78 | 103.79 | 1.90 | 0.0004 | 0.0081 |
| ENSG00000224184 | lincRNA | chr2:11848622-12578348:+ | 17.20 | 1.88 | -3.19 | 0.0004 | 0.0081 |
| ENSG00000281005 | lincRNA | chr16:3263743-3267567:+ | 258.31 | 529.67 | 1.04 | 0.0004 | 0.0082 |
| ENSG00000281162 | lincRNA | chr2:101962056-101987167:+ | 50.45 | 207.38 | 2.04 | 0.0004 | 0.0082 |
| MSTRG.12636 | linc | chr1:173926307-173930515:- | 64.04 | 147.85 | 1.21 | 0.0004 | 0.0082 |
| ENSG00000226334 | antisense | chr9:104927553-104928892:+ | 2.23 | 10.21 | 2.19 | 0.0004 | 0.0083 |
| MSTRG.69881 | antisense | chr17:28880539-28882965:+ | 1.74 | 9.59 | 2.46 | 0.0005 | 0.0083 |
| MSTRG.142970 | linc | chr5:109869957-109870453:+ | 3.62 | 15.92 | 2.14 | 0.0005 | 0.0085 |
| MSTRG.153609 | linc | chr6:89357830-89362898:- | 9.83 | 31.97 | 1.70 | 0.0005 | 0.0086 |
| MSTRG.110441 | linc | chr22:29779652-29781311:- | 0.48 | 5.06 | 3.39 | 0.0005 | 0.0086 |
| MSTRG.10585 | linc | chr1:156180278-156191023:+ | 178.27 | 770.33 | 2.11 | 0.0005 | 0.0088 |
| ENSG00000269570 | antisense | chr11:58611119-58612642:- | 0.32 | 4.19 | 3.70 | 0.0005 | 0.0089 |
| MSTRG.21186 | linc | chr10:46093349-46095534:- | 6.25 | 23.74 | 1.92 | 0.0005 | 0.0089 |
| ENSG00000245149 | lincRNA | chr8:124462485-124474576:- | 166.56 | 77.44 | -1.10 | 0.0005 | 0.0090 |
| ENSG00000225798 | antisense | chr15:58889967-58894082:+ | 0.43 | 5.01 | 3.53 | 0.0005 | 0.0091 |
| MSTRG.166966 | linc | chr7:140924883-140927109:+ | 89.21 | 24.95 | -1.84 | 0.0005 | 0.0091 |
| MSTRG.83359 | antisense | chr19:40833499-40885937:+ | 80.45 | 261.60 | 1.70 | 0.0005 | 0.0092 |
| MSTRG.189033 | antisense | chrX:44285879-44290261:- | 0.94 | 6.87 | 2.87 | 0.0005 | 0.0092 |
| MSTRG.24156 | antisense | chr10:104036202-104039518:+ | 14.54 | 45.14 | 1.63 | 0.0005 | 0.0093 |
| ENSG00000234389 | sense_intronic | chr2:102438713-102440475:+ | 182.52 | 631.45 | 1.79 | 0.0005 | 0.0094 |
| MSTRG.352 | antisense | chr1:631800-632316:- | 3.24 | 0.07 | -5.47 | 0.0005 | 0.0094 |
| MSTRG.189465 | linc | chrX:48665501-48667155:- | 0.16 | 3.58 | 4.45 | 0.0006 | 0.0094 |
| MSTRG.16534 | linc | chr1:235654423-235660031:- | 57.44 | 183.33 | 1.67 | 0.0006 | 0.0094 |
| ENSG00000246263 | antisense | chr8:102239394-102253333:+ | 224.30 | 458.50 | 1.03 | 0.0006 | 0.0094 |
| MSTRG.73803 | linc | chr17:74487168-74517616:+ | 81.26 | 207.66 | 1.35 | 0.0006 | 0.0095 |
| ENSG00000257809 | antisense | chr12:56150796-56158220:- | 3.14 | 13.85 | 2.14 | 0.0006 | 0.0096 |
| ENSG00000232912 | antisense | chr1:8424645-8434838:+ | 30.61 | 78.31 | 1.36 | 0.0006 | 0.0096 |
| ENSG00000255026 | antisense | chr11:287305-288987:+ | 121.57 | 256.69 | 1.08 | 0.0006 | 0.0097 |
| MSTRG.92719 | linc | chr2:105635381-105713303:- | 184.35 | 70.08 | -1.40 | 0.0006 | 0.0097 |
| ENSG00000264188 | sense_intronic | chr18:21661787-21662395:- | 3.85 | 15.66 | 2.02 | 0.0006 | 0.0099 |
| ENSG00000271327 | lincRNA | chr12:89367807-89369301:+ | 4.85 | 18.44 | 1.93 | 0.0006 | 0.0100 |
| MSTRG.248 | antisense | chr1:2545568-2547276:- | 15.34 | 58.38 | 1.93 | 0.0006 | 0.0104 |
| ENSG00000245694 | lincRNA | chr16:54845189-54929189:- | 8.66 | 1.86 | -2.22 | 0.0007 | 0.0105 |
| MSTRG.43950 | antisense | chr12:112267365-112269434:- | 7.88 | 26.01 | 1.72 | 0.0006 | 0.0105 |
| ENSG00000238164 | antisense | chr1:2549920-2557031:- | 134.80 | 279.24 | 1.05 | 0.0006 | 0.0105 |
| MSTRG.110112 | linc | chr22:24529561-24532569:+ | 5.80 | 24.68 | 2.09 | 0.0007 | 0.0105 |
| MSTRG.12579 | linc | chr1:180152756-180154475:+ | 1.12 | 11.19 | 3.32 | 0.0007 | 0.0105 |
| ENSG00000261043 | lincRNA | chr15:75759501-75762405:- | 1.12 | 30.14 | 4.75 | 0.0007 | 0.0105 |
| ENSG00000251442 | lincRNA | chr4:78645903-78682699:+ | 27.75 | 91.04 | 1.71 | 0.0007 | 0.0106 |
| ENSG00000203497 | antisense | chr10:110869868-110872233:- | 92.31 | 187.49 | 1.02 | 0.0007 | 0.0106 |
| ENSG00000275371 | antisense | chr16:30110895-30111955:+ | 3.35 | 19.21 | 2.52 | 0.0007 | 0.0106 |
| ENSG00000226239 | antisense | chr20:32027753-32031575:- | 0.16 | 3.60 | 4.51 | 0.0007 | 0.0106 |
| MSTRG.120650 | linc | chr3:122730938-122733204:+ | 8.86 | 27.32 | 1.62 | 0.0007 | 0.0106 |
| ENSG00000224152 | antisense | chr2:159615296-159617082:+ | 84.07 | 259.81 | 1.63 | 0.0007 | 0.0106 |
| MSTRG.13786 | antisense | chr1:200345350-200347125:- | 1.98 | 13.36 | 2.75 | 0.0007 | 0.0106 |
| MSTRG.182748 | antisense | chr9:96402030-96414736:- | 60.94 | 22.79 | -1.42 | 0.0007 | 0.0106 |
| MSTRG.129614 | antisense | chr4:56412310-56415249:- | 51.86 | 20.21 | -1.36 | 0.0007 | 0.0106 |
| MSTRG.181983 | linc | chr9:85533984-85540704:- | 25.67 | 67.34 | 1.39 | 0.0007 | 0.0107 |
| ENSG00000177757 | lincRNA | chr1:817371-819837:+ | 3.17 | 0.20 | -4.00 | 0.0007 | 0.0107 |
| ENSG00000274536 | antisense | chrX:66015461-66020422:+ | 979.86 | 2487.62 | 1.34 | 0.0007 | 0.0107 |
| MSTRG.89332 | linc | chr2:49601869-49784790:+ | 98.73 | 36.29 | -1.44 | 0.0007 | 0.0108 |
| MSTRG.174310 | linc | chr8:100907978-100911871:+ | 0.16 | 3.68 | 4.49 | 0.0007 | 0.0110 |
| MSTRG.137019 | antisense | chr5:10492129-10498784:+ | 17.79 | 99.85 | 2.49 | 0.0007 | 0.0110 |
| MSTRG.99519 | linc | chr2:207807527-207812837:- | 3.71 | 23.47 | 2.66 | 0.0007 | 0.0111 |
| MSTRG.90854 | linc | chr2:71081243-71086367:+ | 149.06 | 303.95 | 1.03 | 0.0007 | 0.0111 |
| MSTRG.157175 | linc | chr6:141831065-142030439:- | 37.41 | 10.26 | -1.87 | 0.0008 | 0.0112 |
| ENSG00000273149 | antisense | chr13:45340039-45341183:+ | 26.61 | 68.70 | 1.37 | 0.0008 | 0.0112 |
| MSTRG.65923 | antisense | chr16:67199589-67200633:- | 5.06 | 20.41 | 2.01 | 0.0008 | 0.0112 |
| MSTRG.36344 | linc | chr12:8323149-8453159:- | 90.97 | 203.28 | 1.16 | 0.0008 | 0.0116 |
| MSTRG.15600 | linc | chr1:226042936-226059382:- | 28.37 | 70.63 | 1.32 | 0.0008 | 0.0116 |
| ENSG00000255197 | antisense | chr11:47383148-47409190:- | 292.04 | 739.99 | 1.34 | 0.0008 | 0.0117 |
| MSTRG.180364 | linc | chr9:63037907-63043047:- | 65.63 | 18.56 | -1.82 | 0.0008 | 0.0117 |
| MSTRG.138918 | linc | chr5:50129000-50145864:- | 149.45 | 72.18 | -1.05 | 0.0008 | 0.0117 |
| MSTRG.89063 | linc | chr2:43135241-43145678:- | 25.39 | 64.31 | 1.34 | 0.0008 | 0.0117 |
| ENSG00000259548 | lincRNA | chr15:79832466-79833554:- | 9.40 | 40.74 | 2.12 | 0.0008 | 0.0117 |
| MSTRG.154253 | linc | chr6:105453557-105480939:+ | 213.84 | 71.07 | -1.59 | 0.0008 | 0.0117 |
| ENSG00000259786 | lincRNA | chr5:29143503-29217115:+ | 0.16 | 3.55 | 4.49 | 0.0008 | 0.0117 |
| ENSG00000276241 | antisense | chr17:36116177-36177510:+ | 40.84 | 15.16 | -1.43 | 0.0008 | 0.0117 |
| ENSG00000274422 | lincRNA | chr22:22283928-22287220:- | 139.71 | 62.69 | -1.16 | 0.0008 | 0.0117 |
| MSTRG.43515 | linc | chr12:108607214-108612641:- | 60.12 | 157.85 | 1.39 | 0.0008 | 0.0117 |
| ENSG00000250734 | antisense | chr1:151612038-151613363:- | 0.58 | 5.26 | 3.19 | 0.0008 | 0.0117 |
| ENSG00000232184 | sense_intronic | chr1:243702857-243740821:- | 61.63 | 25.42 | -1.28 | 0.0009 | 0.0118 |
| MSTRG.11326 | linc | chr1:161972044-161977537:+ | 18.06 | 49.45 | 1.45 | 0.0009 | 0.0118 |
| ENSG00000250616 | antisense | chr16:30096430-30104116:+ | 40.12 | 169.07 | 2.08 | 0.0009 | 0.0118 |
| MSTRG.192676 | linc | chrX:107636256-107677157:- | 219.46 | 457.05 | 1.06 | 0.0008 | 0.0118 |
| MSTRG.179483 | antisense | chr9:35015555-35020180:- | 5.14 | 18.68 | 1.86 | 0.0009 | 0.0118 |
| ENSG00000269919 | sense_intronic | chr6:106100140-106100593:+ | 111.68 | 50.15 | -1.15 | 0.0008 | 0.0118 |
| ENSG00000260019 | lincRNA | chr17:27929548-27974223:- | 7.66 | 1.48 | -2.38 | 0.0009 | 0.0120 |
| MSTRG.190993 | antisense | chrX:71349542-71352520:- | 13.19 | 41.75 | 1.66 | 0.0009 | 0.0122 |
| ENSG00000231528 | lincRNA | chr9:113113073-113119928:+ | 0.91 | 6.19 | 2.77 | 0.0009 | 0.0125 |
| MSTRG.101764 | linc | chr2:239503538-239529710:+ | 9.83 | 44.88 | 2.19 | 0.0009 | 0.0125 |
| MSTRG.179264 | linc | chr9:33374436-33376085:+ | 2.08 | 9.58 | 2.20 | 0.0010 | 0.0131 |
| MSTRG.111430 | antisense | chr22:37741031-37745984:- | 26.44 | 91.86 | 1.80 | 0.0010 | 0.0131 |
| MSTRG.119201 | linc | chr3:105280540-105287680:- | 17.27 | 53.85 | 1.64 | 0.0010 | 0.0131 |
| MSTRG.101996 | linc | chr2:241860015-241864616:+ | 17.82 | 4.74 | -1.91 | 0.0010 | 0.0131 |
| MSTRG.138569 | antisense | chr5:39197828-39212577:+ | 21.10 | 53.82 | 1.35 | 0.0010 | 0.0132 |
| ENSG00000243179 | lincRNA | chr2:113979909-113983258:+ | 16.43 | 46.27 | 1.49 | 0.0010 | 0.0132 |
| MSTRG.131944 | antisense | chr4:102007151-102018762:+ | 2216.58 | 851.16 | -1.38 | 0.0010 | 0.0133 |
| MSTRG.154591 | linc | chr6:108034922-108041033:- | 57.87 | 132.37 | 1.19 | 0.0010 | 0.0133 |
| ENSG00000270959 | antisense | chr3:188151206-188154057:- | 28.91 | 70.18 | 1.28 | 0.0010 | 0.0133 |
| ENSG00000232680 | lincRNA | chr19:35432957-35434642:- | 14.86 | 58.32 | 1.97 | 0.0010 | 0.0134 |
| MSTRG.80994 | antisense | chr19:12920127-12921809:- | 2.49 | 14.34 | 2.53 | 0.0010 | 0.0134 |
| ENSG00000265206 | antisense | chr17:58330884-58332508:- | 275.94 | 676.01 | 1.29 | 0.0010 | 0.0134 |
| MSTRG.84405 | antisense | chr19:49551093-49565172:+ | 39.92 | 101.57 | 1.35 | 0.0010 | 0.0134 |
| MSTRG.81376 | linc | chr19:16194658-16195478:+ | 1.07 | 6.93 | 2.70 | 0.0010 | 0.0135 |
| ENSG00000274737 | sense_intronic | chr12:47817451-47817966:- | 16.36 | 44.67 | 1.45 | 0.0010 | 0.0135 |
| MSTRG.151070 | antisense | chr6:36240423-36248360:- | 51.53 | 194.53 | 1.92 | 0.0011 | 0.0136 |
| ENSG00000255028 | antisense | chr11:109355085-109583907:- | 33.36 | 7.78 | -2.10 | 0.0011 | 0.0136 |
| MSTRG.80171 | antisense | chr19:4643117-4651481:+ | 3.79 | 15.81 | 2.06 | 0.0011 | 0.0136 |
| MSTRG.86979 | linc | chr2:20402140-20425143:- | 92.59 | 267.39 | 1.53 | 0.0011 | 0.0136 |
| MSTRG.151060 | antisense | chr6:35977706-35984240:+ | 0.79 | 5.84 | 2.88 | 0.0011 | 0.0138 |
| MSTRG.46317 | antisense | chr13:28138536-28139247:- | 16.28 | 45.41 | 1.48 | 0.0011 | 0.0138 |
| ENSG00000235531 | antisense | chr8:71828167-72118393:+ | 137.73 | 67.91 | -1.02 | 0.0011 | 0.0138 |
| ENSG00000269318 | antisense | chr19:4356637-4358448:- | 11.16 | 32.94 | 1.56 | 0.0011 | 0.0138 |
| MSTRG.67209 | linc | chr16:85025712-85027615:- | 6.27 | 19.83 | 1.66 | 0.0011 | 0.0138 |
| MSTRG.163135 | antisense | chr7:66383336-66386386:- | 23.20 | 58.97 | 1.35 | 0.0011 | 0.0139 |
| ENSG00000274173 | lincRNA | chr20:24931840-24932983:+ | 19.29 | 66.41 | 1.78 | 0.0011 | 0.0139 |
| MSTRG.165041 | linc | chr7:100281076-100282485:- | 1.69 | 9.30 | 2.46 | 0.0011 | 0.0139 |
| ENSG00000223653 | antisense | chr1:85276715-85448124:+ | 27.13 | 9.07 | -1.58 | 0.0011 | 0.0139 |
| ENSG00000280213 | antisense | chr20:63953384-63956985:+ | 24.25 | 63.50 | 1.39 | 0.0011 | 0.0139 |
| MSTRG.40287 | antisense | chr12:64699215-64741058:+ | 328.52 | 1014.06 | 1.63 | 0.0011 | 0.0139 |
| MSTRG.95087 | linc | chr2:144660953-144665439:+ | 94.34 | 24.99 | -1.92 | 0.0011 | 0.0139 |
| MSTRG.69730 | linc | chr17:27375415-27379169:- | 6.19 | 0.82 | -2.91 | 0.0011 | 0.0139 |
| MSTRG.80387 | linc | chr19:6858008-6881844:+ | 42.99 | 102.93 | 1.26 | 0.0011 | 0.0140 |
| MSTRG.80769 | linc | chr19:10347759-10348204:- | 0.75 | 5.60 | 2.90 | 0.0012 | 0.0141 |
| ENSG00000259376 | antisense | chr15:101043716-101049456:- | 2.83 | 11.96 | 2.08 | 0.0012 | 0.0141 |
| MSTRG.110255 | linc | chr22:26632025-26632729:- | 0.30 | 4.02 | 3.74 | 0.0012 | 0.0142 |
| ENSG00000273381 | lincRNA | chr9:91206175-91210299:- | 23.45 | 7.84 | -1.58 | 0.0012 | 0.0142 |
| MSTRG.103212 | linc | chr20:19207395-19212208:- | 43.68 | 13.22 | -1.72 | 0.0012 | 0.0146 |
| MSTRG.179556 | linc | chr9:36164910-36167780:+ | 45.05 | 164.97 | 1.87 | 0.0012 | 0.0147 |
| ENSG00000277214 | lincRNA | chr16:79603572-79604177:+ | 10.43 | 2.64 | -1.98 | 0.0012 | 0.0148 |
| MSTRG.23757 | linc | chr10:98361338-98368642:+ | 4.24 | 0.40 | -3.41 | 0.0013 | 0.0148 |
| ENSG00000269887 | sense_intronic | chr1:164680085-164680799:+ | 23.78 | 7.19 | -1.73 | 0.0013 | 0.0148 |
| MSTRG.45477 | linc | chr13:19800391-19807113:- | 5.07 | 18.81 | 1.89 | 0.0013 | 0.0148 |
| MSTRG.134041 | antisense | chr4:143654875-143693497:+ | 56.42 | 20.05 | -1.49 | 0.0013 | 0.0149 |
| MSTRG.110578 | linc | chr22:27835733-27836525:- | 1.49 | 8.25 | 2.47 | 0.0013 | 0.0149 |
| MSTRG.35821 | antisense | chr12:1796345-1797664:+ | 21.08 | 55.51 | 1.40 | 0.0013 | 0.0149 |
| MSTRG.81368 | antisense | chr19:16135156-16138551:+ | 29.76 | 71.45 | 1.26 | 0.0013 | 0.0149 |
| MSTRG.22946 | linc | chr10:89027822-89033111:+ | 58.97 | 132.43 | 1.17 | 0.0013 | 0.0149 |
| ENSG00000276649 | antisense | chr20:1888128-1894374:- | 96.63 | 329.06 | 1.77 | 0.0013 | 0.0150 |
| MSTRG.73962 | linc | chr17:67059965-67065759:- | 14.33 | 44.83 | 1.65 | 0.0013 | 0.0150 |
| MSTRG.26081 | linc | chr10:133259524-133260533:+ | 13.59 | 3.34 | -2.02 | 0.0013 | 0.0151 |
| MSTRG.172533 | linc | chr8:74032173-74043417:+ | 163.81 | 649.45 | 1.99 | 0.0013 | 0.0151 |
| ENSG00000279727 | lincRNA | chr3:36819276-36822498:- | 103.38 | 211.68 | 1.03 | 0.0013 | 0.0151 |
| ENSG00000261804 | lincRNA | chr16:53373493-53384745:+ | 75.72 | 239.44 | 1.66 | 0.0013 | 0.0152 |
| ENSG00000272477 | sense_intronic | chr3:18408680-18409635:- | 157.23 | 72.07 | -1.13 | 0.0013 | 0.0153 |
| ENSG00000276573 | sense_intronic | chr13:97435946-97436168:+ | 57.32 | 24.71 | -1.21 | 0.0014 | 0.0153 |
| MSTRG.8711 | antisense | chr1:110572094-110618959:+ | 33.42 | 9.69 | -1.79 | 0.0014 | 0.0155 |
| ENSG00000260912 | sense_overlapping | chr9:19453209-19455173:+ | 110.08 | 46.93 | -1.23 | 0.0014 | 0.0155 |
| ENSG00000273599 | antisense | chr10:124996064-125001491:+ | 24.79 | 60.47 | 1.29 | 0.0014 | 0.0156 |
| MSTRG.118864 | antisense | chr3:71584328-71644483:- | 1825.22 | 4449.87 | 1.29 | 0.0014 | 0.0157 |
| MSTRG.13303 | linc | chr1:192106198-192157884:+ | 1013.83 | 361.36 | -1.49 | 0.0014 | 0.0157 |
| MSTRG.172596 | linc | chr8:73878959-73882396:+ | 43.35 | 13.79 | -1.65 | 0.0014 | 0.0158 |
| ENSG00000225313 | antisense | chr1:33307348-33349245:+ | 2.24 | 10.21 | 2.19 | 0.0014 | 0.0159 |
| MSTRG.12928 | linc | chr1:185386731-185389229:- | 0.34 | 3.70 | 3.43 | 0.0015 | 0.0163 |
| MSTRG.119213 | linc | chr3:104337115-104526897:- | 68.66 | 27.37 | -1.33 | 0.0015 | 0.0163 |
| MSTRG.161766 | linc | chr7:30199265-30201979:+ | 63.10 | 135.69 | 1.10 | 0.0015 | 0.0164 |
| MSTRG.56358 | antisense | chr14:102368357-102394888:- | 17.37 | 70.40 | 2.02 | 0.0015 | 0.0164 |
| ENSG00000214407 | lincRNA | chr3:101940859-101997926:+ | 14.36 | 4.12 | -1.80 | 0.0015 | 0.0167 |
| MSTRG.3573 | linc | chr1:44200585-44206663:- | 33.05 | 8.70 | -1.93 | 0.0016 | 0.0168 |
| MSTRG.50234 | linc | chr13:111037132-111049278:+ | 4.80 | 0.46 | -3.37 | 0.0016 | 0.0168 |
| MSTRG.154361 | linc | chr6:105751245-105758200:- | 0.85 | 15.59 | 4.19 | 0.0016 | 0.0168 |
| MSTRG.83071 | antisense | chr19:38734269-38735428:+ | 0.42 | 4.43 | 3.41 | 0.0016 | 0.0168 |
| ENSG00000232721 | lincRNA | chr1:143736066-143739506:+ | 0.14 | 3.00 | 4.41 | 0.0015 | 0.0168 |
| MSTRG.116308 | linc | chr3:48513740-48516990:- | 29.32 | 110.27 | 1.91 | 0.0016 | 0.0169 |
| MSTRG.193308 | linc | chrX:118724910-118727398:- | 4.28 | 14.63 | 1.77 | 0.0016 | 0.0169 |
| ENSG00000261114 | sense_intronic | chr16:56941028-56941726:+ | 46.87 | 19.70 | -1.25 | 0.0016 | 0.0169 |
| MSTRG.30606 | antisense | chr11:67278449-67284497:- | 5.42 | 19.65 | 1.86 | 0.0016 | 0.0170 |
| MSTRG.1605 | antisense | chr1:19331623-19339722:+ | 9.33 | 26.41 | 1.50 | 0.0016 | 0.0170 |
| MSTRG.147689 | antisense | chr5:176872188-176880564:- | 159.52 | 504.27 | 1.66 | 0.0016 | 0.0170 |
| ENSG00000228215 | lincRNA | chr1:191875495-192011260:+ | 26.34 | 8.29 | -1.67 | 0.0016 | 0.0170 |
| MSTRG.53759 | linc | chr14:63765472-63786874:+ | 34.14 | 285.83 | 3.07 | 0.0016 | 0.0170 |
| MSTRG.127451 | linc | chr4:12123976-12125545:+ | 0.16 | 3.25 | 4.36 | 0.0016 | 0.0170 |
| ENSG00000230084 | antisense | chr3:42601963-42654388:- | 8.38 | 25.03 | 1.58 | 0.0016 | 0.0171 |
| MSTRG.130693 | linc | chr4:78727269-78754495:+ | 104.87 | 440.26 | 2.07 | 0.0016 | 0.0171 |
| MSTRG.68721 | linc | chr17:8213288-8218722:- | 10.04 | 28.84 | 1.52 | 0.0016 | 0.0171 |
| ENSG00000273402 | antisense | chr8:237045-237669:+ | 30.64 | 10.88 | -1.49 | 0.0016 | 0.0171 |
| MSTRG.39108 | linc | chr12:52016053-52016438:+ | 17.88 | 3.90 | -2.20 | 0.0016 | 0.0171 |
| MSTRG.151606 | antisense | chr6:43043514-43052909:+ | 2.29 | 10.05 | 2.14 | 0.0017 | 0.0174 |
| MSTRG.24421 | linc | chr10:110375366-110376179:- | 2.62 | 0.07 | -5.30 | 0.0017 | 0.0176 |
| MSTRG.128667 | antisense | chr4:38802984-38805618:+ | 0.60 | 5.77 | 3.27 | 0.0017 | 0.0176 |
| MSTRG.11365 | antisense | chr1:161512561-161525557:- | 3.20 | 23.39 | 2.87 | 0.0017 | 0.0177 |
| MSTRG.145922 | antisense | chr5:149203798-149205108:+ | 18.16 | 5.44 | -1.74 | 0.0017 | 0.0177 |
| MSTRG.81616 | antisense | chr19:18384111-18386466:- | 14.28 | 63.15 | 2.15 | 0.0017 | 0.0177 |
| ENSG00000224707 | sense_intronic | chr6:20437821-20440178:+ | 53.01 | 116.46 | 1.14 | 0.0017 | 0.0177 |
| MSTRG.60283 | antisense | chr15:64506449-64592774:+ | 0.60 | 4.70 | 2.96 | 0.0018 | 0.0177 |
| MSTRG.185097 | antisense | chr9:124647452-124703477:- | 27.06 | 92.95 | 1.78 | 0.0018 | 0.0177 |
| MSTRG.130692 | antisense | chr4:78625294-78643596:+ | 7.81 | 54.24 | 2.80 | 0.0017 | 0.0177 |
| ENSG00000275582 | antisense | chr20:3921279-3923400:- | 1.17 | 7.35 | 2.65 | 0.0017 | 0.0177 |
| MSTRG.26186 | antisense | chr11:282817-289087:- | 95.35 | 284.69 | 1.58 | 0.0018 | 0.0180 |
| MSTRG.61397 | antisense | chr15:83093855-83109055:- | 80.48 | 234.93 | 1.55 | 0.0018 | 0.0182 |
| MSTRG.106237 | antisense | chr20:60081563-60087668:+ | 25.71 | 62.43 | 1.28 | 0.0018 | 0.0182 |
| MSTRG.133008 | linc | chr4:119635696-119657423:- | 160.65 | 43.53 | -1.88 | 0.0018 | 0.0183 |
| MSTRG.39822 | antisense | chr12:57480489-57486959:+ | 383.41 | 795.32 | 1.05 | 0.0018 | 0.0183 |
| MSTRG.38251 | antisense | chr12:40191124-40210961:- | 53.79 | 230.63 | 2.10 | 0.0019 | 0.0184 |
| MSTRG.52953 | antisense | chr14:54681748-54729341:- | 1420.03 | 398.15 | -1.83 | 0.0019 | 0.0185 |
| ENSG00000272933 | lincRNA | chr10:102642792-102644140:- | 8.85 | 30.12 | 1.77 | 0.0019 | 0.0185 |
| ENSG00000228382 | sense_intronic | chr1:226656640-226675067:- | 72.78 | 149.06 | 1.03 | 0.0019 | 0.0185 |
| ENSG00000273565 | lincRNA | chr14:75176929-75177418:+ | 24.61 | 8.16 | -1.59 | 0.0019 | 0.0185 |
| MSTRG.125728 | linc | chr3:196443949-196458686:- | 16.28 | 43.75 | 1.43 | 0.0019 | 0.0186 |
| MSTRG.24582 | linc | chr10:111114161-111481889:+ | 176.23 | 70.87 | -1.31 | 0.0019 | 0.0187 |
| MSTRG.141988 | antisense | chr5:95790546-95792781:- | 11.11 | 31.42 | 1.50 | 0.0019 | 0.0187 |
| ENSG00000267765 | antisense | chr17:42683187-42699466:- | 4.73 | 16.07 | 1.76 | 0.0019 | 0.0187 |
| MSTRG.54498 | linc | chr14:75298906-75303521:+ | 94.28 | 37.54 | -1.33 | 0.0020 | 0.0188 |
| MSTRG.28733 | linc | chr11:33893889-33916520:+ | 108.65 | 44.78 | -1.28 | 0.0020 | 0.0189 |
| ENSG00000257433 | antisense | chr12:47706085-47742294:+ | 50.75 | 138.08 | 1.44 | 0.0020 | 0.0189 |
| ENSG00000254165 | antisense | chr8:42537529-42538304:- | 87.13 | 41.04 | -1.09 | 0.0019 | 0.0189 |
| MSTRG.128382 | antisense | chr4:26154262-26197056:- | 1.52 | 8.49 | 2.48 | 0.0020 | 0.0191 |
| ENSG00000274251 | lincRNA | chr11:67353629-67354348:+ | 7.36 | 21.03 | 1.51 | 0.0020 | 0.0191 |
| MSTRG.87758 | linc | chr2:28421598-28423235:+ | 13.52 | 36.24 | 1.42 | 0.0020 | 0.0194 |
| ENSG00000278090 | lincRNA | chr15:99028538-99031053:+ | 4.82 | 25.65 | 2.41 | 0.0021 | 0.0195 |
| MSTRG.19560 | linc | chr10:27557794-27608527:+ | 114.73 | 339.26 | 1.56 | 0.0021 | 0.0196 |
| ENSG00000236226 | antisense | chr7:103030104-103031354:+ | 0.16 | 2.87 | 4.13 | 0.0021 | 0.0199 |
| MSTRG.98106 | antisense | chr2:190694763-190700937:+ | 29.69 | 99.64 | 1.75 | 0.0021 | 0.0202 |
| MSTRG.102592 | linc | chr20:5504620-5506455:- | 2.28 | 9.63 | 2.08 | 0.0021 | 0.0203 |
| MSTRG.114210 | antisense | chr3:24137812-24238822:+ | 1075.10 | 395.53 | -1.44 | 0.0022 | 0.0203 |
| MSTRG.175987 | linc | chr8:122338742-122379443:- | 7.03 | 0.86 | -3.04 | 0.0022 | 0.0203 |
| MSTRG.79321 | linc | chr18:76316498-76319724:- | 14.83 | 42.47 | 1.52 | 0.0022 | 0.0204 |
| MSTRG.363 | antisense | chr1:779512-781826:+ | 73.14 | 33.48 | -1.13 | 0.0022 | 0.0205 |
| MSTRG.92292 | linc | chr2:99515500-99524755:+ | 0.15 | 3.00 | 4.32 | 0.0022 | 0.0208 |
| MSTRG.104420 | linc | chr20:35766854-35768218:- | 6.45 | 20.53 | 1.67 | 0.0022 | 0.0208 |
| MSTRG.61364 | linc | chr15:84807049-84812566:+ | 72.30 | 164.70 | 1.19 | 0.0023 | 0.0210 |
| MSTRG.8564 | antisense | chr1:109025847-109033986:- | 30.72 | 9.53 | -1.69 | 0.0023 | 0.0211 |
| MSTRG.185500 | linc | chr9:128665373-128680368:- | 24.53 | 61.44 | 1.32 | 0.0023 | 0.0211 |
| ENSG00000258418 | lincRNA | chr14:40386252-40386794:+ | 8.92 | 1.04 | -3.09 | 0.0023 | 0.0213 |
| ENSG00000264548 | antisense | chr17:82214227-82217352:+ | 11.52 | 30.69 | 1.41 | 0.0024 | 0.0215 |
| ENSG00000229191 | antisense | chr1:201023949-201028792:+ | 2.26 | 10.28 | 2.19 | 0.0023 | 0.0215 |
| MSTRG.150406 | linc | chr6:31220005-31221419:+ | 2.20 | 17.37 | 2.98 | 0.0024 | 0.0215 |
| MSTRG.54756 | linc | chr14:77503115-77505323:- | 2.62 | 10.32 | 1.98 | 0.0024 | 0.0215 |
| MSTRG.88721 | linc | chr2:42105615-42110646:+ | 60.61 | 153.26 | 1.34 | 0.0024 | 0.0215 |
| ENSG00000234883 | lincRNA | chr21:25561909-25575168:+ | 69.04 | 32.03 | -1.11 | 0.0024 | 0.0215 |
| MSTRG.96243 | antisense | chr2:165801847-165802612:- | 11.99 | 3.66 | -1.71 | 0.0024 | 0.0215 |
| MSTRG.96089 | linc | chr2:159307052-159312559:- | 13.66 | 44.97 | 1.72 | 0.0023 | 0.0215 |
| MSTRG.65832 | antisense | chr16:66471095-66492777:- | 9.75 | 28.53 | 1.55 | 0.0024 | 0.0215 |
| ENSG00000266236 | sense_intronic | chr17:82482098-82483388:+ | 31.78 | 84.11 | 1.40 | 0.0024 | 0.0215 |
| ENSG00000251179 | antisense | chr17:50281577-50287855:- | 4.40 | 21.01 | 2.26 | 0.0024 | 0.0215 |
| MSTRG.67138 | linc | chr16:84558374-84560708:- | 62.86 | 222.34 | 1.82 | 0.0024 | 0.0216 |
| ENSG00000213279 | lincRNA | chr22:49572264-49575426:- | 40.52 | 96.75 | 1.26 | 0.0024 | 0.0216 |
| ENSG00000273368 | sense_intronic | chr18:8154558-8155070:+ | 13.65 | 4.01 | -1.77 | 0.0024 | 0.0216 |
| ENSG00000264007 | sense_intronic | chr17:29621617-29622254:- | 2.16 | 9.05 | 2.07 | 0.0024 | 0.0216 |
| MSTRG.135826 | linc | chr4:183334139-183334525:- | 7.70 | 1.12 | -2.78 | 0.0025 | 0.0218 |
| ENSG00000233096 | lincRNA | chr3:40970541-40971578:- | 5.43 | 0.88 | -2.63 | 0.0025 | 0.0218 |
| MSTRG.163338 | linc | chr7:74280626-74289295:- | 19.00 | 5.03 | -1.92 | 0.0025 | 0.0218 |
| ENSG00000263766 | antisense | chr17:47603860-47649420:- | 44.62 | 101.00 | 1.18 | 0.0025 | 0.0218 |
| ENSG00000248774 | antisense | chr4:173322206-173329694:- | 79.01 | 35.70 | -1.15 | 0.0025 | 0.0222 |
| MSTRG.154348 | linc | chr6:105676852-105892943:- | 3.70 | 14.36 | 1.96 | 0.0026 | 0.0223 |
| MSTRG.137922 | linc | chr5:29969396-29976973:- | 28.54 | 1.75 | -4.03 | 0.0026 | 0.0223 |
| ENSG00000257764 | antisense | chr12:69353493-69354225:- | 1.74 | 20.35 | 3.55 | 0.0026 | 0.0223 |
| MSTRG.16099 | linc | chr1:230540230-230542038:- | 2.15 | 9.40 | 2.13 | 0.0026 | 0.0224 |
| MSTRG.17107 | linc | chr1:244815774-244830975:+ | 58.99 | 120.01 | 1.02 | 0.0026 | 0.0224 |
| MSTRG.24583 | linc | chr10:111147684-111150843:- | 44.44 | 7.08 | -2.65 | 0.0026 | 0.0225 |
| MSTRG.137905 | linc | chr5:29088081-29089443:+ | 0.16 | 2.94 | 4.16 | 0.0026 | 0.0225 |
| MSTRG.122989 | linc | chr3:155187396-155195252:+ | 142.32 | 596.88 | 2.07 | 0.0026 | 0.0225 |
| ENSG00000270210 | lincRNA | chr2:28425945-28426719:+ | 11.35 | 29.95 | 1.40 | 0.0026 | 0.0226 |
| ENSG00000236352 | antisense | chr20:53940160-53942508:+ | 3.03 | 11.14 | 1.88 | 0.0026 | 0.0226 |
| ENSG00000273951 | sense_intronic | chr20:41485571-41486225:- | 62.94 | 28.52 | -1.14 | 0.0026 | 0.0226 |
| MSTRG.31105 | linc | chr11:71657017-71659915:+ | 0.49 | 4.00 | 3.02 | 0.0026 | 0.0226 |
| ENSG00000249173 | lincRNA | chr4:184893871-184899454:- | 1.69 | 18.93 | 3.49 | 0.0026 | 0.0226 |
| MSTRG.67728 | linc | chr16:89968110-89969722:+ | 0.98 | 5.67 | 2.54 | 0.0027 | 0.0226 |
| MSTRG.70819 | antisense | chr17:38765029-38767421:+ | 21.71 | 69.63 | 1.68 | 0.0027 | 0.0227 |
| ENSG00000234222 | antisense | chr1:145926590-145959179:+ | 1.87 | 8.23 | 2.14 | 0.0027 | 0.0227 |
| ENSG00000265888 | antisense | chr18:31101588-31162789:+ | 2.38 | 12.13 | 2.35 | 0.0027 | 0.0230 |
| MSTRG.111395 | linc | chr22:37454828-37463739:- | 46.33 | 100.94 | 1.12 | 0.0027 | 0.0230 |
| MSTRG.72904 | linc | chr17:61935496-61937631:- | 0.95 | 5.43 | 2.52 | 0.0027 | 0.0231 |
| ENSG00000229056 | antisense | chr2:196260024-196264204:+ | 72.25 | 32.60 | -1.15 | 0.0028 | 0.0232 |
| ENSG00000269892 | lincRNA | chr12:6742985-6743641:+ | 6.29 | 18.86 | 1.58 | 0.0028 | 0.0233 |
| MSTRG.180646 | linc | chr9:66259047-66269877:- | 66.47 | 138.68 | 1.06 | 0.0028 | 0.0233 |
| MSTRG.54308 | linc | chr14:73224732-73232041:+ | 54.16 | 129.88 | 1.26 | 0.0028 | 0.0233 |
| MSTRG.83423 | antisense | chr19:41384144-41387737:+ | 18.54 | 59.83 | 1.69 | 0.0028 | 0.0233 |
| ENSG00000238201 | lincRNA | chr2:64338067-64341647:- | 226.46 | 87.05 | -1.38 | 0.0028 | 0.0235 |
| MSTRG.168468 | antisense | chr8:1760235-1762602:+ | 31.12 | 9.39 | -1.73 | 0.0028 | 0.0235 |
| ENSG00000230257 | antisense | chr7:102973522-102988856:+ | 54.25 | 197.22 | 1.86 | 0.0029 | 0.0236 |
| ENSG00000277602 | lincRNA | chr16:1971655-1971896:- | 2.04 | 8.49 | 2.06 | 0.0029 | 0.0239 |
| ENSG00000272682 | lincRNA | chr22:19124309-19128449:- | 0.33 | 3.46 | 3.37 | 0.0029 | 0.0239 |
| MSTRG.10312 | antisense | chr1:151020736-151023427:+ | 2.98 | 10.94 | 1.88 | 0.0029 | 0.0239 |
| MSTRG.184031 | antisense | chr9:110883509-110927695:- | 46.91 | 98.74 | 1.07 | 0.0029 | 0.0239 |
| MSTRG.13753 | antisense | chr1:200387442-200397631:- | 187.19 | 469.04 | 1.33 | 0.0029 | 0.0240 |
| MSTRG.65685 | linc | chr16:66014598-66227765:- | 110.35 | 540.84 | 2.29 | 0.0030 | 0.0240 |
| MSTRG.61698 | antisense | chr15:89821456-89836802:+ | 9.68 | 42.00 | 2.12 | 0.0029 | 0.0240 |
| MSTRG.126297 | antisense | chr4:2731483-2735442:- | 29.44 | 67.72 | 1.20 | 0.0030 | 0.0240 |
| MSTRG.159929 | antisense | chr7:15364983-15366355:+ | 5.53 | 19.87 | 1.85 | 0.0030 | 0.0240 |
| ENSG00000203801 | lincRNA | chr6:108751654-108769942:+ | 4.70 | 15.20 | 1.69 | 0.0030 | 0.0241 |
| ENSG00000246740 | antisense | chr15:41972763-41999094:+ | 6.60 | 1.05 | -2.66 | 0.0029 | 0.0241 |
| MSTRG.71291 | antisense | chr17:42026723-42027293:+ | 0.91 | 5.59 | 2.62 | 0.0030 | 0.0241 |
| MSTRG.60787 | linc | chr15:75144550-75155678:- | 4.62 | 15.30 | 1.73 | 0.0030 | 0.0241 |
| MSTRG.166801 | antisense | chr7:140012852-140018111:- | 12.89 | 34.25 | 1.41 | 0.0030 | 0.0241 |
| ENSG00000267317 | antisense | chr19:1457670-1458580:- | 33.11 | 73.09 | 1.14 | 0.0031 | 0.0247 |
| ENSG00000236242 | antisense | chr13:109163902-109201483:- | 4.13 | 14.27 | 1.79 | 0.0031 | 0.0248 |
| ENSG00000272842 | antisense | chr9:19371386-19371945:- | 20.26 | 7.55 | -1.42 | 0.0031 | 0.0249 |
| MSTRG.16522 | linc | chr1:235916652-235917632:- | 0.33 | 3.34 | 3.32 | 0.0031 | 0.0249 |
| ENSG00000228950 | lincRNA | chr2:20451042-20452947:+ | 18.56 | 6.11 | -1.60 | 0.0031 | 0.0249 |
| ENSG00000272953 | lincRNA | chr7:5425770-5426401:+ | 4.72 | 14.57 | 1.63 | 0.0032 | 0.0249 |
| MSTRG.23885 | antisense | chr10:99699898-99701305:- | 12.36 | 32.07 | 1.38 | 0.0031 | 0.0249 |
| MSTRG.149960 | linc | chr6:26486395-26487739:- | 0.14 | 2.69 | 4.25 | 0.0031 | 0.0249 |
| MSTRG.63398 | antisense | chr16:9127039-9141108:+ | 45.34 | 186.43 | 2.04 | 0.0031 | 0.0250 |
| MSTRG.138609 | antisense | chr5:39894368-39948498:- | 2.80 | 10.62 | 1.92 | 0.0032 | 0.0251 |
| MSTRG.26340 | linc | chr11:1770420-1771876:- | 12.85 | 40.42 | 1.65 | 0.0032 | 0.0251 |
| MSTRG.130115 | linc | chr4:74035472-74074421:+ | 296.80 | 126.85 | -1.23 | 0.0032 | 0.0251 |
| ENSG00000234810 | lincRNA | chr1:55581037-55868248:+ | 28.00 | 7.86 | -1.83 | 0.0032 | 0.0251 |
| MSTRG.110365 | antisense | chr22:29100431-29111356:+ | 60.40 | 476.00 | 2.98 | 0.0032 | 0.0252 |
| MSTRG.159431 | linc | chr7:6103517-6104660:- | 10.09 | 28.29 | 1.49 | 0.0032 | 0.0252 |
| ENSG00000215244 | lincRNA | chr10:6277687-6335982:+ | 145.99 | 425.24 | 1.54 | 0.0032 | 0.0252 |
| MSTRG.35162 | antisense | chr11:126281961-126283538:- | 37.15 | 95.25 | 1.36 | 0.0032 | 0.0252 |
| MSTRG.86528 | linc | chr2:16452019-16478608:- | 19.18 | 5.28 | -1.86 | 0.0033 | 0.0252 |
| MSTRG.171940 | linc | chr8:65930911-65952743:- | 23.27 | 60.67 | 1.38 | 0.0033 | 0.0253 |
| MSTRG.56206 | linc | chr14:102732874-102743845:+ | 136.74 | 341.34 | 1.32 | 0.0033 | 0.0253 |
| ENSG00000259687 | lincRNA | chr14:75294404-75296638:+ | 126.10 | 49.97 | -1.34 | 0.0033 | 0.0253 |
| MSTRG.31488 | antisense | chr11:75810346-75813856:+ | 4.82 | 40.43 | 3.07 | 0.0033 | 0.0254 |
| MSTRG.23644 | antisense | chr10:97346175-97435046:- | 10.95 | 36.63 | 1.74 | 0.0033 | 0.0254 |
| MSTRG.38009 | antisense | chr12:31895068-31959370:- | 197.30 | 624.45 | 1.66 | 0.0033 | 0.0254 |
| MSTRG.72873 | antisense | chr17:59848352-59873899:+ | 192.36 | 630.55 | 1.71 | 0.0033 | 0.0255 |
| MSTRG.151728 | antisense | chr6:43610907-43617554:- | 68.40 | 137.68 | 1.01 | 0.0033 | 0.0255 |
| MSTRG.64596 | linc | chr16:30804550-30815395:+ | 3.58 | 12.91 | 1.85 | 0.0033 | 0.0255 |
| MSTRG.183249 | linc | chr9:105036316-105061582:- | 21.57 | 143.97 | 2.74 | 0.0034 | 0.0256 |
| MSTRG.43641 | antisense | chr12:109900621-109924248:- | 127.71 | 337.78 | 1.40 | 0.0034 | 0.0256 |
| MSTRG.365 | antisense | chr1:789473-789873:+ | 5.34 | 0.98 | -2.44 | 0.0035 | 0.0261 |
| MSTRG.126902 | linc | chr4:9636874-9638346:+ | 0.15 | 2.75 | 4.19 | 0.0035 | 0.0262 |
| ENSG00000266980 | antisense | chr17:75818815-75820055:- | 8.78 | 23.63 | 1.43 | 0.0035 | 0.0262 |
| ENSG00000274213 | lincRNA | chr17:56914186-56914533:+ | 24.47 | 88.05 | 1.85 | 0.0035 | 0.0262 |
| ENSG00000229807 | lincRNA | chrX:73820651-73852753:- | 21283.79 | 3508.42 | -2.60 | 0.0035 | 0.0266 |
| MSTRG.68254 | linc | chr17:4645765-4647270:+ | 0.33 | 3.36 | 3.33 | 0.0036 | 0.0268 |
| MSTRG.50215 | antisense | chr13:110637064-110637837:- | 9.33 | 29.78 | 1.67 | 0.0036 | 0.0269 |
| MSTRG.80004 | antisense | chr19:3159496-3169946:- | 12.92 | 68.98 | 2.42 | 0.0036 | 0.0269 |
| MSTRG.31016 | antisense | chr11:70384357-70385607:- | 49.51 | 16.51 | -1.58 | 0.0036 | 0.0270 |
| MSTRG.106005 | linc | chr20:61951185-61952465:- | 2.65 | 11.11 | 2.07 | 0.0036 | 0.0270 |
| MSTRG.59674 | antisense | chr15:65895307-65897459:+ | 57.67 | 117.58 | 1.03 | 0.0036 | 0.0270 |
| ENSG00000267365 | antisense | chr17:70166961-70169402:- | 37.58 | 137.62 | 1.87 | 0.0037 | 0.0270 |
| ENSG00000230606 | lincRNA | chr2:97416165-97433527:- | 272.01 | 123.77 | -1.14 | 0.0036 | 0.0270 |
| ENSG00000233901 | lincRNA | chr9:129332300-129359538:+ | 32.75 | 76.60 | 1.23 | 0.0037 | 0.0271 |
| MSTRG.53707 | linc | chr14:64674107-64703933:+ | 4.61 | 20.18 | 2.13 | 0.0037 | 0.0275 |
| ENSG00000274104 | lincRNA | chr19:34733298-34733837:- | 26.73 | 10.31 | -1.37 | 0.0037 | 0.0275 |
| MSTRG.100348 | linc | chr2:218152496-218156780:- | 28.04 | 122.36 | 2.13 | 0.0038 | 0.0275 |
| MSTRG.57257 | antisense | chr15:30812509-30815785:- | 52.30 | 23.69 | -1.14 | 0.0038 | 0.0279 |
| MSTRG.93209 | linc | chr2:112669816-112695808:+ | 53.05 | 141.73 | 1.42 | 0.0038 | 0.0279 |
| MSTRG.46349 | linc | chr13:30793004-30800958:+ | 8.40 | 26.57 | 1.66 | 0.0038 | 0.0279 |
| MSTRG.100318 | linc | chr2:218187313-218191342:+ | 1.91 | 20.18 | 3.40 | 0.0038 | 0.0279 |
| MSTRG.91222 | linc | chr2:75239094-75416833:+ | 135.52 | 50.00 | -1.44 | 0.0038 | 0.0279 |
| ENSG00000237798 | antisense | chr2:174575227-174587726:+ | 0.59 | 4.11 | 2.79 | 0.0039 | 0.0281 |
| ENSG00000261441 | antisense | chr15:89335053-89336161:+ | 2.51 | 8.86 | 1.82 | 0.0039 | 0.0281 |
| MSTRG.72535 | antisense | chr17:57909239-57933850:- | 76.18 | 207.18 | 1.44 | 0.0039 | 0.0281 |
| MSTRG.30565 | linc | chr11:65574460-65575811:+ | 33.58 | 73.61 | 1.13 | 0.0039 | 0.0282 |
| ENSG00000268262 | sense_overlapping | chr19:39314651-39320858:- | 5.43 | 24.43 | 2.17 | 0.0039 | 0.0284 |
| MSTRG.111444 | linc | chr22:37895975-37897670:+ | 0.48 | 3.83 | 3.01 | 0.0040 | 0.0285 |
| MSTRG.36268 | linc | chr12:7982061-7984033:+ | 0.46 | 4.05 | 3.14 | 0.0040 | 0.0285 |
| MSTRG.175404 | antisense | chr8:116651989-116681380:+ | 62.22 | 230.20 | 1.89 | 0.0040 | 0.0285 |
| MSTRG.36525 | linc | chr12:10579694-10585205:- | 116.34 | 55.39 | -1.07 | 0.0040 | 0.0285 |
| MSTRG.23730 | linc | chr10:97299936-97308806:- | 5.96 | 24.33 | 2.03 | 0.0040 | 0.0286 |
| ENSG00000234506 | lincRNA | chr9:68543541-68546589:- | 25.31 | 162.06 | 2.68 | 0.0040 | 0.0286 |
| MSTRG.122637 | linc | chr3:151932414-151969586:- | 0.60 | 4.21 | 2.81 | 0.0040 | 0.0286 |
| MSTRG.111972 | antisense | chr22:43109567-43111354:- | 1.00 | 5.11 | 2.36 | 0.0040 | 0.0286 |
| ENSG00000255864 | lincRNA | chr12:24213256-24562590:- | 23.06 | 9.43 | -1.29 | 0.0041 | 0.0287 |
| MSTRG.78689 | linc | chr18:65236527-65254916:+ | 9.36 | 1.78 | -2.40 | 0.0041 | 0.0287 |
| ENSG00000237094 | lincRNA | chr1:365389-501617:- | 65.08 | 186.35 | 1.52 | 0.0041 | 0.0287 |
| ENSG00000235908 | sense_intronic | chr3:49365145-49367006:- | 13.40 | 34.19 | 1.35 | 0.0041 | 0.0288 |
| MSTRG.36296 | linc | chr12:7954220-7972939:+ | 11.66 | 29.43 | 1.34 | 0.0041 | 0.0288 |
| MSTRG.45836 | linc | chr13:25243093-25245711:- | 28.12 | 64.73 | 1.20 | 0.0041 | 0.0288 |
| MSTRG.126872 | linc | chr4:9073928-9078879:+ | 2.70 | 0.11 | -4.65 | 0.0041 | 0.0288 |
| ENSG00000228485 | sense_intronic | chr10:119208531-119211760:+ | 65.72 | 32.74 | -1.01 | 0.0041 | 0.0288 |
| MSTRG.91759 | linc | chr2:88659020-88688583:+ | 168.85 | 49.91 | -1.76 | 0.0041 | 0.0288 |
| ENSG00000280543 | sense_intronic | chr8:130082738-130084768:- | 70.82 | 163.87 | 1.21 | 0.0041 | 0.0288 |
| ENSG00000233013 | lincRNA | chr9:138216187-138252994:+ | 250.68 | 825.65 | 1.72 | 0.0042 | 0.0289 |
| ENSG00000205018 | antisense | chr16:88939789-88951524:+ | 5.45 | 15.89 | 1.54 | 0.0041 | 0.0289 |
| ENSG00000249096 | lincRNA | chr4:184365183-184382306:- | 137.16 | 290.31 | 1.08 | 0.0042 | 0.0293 |
| ENSG00000273141 | antisense | chr18:11908712-11909223:+ | 17.76 | 41.60 | 1.23 | 0.0043 | 0.0296 |
| MSTRG.185049 | linc | chr9:124181701-124188068:- | 0.31 | 3.30 | 3.39 | 0.0043 | 0.0296 |
| ENSG00000273133 | antisense | chr4:15563698-15564253:- | 28.38 | 83.53 | 1.56 | 0.0044 | 0.0300 |
| MSTRG.1252 | linc | chr1:15821694-15824166:- | 2.30 | 14.27 | 2.63 | 0.0044 | 0.0301 |
| MSTRG.3528 | linc | chr1:43372242-43372779:+ | 1.97 | 8.17 | 2.05 | 0.0044 | 0.0302 |
| MSTRG.29847 | antisense | chr11:59826137-59835131:+ | 13.66 | 64.98 | 2.25 | 0.0044 | 0.0302 |
| ENSG00000257599 | antisense | chr12:29389294-29487488:+ | 12.94 | 38.71 | 1.58 | 0.0044 | 0.0303 |
| ENSG00000249898 | antisense | chr8:6618475-6708209:- | 104.69 | 43.72 | -1.26 | 0.0045 | 0.0305 |
| MSTRG.18926 | antisense | chr10:17406098-17412006:- | 26.56 | 10.52 | -1.34 | 0.0045 | 0.0305 |
| MSTRG.121783 | antisense | chr3:136862215-136862710:- | 1.11 | 5.29 | 2.26 | 0.0045 | 0.0306 |
| ENSG00000258777 | lincRNA | chr14:61681041-61695823:- | 1.43 | 6.50 | 2.19 | 0.0046 | 0.0312 |
| MSTRG.151509 | antisense | chr6:42693633-42701330:+ | 219.98 | 472.12 | 1.10 | 0.0046 | 0.0312 |
| MSTRG.148669 | linc | chr6:6885640-6898485:+ | 5.85 | 21.59 | 1.88 | 0.0046 | 0.0312 |
| MSTRG.112444 | linc | chr22:49770626-49771153:- | 4.89 | 17.78 | 1.86 | 0.0046 | 0.0313 |
| MSTRG.108716 | linc | chr21:43034702-43046743:+ | 86.26 | 192.36 | 1.16 | 0.0047 | 0.0314 |
| MSTRG.164865 | antisense | chr7:100477741-100479703:+ | 7.10 | 19.30 | 1.44 | 0.0047 | 0.0316 |
| ENSG00000273143 | lincRNA | chr10:110428840-110496204:- | 12.59 | 3.18 | -1.99 | 0.0047 | 0.0316 |
| MSTRG.183250 | linc | chr9:105045268-105049958:- | 10.58 | 77.31 | 2.87 | 0.0047 | 0.0316 |
| MSTRG.56156 | linc | chr14:101939709-101941727:+ | 2.24 | 0.11 | -4.38 | 0.0047 | 0.0316 |
| ENSG00000261101 | sense_overlapping | chrX:101627868-101628523:+ | 65.82 | 31.67 | -1.06 | 0.0047 | 0.0317 |
| MSTRG.20428 | antisense | chr10:12123852-12187967:+ | 41.73 | 87.01 | 1.06 | 0.0047 | 0.0317 |
| MSTRG.159049 | linc | chr7:2402645-2403276:- | 0.32 | 3.17 | 3.30 | 0.0047 | 0.0317 |
| ENSG00000277767 | lincRNA | chr13:110916004-110917827:+ | 55.11 | 24.50 | -1.17 | 0.0048 | 0.0318 |
| ENSG00000223571 | sense_intronic | chrX:2334295-2336410:- | 7.71 | 22.36 | 1.54 | 0.0048 | 0.0320 |
| MSTRG.73805 | antisense | chr17:74520798-74535561:+ | 61.78 | 131.02 | 1.08 | 0.0049 | 0.0322 |
| MSTRG.47618 | linc | chr13:48567368-48570390:- | 3.46 | 12.73 | 1.88 | 0.0049 | 0.0323 |
| ENSG00000272941 | antisense | chr7:135168403-135169547:+ | 243.33 | 596.33 | 1.29 | 0.0049 | 0.0323 |
| MSTRG.138979 | linc | chr5:50445504-50560103:+ | 1.47 | 18.34 | 3.65 | 0.0049 | 0.0325 |
| ENSG00000225850 | antisense | chr10:97334564-97343203:+ | 2.78 | 10.22 | 1.88 | 0.0049 | 0.0325 |
| MSTRG.53412 | antisense | chr14:58205620-58207041:+ | 30.82 | 12.65 | -1.28 | 0.0050 | 0.0325 |
| ENSG00000240893 | lincRNA | chr3:112736447-112749319:- | 10.26 | 2.93 | -1.81 | 0.0049 | 0.0325 |
| ENSG00000272010 | lincRNA | chr8:65591850-65592472:- | 8.26 | 2.27 | -1.86 | 0.0050 | 0.0326 |
| ENSG00000273117 | lincRNA | chr7:155295918-155297541:- | 86.65 | 42.37 | -1.03 | 0.0050 | 0.0327 |
| ENSG00000275426 | sense_intronic | chr4:149738-150317:+ | 37.12 | 16.07 | -1.21 | 0.0050 | 0.0327 |
| MSTRG.89605 | antisense | chr2:54957708-54961159:- | 10.98 | 38.91 | 1.83 | 0.0050 | 0.0327 |
| MSTRG.187193 | linc | chrX:1923372-2216817:- | 729.55 | 1641.65 | 1.17 | 0.0050 | 0.0328 |
| MSTRG.5192 | antisense | chr1:63538778-63544873:+ | 21.16 | 8.25 | -1.36 | 0.0051 | 0.0329 |
| MSTRG.65299 | linc | chr16:53336741-53355399:- | 17.49 | 79.02 | 2.18 | 0.0051 | 0.0329 |
| MSTRG.170267 | antisense | chr8:28770038-28771987:+ | 21.07 | 49.68 | 1.24 | 0.0051 | 0.0330 |
| MSTRG.33371 | linc | chr11:109946542-109972994:+ | 5.95 | 0.96 | -2.63 | 0.0051 | 0.0331 |
| ENSG00000214188 | sense_intronic | chr7:116953899-117098806:+ | 37.53 | 17.25 | -1.12 | 0.0052 | 0.0332 |
| MSTRG.109904 | antisense | chr22:22732422-22759623:- | 73.22 | 27.71 | -1.40 | 0.0051 | 0.0332 |
| ENSG00000264254 | lincRNA | chr18:5310396-5317664:+ | 8.30 | 2.12 | -1.97 | 0.0052 | 0.0334 |
| ENSG00000268555 | lincRNA | chr19:21570822-21587322:- | 150.09 | 69.01 | -1.12 | 0.0052 | 0.0334 |
| ENSG00000235527 | antisense | chr1:113924000-113929492:- | 71.28 | 33.80 | -1.08 | 0.0052 | 0.0334 |
| ENSG00000255364 | lincRNA | chr8:122414332-122428551:- | 29.71 | 10.08 | -1.56 | 0.0052 | 0.0335 |
| MSTRG.151476 | linc | chr6:41174296-41179096:- | 7.90 | 24.17 | 1.61 | 0.0053 | 0.0336 |
| ENSG00000225195 | sense_intronic | chr12:64628344-64629976:+ | 23.70 | 56.28 | 1.25 | 0.0053 | 0.0336 |
| MSTRG.127367 | antisense | chr4:10143081-10146587:+ | 3.58 | 12.03 | 1.75 | 0.0053 | 0.0337 |
| MSTRG.84398 | linc | chr19:49549673-49554377:- | 8.81 | 28.24 | 1.68 | 0.0053 | 0.0338 |
| MSTRG.184314 | antisense | chr9:112483558-112484577:+ | 2.21 | 8.63 | 1.97 | 0.0054 | 0.0341 |
| ENSG00000261229 | lincRNA | chr15:79843547-79844304:- | 19.54 | 58.37 | 1.58 | 0.0054 | 0.0341 |
| ENSG00000276867 | lincRNA | chr16:31704728-31705260:+ | 16.60 | 5.91 | -1.49 | 0.0054 | 0.0341 |
| MSTRG.123241 | linc | chr3:158923871-158930980:+ | 9.61 | 27.84 | 1.54 | 0.0054 | 0.0341 |
| ENSG00000278462 | sense_intronic | chr13:46717423-46717688:+ | 35.10 | 14.27 | -1.30 | 0.0054 | 0.0342 |
| ENSG00000257878 | antisense | chr12:95996521-96011489:+ | 101.01 | 347.59 | 1.78 | 0.0054 | 0.0342 |
| ENSG00000258168 | antisense | chr12:70468080-70543040:+ | 77.66 | 37.59 | -1.05 | 0.0054 | 0.0343 |
| ENSG00000261736 | sense_intronic | chr16:27643199-27644663:+ | 4.15 | 12.80 | 1.63 | 0.0055 | 0.0343 |
| ENSG00000241666 | antisense | chr1:167627385-167630674:- | 17.93 | 41.68 | 1.22 | 0.0055 | 0.0344 |
| ENSG00000179840 | antisense | chr1:9652610-9654586:- | 12.33 | 29.89 | 1.28 | 0.0055 | 0.0345 |
| ENSG00000255337 | antisense | chr11:102452919-102462008:+ | 0.35 | 2.99 | 3.10 | 0.0055 | 0.0345 |
| ENSG00000213373 | lincRNA | chr17:42874670-42898704:- | 7.42 | 24.79 | 1.74 | 0.0056 | 0.0348 |
| MSTRG.163349 | antisense | chr7:74218306-74231068:- | 0.50 | 5.77 | 3.52 | 0.0056 | 0.0348 |
| ENSG00000260361 | sense_intronic | chr15:92779757-92781492:- | 8.74 | 31.10 | 1.83 | 0.0056 | 0.0349 |
| ENSG00000273123 | antisense | chr3:123715851-123716399:+ | 10.25 | 2.48 | -2.05 | 0.0056 | 0.0349 |
| MSTRG.109665 | linc | chr22:22093541-22094781:- | 2.27 | 0.18 | -3.64 | 0.0056 | 0.0349 |
| MSTRG.72882 | linc | chr17:59855130-59856647:- | 0.44 | 3.71 | 3.08 | 0.0057 | 0.0352 |
| MSTRG.90771 | antisense | chr2:70553217-70569779:+ | 0.15 | 2.58 | 4.10 | 0.0057 | 0.0352 |
| ENSG00000278231 | lincRNA | chr20:48821688-48849458:- | 107.15 | 332.68 | 1.63 | 0.0057 | 0.0352 |
| MSTRG.53907 | linc | chr14:68800949-68825967:- | 283.73 | 580.04 | 1.03 | 0.0057 | 0.0352 |
| MSTRG.152694 | antisense | chr6:70413740-70415290:+ | 54.04 | 23.86 | -1.18 | 0.0057 | 0.0352 |
| MSTRG.66804 | linc | chr16:79842777-79845858:- | 22.14 | 6.54 | -1.76 | 0.0058 | 0.0355 |
| MSTRG.67234 | antisense | chr16:85759375-85774281:+ | 12.30 | 54.72 | 2.15 | 0.0058 | 0.0355 |
| MSTRG.47847 | antisense | chr13:51834658-51845497:+ | 50.63 | 244.37 | 2.27 | 0.0058 | 0.0355 |
| MSTRG.2440 | linc | chr1:30101880-30130717:- | 19.99 | 138.35 | 2.79 | 0.0058 | 0.0357 |
| MSTRG.73235 | linc | chr17:67814820-67816335:- | 0.18 | 2.90 | 3.97 | 0.0059 | 0.0358 |
| MSTRG.60245 | antisense | chr15:63883107-63901197:- | 2.51 | 12.47 | 2.31 | 0.0059 | 0.0358 |
| ENSG00000224616 | antisense | chr1:100264009-100266120:- | 57.20 | 26.80 | -1.09 | 0.0059 | 0.0361 |
| ENSG00000225778 | antisense | chr10:11849608-11894700:- | 4.53 | 0.53 | -3.09 | 0.0060 | 0.0362 |
| MSTRG.42366 | linc | chr12:94486854-94490629:- | 4.05 | 20.22 | 2.32 | 0.0060 | 0.0363 |
| MSTRG.126029 | linc | chr4:1690113-1690773:- | 1.24 | 6.15 | 2.31 | 0.0060 | 0.0363 |
| ENSG00000225623 | sense_intronic | chr1:49374201-49472085:- | 2.18 | 0.11 | -4.28 | 0.0060 | 0.0365 |
| MSTRG.67188 | antisense | chr16:84915668-84917877:+ | 1.28 | 10.36 | 3.01 | 0.0061 | 0.0365 |
| ENSG00000272983 | lincRNA | chr10:38137337-38144399:+ | 28.27 | 12.29 | -1.20 | 0.0061 | 0.0365 |
| ENSG00000253230 | lincRNA | chr8:9900064-9905366:- | 2.74 | 0.12 | -4.52 | 0.0061 | 0.0368 |
| ENSG00000245008 | antisense | chr11:128629653-128686922:- | 18.38 | 5.88 | -1.65 | 0.0061 | 0.0368 |
| ENSG00000246448 | antisense | chr4:143700257-143865072:+ | 136.15 | 52.52 | -1.37 | 0.0061 | 0.0368 |
| MSTRG.168092 | linc | chr7:155787444-155788573:+ | 3.46 | 17.03 | 2.30 | 0.0062 | 0.0368 |
| MSTRG.176787 | linc | chr8:130031130-130049219:- | 123.67 | 513.81 | 2.05 | 0.0062 | 0.0368 |
| MSTRG.115346 | antisense | chr3:38098940-38100294:- | 2.27 | 13.24 | 2.55 | 0.0062 | 0.0368 |
| ENSG00000248996 | antisense | chr5:177494995-177503647:+ | 0.98 | 4.95 | 2.34 | 0.0062 | 0.0371 |
| ENSG00000226070 | lincRNA | chr6:40358744-40369474:- | 3.31 | 0.49 | -2.74 | 0.0062 | 0.0371 |
| MSTRG.124639 | linc | chr3:183813638-183814751:- | 1.75 | 7.23 | 2.04 | 0.0062 | 0.0371 |
| ENSG00000260306 | lincRNA | chr16:21794095-21795759:- | 50.60 | 24.00 | -1.08 | 0.0063 | 0.0371 |
| MSTRG.106636 | linc | chr21:13850456-13903758:- | 2905.98 | 725.88 | -2.00 | 0.0063 | 0.0371 |
| ENSG00000267905 | antisense | chr19:51340695-51344117:+ | 2.52 | 0.30 | -3.06 | 0.0063 | 0.0371 |
| ENSG00000229855 | lincRNA | chr5:121199704-121357398:+ | 6.56 | 1.59 | -2.05 | 0.0063 | 0.0372 |
| ENSG00000233338 | antisense | chrX:12902817-12908333:- | 1.41 | 6.27 | 2.15 | 0.0063 | 0.0372 |
| ENSG00000254138 | antisense | chr5:31093977-31267610:- | 29.32 | 8.40 | -1.80 | 0.0063 | 0.0373 |
| MSTRG.15325 | linc | chr1:223481658-223482824:- | 2.09 | 0.09 | -4.50 | 0.0063 | 0.0373 |
| MSTRG.68116 | linc | chr17:1629558-1629982:- | 0.77 | 6.67 | 3.11 | 0.0065 | 0.0376 |
| MSTRG.10494 | antisense | chr1:153797688-153800298:- | 15.36 | 40.06 | 1.38 | 0.0065 | 0.0376 |
| MSTRG.20108 | antisense | chr10:38003940-38011295:- | 1.00 | 5.66 | 2.51 | 0.0065 | 0.0376 |
| MSTRG.39144 | linc | chr12:51220038-51233251:+ | 11.23 | 3.05 | -1.88 | 0.0065 | 0.0377 |
| MSTRG.44266 | antisense | chr12:118019020-118023386:- | 6.19 | 17.81 | 1.53 | 0.0064 | 0.0377 |
| MSTRG.81117 | antisense | chr19:14080410-14105537:+ | 25.82 | 10.23 | -1.34 | 0.0065 | 0.0377 |
| MSTRG.138366 | antisense | chr5:35707729-35708576:+ | 6.11 | 1.50 | -2.02 | 0.0065 | 0.0377 |
| ENSG00000233429 | antisense | chr7:27095647-27100265:+ | 23.30 | 53.58 | 1.20 | 0.0064 | 0.0377 |
| ENSG00000248724 | antisense | chr3:132721750-132874223:+ | 3.10 | 0.47 | -2.73 | 0.0065 | 0.0377 |
| MSTRG.80667 | linc | chr19:8405802-8407678:+ | 6.68 | 19.29 | 1.53 | 0.0066 | 0.0377 |
| ENSG00000278869 | lincRNA | chr22:49933198-49934074:- | 29.07 | 62.87 | 1.11 | 0.0064 | 0.0377 |
| ENSG00000251399 | lincRNA | chr4:79596542-79597173:+ | 9.45 | 2.02 | -2.23 | 0.0065 | 0.0378 |
| MSTRG.39289 | linc | chr12:53370289-53371245:- | 14.08 | 36.55 | 1.38 | 0.0065 | 0.0378 |
| MSTRG.112472 | linc | chr22:49934126-49935376:- | 52.23 | 143.89 | 1.46 | 0.0066 | 0.0378 |
| MSTRG.21886 | antisense | chr10:71803877-71806538:- | 47.71 | 101.32 | 1.09 | 0.0066 | 0.0379 |
| MSTRG.90498 | linc | chr2:68848935-68849636:+ | 1.26 | 5.94 | 2.23 | 0.0066 | 0.0379 |
| MSTRG.150427 | linc | chr6:31563901-31566008:- | 9.80 | 24.95 | 1.35 | 0.0066 | 0.0379 |
| MSTRG.128666 | antisense | chr4:38791186-38796841:+ | 0.13 | 2.41 | 4.17 | 0.0066 | 0.0379 |
| ENSG00000262714 | lincRNA | chr16:53386944-53389085:+ | 126.25 | 342.11 | 1.44 | 0.0066 | 0.0379 |
| ENSG00000228719 | lincRNA | chr22:36445395-36454944:- | 8.71 | 32.11 | 1.88 | 0.0067 | 0.0380 |
| MSTRG.71872 | antisense | chr17:47321064-47322865:+ | 17.06 | 4.71 | -1.86 | 0.0067 | 0.0381 |
| ENSG00000256706 | antisense | chr12:1917951-1922867:+ | 1.54 | 6.36 | 2.05 | 0.0067 | 0.0381 |
| ENSG00000259278 | lincRNA | chr15:39019233-39024918:+ | 14.54 | 5.07 | -1.52 | 0.0067 | 0.0382 |
| ENSG00000274827 | lincRNA | chr14:19344578-19384587:- | 4.13 | 0.91 | -2.19 | 0.0068 | 0.0386 |
| MSTRG.142436 | linc | chr5:100790644-100904659:- | 19.79 | 58.14 | 1.55 | 0.0068 | 0.0386 |
| MSTRG.139168 | linc | chr5:55042975-55062514:- | 61.14 | 28.25 | -1.11 | 0.0068 | 0.0387 |
| MSTRG.163353 | linc | chr7:74254505-74258211:+ | 7.71 | 20.69 | 1.42 | 0.0069 | 0.0387 |
| ENSG00000259642 | antisense | chr15:79922771-79926993:+ | 117.99 | 240.13 | 1.03 | 0.0069 | 0.0388 |
| MSTRG.116691 | antisense | chr3:52218973-52226065:+ | 13.35 | 31.88 | 1.26 | 0.0069 | 0.0388 |
| MSTRG.108602 | linc | chr21:42446035-42456563:- | 104.76 | 38.94 | -1.43 | 0.0069 | 0.0388 |
| ENSG00000272037 | antisense | chr8:102256392-102257821:+ | 6.87 | 18.19 | 1.40 | 0.0069 | 0.0388 |
| ENSG00000269906 | sense_intronic | chr14:50662511-50663178:- | 23.40 | 9.79 | -1.26 | 0.0070 | 0.0391 |
| ENSG00000269921 | lincRNA | chr4:56387625-56388153:+ | 36.74 | 16.04 | -1.20 | 0.0070 | 0.0391 |
| MSTRG.99520 | linc | chr2:207809688-207818178:- | 17.54 | 42.02 | 1.26 | 0.0070 | 0.0393 |
| MSTRG.71753 | linc | chr17:45490885-45491658:+ | 7.49 | 20.27 | 1.44 | 0.0071 | 0.0394 |
| MSTRG.55358 | antisense | chr14:91227593-91233771:+ | 434.76 | 1005.00 | 1.21 | 0.0071 | 0.0395 |
| MSTRG.118059 | linc | chr3:80603923-80653000:- | 12.74 | 2.05 | -2.63 | 0.0071 | 0.0395 |
| MSTRG.30474 | linc | chr11:65477609-65484601:+ | 3.59 | 11.55 | 1.69 | 0.0071 | 0.0395 |
| MSTRG.68170 | linc | chr17:2402305-2403269:+ | 15.19 | 43.52 | 1.52 | 0.0071 | 0.0395 |
| ENSG00000233875 | antisense | chr1:154579065-154579663:- | 17.42 | 40.58 | 1.22 | 0.0071 | 0.0395 |
| MSTRG.195377 | antisense | chrX:155547303-155548689:- | 41.48 | 84.98 | 1.03 | 0.0071 | 0.0395 |
| MSTRG.34722 | linc | chr11:122820351-122824122:+ | 19.60 | 46.95 | 1.26 | 0.0072 | 0.0397 |
| MSTRG.71881 | antisense | chr17:47532654-47598828:- | 87.27 | 228.98 | 1.39 | 0.0072 | 0.0398 |
| ENSG00000232411 | lincRNA | chr2:165833048-165839098:- | 29.49 | 13.41 | -1.14 | 0.0072 | 0.0399 |
| ENSG00000272669 | antisense | chr22:38742625-38743115:+ | 29.73 | 85.53 | 1.52 | 0.0073 | 0.0403 |
| MSTRG.150962 | antisense | chr6:35732133-35735076:+ | 30.58 | 79.56 | 1.38 | 0.0073 | 0.0403 |
| MSTRG.30539 | antisense | chr11:65960220-65961974:- | 13.11 | 30.38 | 1.21 | 0.0074 | 0.0404 |
| MSTRG.94162 | antisense | chr2:127445820-127461574:- | 8.54 | 22.87 | 1.42 | 0.0074 | 0.0404 |
| MSTRG.95088 | linc | chr2:144662307-144663366:- | 11.57 | 1.84 | -2.66 | 0.0074 | 0.0404 |
| MSTRG.72349 | linc | chr17:55666216-55673222:- | 0.66 | 4.14 | 2.64 | 0.0074 | 0.0405 |
| ENSG00000259345 | lincRNA | chr15:38865322-39427195:- | 17.19 | 6.02 | -1.51 | 0.0074 | 0.0406 |
| MSTRG.179210 | antisense | chr9:32406755-32411484:- | 5.15 | 15.21 | 1.56 | 0.0075 | 0.0408 |
| ENSG00000236345 | antisense | chr6:63806836-63822642:+ | 34.12 | 90.94 | 1.41 | 0.0076 | 0.0411 |
| ENSG00000259747 | lincRNA | chr15:38671847-38689191:+ | 11.41 | 3.91 | -1.54 | 0.0076 | 0.0414 |
| MSTRG.177323 | antisense | chr8:141326825-141327576:- | 0.32 | 9.50 | 4.91 | 0.0077 | 0.0415 |
| ENSG00000269930 | lincRNA | chr15:30616958-30617749:+ | 10.00 | 3.14 | -1.67 | 0.0077 | 0.0416 |
| ENSG00000253284 | sense_intronic | chr12:19147074-19154659:+ | 49.24 | 20.50 | -1.26 | 0.0077 | 0.0416 |
| MSTRG.102345 | linc | chr20:1711037-1746705:- | 47.76 | 158.89 | 1.73 | 0.0077 | 0.0417 |
| MSTRG.556 | linc | chr1:7863299-7913280:- | 208.79 | 764.87 | 1.87 | 0.0077 | 0.0417 |
| ENSG00000237927 | lincRNA | chr6:159586955-159589169:- | 11.86 | 37.46 | 1.66 | 0.0078 | 0.0417 |
| MSTRG.66247 | linc | chr16:69969747-69972225:+ | 0.58 | 3.90 | 2.74 | 0.0078 | 0.0418 |
| MSTRG.90917 | linc | chr2:69943503-69957800:- | 175.74 | 477.35 | 1.44 | 0.0078 | 0.0419 |
| MSTRG.98840 | linc | chr2:197484857-197486406:- | 35.08 | 16.55 | -1.08 | 0.0078 | 0.0419 |
| ENSG00000259004 | lincRNA | chr14:101120856-101123542:+ | 6.87 | 18.44 | 1.42 | 0.0078 | 0.0419 |
| MSTRG.53712 | antisense | chr14:64750701-64758924:+ | 239.05 | 506.12 | 1.08 | 0.0078 | 0.0419 |
| ENSG00000243273 | antisense | chr3:150890636-151038818:+ | 6.80 | 20.36 | 1.58 | 0.0079 | 0.0420 |
| ENSG00000280604 | sense_overlapping | chr21:45914296-45919483:+ | 9.15 | 25.97 | 1.51 | 0.0079 | 0.0420 |
| MSTRG.167006 | antisense | chr7:142927681-142934931:+ | 10.83 | 50.61 | 2.22 | 0.0079 | 0.0420 |
| MSTRG.12856 | linc | chr1:182831361-182837315:+ | 317.83 | 679.97 | 1.10 | 0.0079 | 0.0420 |
| MSTRG.20447 | linc | chr10:12282030-12304265:+ | 0.48 | 3.56 | 2.88 | 0.0079 | 0.0421 |
| MSTRG.41831 | antisense | chr12:89281065-89309777:+ | 0.80 | 4.50 | 2.49 | 0.0079 | 0.0421 |
| MSTRG.110430 | antisense | chr22:29730163-29730684:- | 0.48 | 4.14 | 3.11 | 0.0080 | 0.0422 |
| ENSG00000267551 | antisense | chr19:3141576-3155175:- | 3.17 | 10.17 | 1.68 | 0.0080 | 0.0422 |
| MSTRG.67600 | linc | chr16:88539974-88556470:+ | 107.87 | 250.19 | 1.21 | 0.0080 | 0.0422 |
| ENSG00000179082 | lincRNA | chr9:129321016-129324905:+ | 30.23 | 64.12 | 1.08 | 0.0081 | 0.0423 |
| MSTRG.64597 | linc | chr16:30804907-30809600:+ | 10.01 | 25.83 | 1.37 | 0.0080 | 0.0423 |
| MSTRG.78489 | linc | chr18:61196279-61254957:- | 5.86 | 1.20 | -2.29 | 0.0081 | 0.0423 |
| ENSG00000250348 | antisense | chr5:76285542-76311462:- | 42.04 | 13.77 | -1.61 | 0.0080 | 0.0424 |
| ENSG00000273176 | antisense | chr22:35298838-35299541:- | 22.62 | 8.91 | -1.34 | 0.0081 | 0.0425 |
| MSTRG.161948 | antisense | chr7:38010674-38071004:+ | 19.64 | 6.40 | -1.62 | 0.0081 | 0.0426 |
| MSTRG.135825 | linc | chr4:183332270-183333587:- | 14.22 | 2.50 | -2.51 | 0.0082 | 0.0427 |
| MSTRG.108921 | linc | chr21:44884204-44885494:- | 8.91 | 23.33 | 1.39 | 0.0082 | 0.0427 |
| ENSG00000264707 | lincRNA | chr18:6256747-6260934:+ | 6.85 | 1.37 | -2.32 | 0.0082 | 0.0428 |
| ENSG00000235513 | antisense | chr22:41209122-41217627:- | 51.71 | 21.05 | -1.30 | 0.0082 | 0.0428 |
| ENSG00000267316 | lincRNA | chr18:61571342-61579456:- | 20.78 | 7.65 | -1.44 | 0.0083 | 0.0431 |
| ENSG00000270141 | lincRNA | chr3:169764520-169765060:- | 6.78 | 1.80 | -1.91 | 0.0084 | 0.0434 |
| MSTRG.84184 | linc | chr19:47281235-47282667:+ | 0.73 | 4.53 | 2.64 | 0.0084 | 0.0434 |
| ENSG00000228661 | antisense | chr11:3854318-3855509:- | 3.23 | 9.99 | 1.63 | 0.0084 | 0.0437 |
| MSTRG.65930 | antisense | chr16:67237056-67237404:+ | 4.57 | 13.35 | 1.55 | 0.0084 | 0.0437 |
| MSTRG.13431 | antisense | chr1:193756234-193757080:+ | 25.68 | 9.54 | -1.43 | 0.0085 | 0.0438 |
| ENSG00000270133 | sense_intronic | chr5:94611906-94618122:- | 11.25 | 29.40 | 1.39 | 0.0085 | 0.0438 |
| ENSG00000203721 | lincRNA | chr1:200253419-200400705:- | 61.95 | 131.04 | 1.08 | 0.0085 | 0.0438 |
| MSTRG.26217 | antisense | chr11:671371-672681:- | 12.10 | 29.70 | 1.30 | 0.0086 | 0.0441 |
| MSTRG.74764 | linc | chr17:82298162-82300389:+ | 70.20 | 151.85 | 1.11 | 0.0086 | 0.0442 |
| MSTRG.10640 | linc | chr1:156708351-156711081:- | 69.54 | 149.43 | 1.10 | 0.0086 | 0.0442 |
| ENSG00000264078 | antisense | chr1:31644694-31649371:+ | 2.40 | 0.23 | -3.41 | 0.0086 | 0.0442 |
| MSTRG.128958 | antisense | chr4:40423554-40424008:+ | 0.30 | 11.08 | 5.21 | 0.0086 | 0.0442 |
| MSTRG.21467 | linc | chr10:69058405-69062276:- | 26.57 | 7.35 | -1.85 | 0.0086 | 0.0442 |
| ENSG00000259357 | antisense | chr1:150965245-150966256:+ | 3.02 | 10.08 | 1.74 | 0.0087 | 0.0442 |
| ENSG00000249835 | antisense | chr5:83531352-83581320:- | 0.46 | 3.86 | 3.08 | 0.0086 | 0.0442 |
| MSTRG.85735 | linc | chr2:6796480-6815235:- | 32.44 | 111.61 | 1.78 | 0.0087 | 0.0443 |
| ENSG00000260823 | lincRNA | chr16:56609501-56611375:- | 2.68 | 0.32 | -3.06 | 0.0087 | 0.0443 |
| MSTRG.157484 | linc | chr6:148617700-148624630:+ | 118.54 | 45.94 | -1.37 | 0.0087 | 0.0443 |
| ENSG00000236535 | sense_intronic | chr1:174009267-174016206:- | 27.86 | 65.92 | 1.24 | 0.0088 | 0.0443 |
| ENSG00000259915 | lincRNA | chr2:186354570-186356773:- | 37.10 | 17.56 | -1.08 | 0.0087 | 0.0443 |
| MSTRG.105327 | linc | chr20:49759799-49775398:- | 11.42 | 64.90 | 2.51 | 0.0088 | 0.0446 |
| MSTRG.126706 | linc | chr4:6893724-6899452:+ | 2.47 | 11.94 | 2.27 | 0.0089 | 0.0447 |
| MSTRG.148346 | linc | chr6:2798034-2799059:+ | 2.65 | 8.74 | 1.72 | 0.0089 | 0.0447 |
| MSTRG.66130 | antisense | chr16:69182009-69187283:- | 13.27 | 35.49 | 1.42 | 0.0089 | 0.0447 |
| ENSG00000235016 | antisense | chr3:50116022-50156085:- | 0.14 | 2.14 | 3.92 | 0.0089 | 0.0448 |
| MSTRG.36165 | antisense | chr12:6952171-6953676:- | 84.21 | 228.25 | 1.44 | 0.0089 | 0.0448 |
| MSTRG.36995 | antisense | chr12:14498807-14500815:- | 13.64 | 32.13 | 1.24 | 0.0089 | 0.0448 |
| ENSG00000265625 | sense_intronic | chr17:29644796-29645847:- | 224.35 | 498.33 | 1.15 | 0.0089 | 0.0448 |
| MSTRG.52469 | antisense | chr14:50884046-50899815:- | 187.70 | 738.83 | 1.98 | 0.0090 | 0.0450 |
| MSTRG.166415 | antisense | chr7:135202734-135205026:+ | 15.94 | 40.96 | 1.36 | 0.0091 | 0.0453 |
| MSTRG.129233 | antisense | chr4:49313102-49511154:+ | 2.83 | 0.24 | -3.54 | 0.0091 | 0.0453 |
| MSTRG.166488 | antisense | chr7:102918736-102925820:- | 12.03 | 41.88 | 1.80 | 0.0091 | 0.0453 |
| ENSG00000273812 | lincRNA | chr20:62596732-62603115:- | 4.41 | 19.75 | 2.16 | 0.0091 | 0.0454 |
| ENSG00000224533 | antisense | chrX:155466540-155611616:+ | 7.67 | 21.35 | 1.48 | 0.0091 | 0.0454 |
| ENSG00000271780 | lincRNA | chr14:101948347-101949425:+ | 25.86 | 53.46 | 1.05 | 0.0091 | 0.0454 |
| MSTRG.30611 | antisense | chr11:66253161-66255658:+ | 42.41 | 86.71 | 1.03 | 0.0092 | 0.0457 |
| ENSG00000224086 | antisense | chr22:21938293-21977632:+ | 39.38 | 81.10 | 1.04 | 0.0092 | 0.0457 |
| MSTRG.22422 | antisense | chr10:79371316-79391580:+ | 51.27 | 164.36 | 1.68 | 0.0093 | 0.0457 |
| ENSG00000229368 | sense_overlapping | chr11:3854612-3855399:+ | 13.02 | 30.41 | 1.22 | 0.0093 | 0.0457 |
| MSTRG.17607 | linc | chr1:248802812-248804807:- | 35.64 | 73.91 | 1.05 | 0.0093 | 0.0458 |
| MSTRG.109789 | antisense | chr22:23146395-23153690:- | 20.22 | 61.53 | 1.61 | 0.0093 | 0.0458 |
| ENSG00000240012 | antisense | chr3:143342246-143347071:+ | 11.29 | 3.07 | -1.88 | 0.0093 | 0.0458 |
| MSTRG.186627 | linc | chrX:1668824-1669790:+ | 0.99 | 6.15 | 2.64 | 0.0093 | 0.0458 |
| MSTRG.145119 | antisense | chr5:134402475-134403640:+ | 66.64 | 157.92 | 1.24 | 0.0093 | 0.0458 |
| ENSG00000237576 | antisense | chr2:68832044-68837724:+ | 21.38 | 63.76 | 1.58 | 0.0094 | 0.0462 |
| ENSG00000250906 | antisense | chr4:40812779-40826151:+ | 0.47 | 3.43 | 2.88 | 0.0095 | 0.0464 |
| MSTRG.79330 | linc | chr18:77162446-77174076:+ | 51.96 | 23.03 | -1.17 | 0.0095 | 0.0464 |
| MSTRG.30403 | antisense | chr11:64742972-64752130:+ | 16.52 | 36.01 | 1.12 | 0.0095 | 0.0464 |
| ENSG00000273585 | lincRNA | chr15:101613676-101614339:- | 0.77 | 4.25 | 2.46 | 0.0096 | 0.0466 |
| MSTRG.84313 | antisense | chr19:48914125-48917270:+ | 23.45 | 51.55 | 1.14 | 0.0096 | 0.0467 |
| MSTRG.116043 | linc | chr3:46109460-46111163:- | 96.04 | 435.19 | 2.18 | 0.0096 | 0.0468 |
| MSTRG.194932 | linc | chrX:153411449-153417866:+ | 3.02 | 12.92 | 2.10 | 0.0096 | 0.0468 |
| ENSG00000260166 | lincRNA | chr16:88087387-88087932:+ | 9.63 | 23.38 | 1.28 | 0.0097 | 0.0469 |
| ENSG00000267226 | antisense | chr18:58670009-58671877:- | 11.16 | 3.81 | -1.55 | 0.0096 | 0.0469 |
| ENSG00000232415 | antisense | chr7:74059576-74062284:- | 2.02 | 0.17 | -3.59 | 0.0097 | 0.0469 |
| ENSG00000258731 | antisense | chr14:53153354-53157528:+ | 59.48 | 29.64 | -1.00 | 0.0097 | 0.0470 |
| ENSG00000204625 | lincRNA | chr6:29975112-29978410:+ | 2.17 | 9.72 | 2.16 | 0.0097 | 0.0470 |
| ENSG00000262712 | sense_intronic | chr16:4335870-4337818:- | 5.05 | 13.61 | 1.43 | 0.0098 | 0.0472 |
| MSTRG.72756 | linc | chr17:59408838-59433609:+ | 27.81 | 58.56 | 1.07 | 0.0098 | 0.0473 |
| ENSG00000276248 | sense_intronic | chr13:113527260-113530621:+ | 39.71 | 80.65 | 1.02 | 0.0099 | 0.0477 |
| MSTRG.84564 | antisense | chr19:51153857-51159442:+ | 23.97 | 69.69 | 1.54 | 0.0099 | 0.0477 |
| ENSG00000276900 | antisense | chr12:31729117-31731204:+ | 214.05 | 510.74 | 1.25 | 0.0100 | 0.0477 |
| MSTRG.126298 | antisense | chr4:2739959-2776934:+ | 15.07 | 34.97 | 1.21 | 0.0100 | 0.0478 |
| MSTRG.89748 | linc | chr2:57302110-57310849:- | 7.19 | 2.11 | -1.77 | 0.0100 | 0.0478 |
| MSTRG.73566 | linc | chr17:73281285-73282773:- | 5.81 | 21.72 | 1.90 | 0.0100 | 0.0478 |
| ENSG00000232220 | antisense | chr19:53874626-53876049:- | 0.14 | 2.20 | 3.96 | 0.0100 | 0.0478 |
| ENSG00000273355 | lincRNA | chr18:813274-813756:+ | 14.52 | 5.60 | -1.38 | 0.0100 | 0.0479 |
| ENSG00000271133 | antisense | chr7:20328299-20331747:- | 5.27 | 1.23 | -2.10 | 0.0101 | 0.0481 |
| MSTRG.11711 | linc | chr1:167206370-167216652:- | 6.69 | 1.78 | -1.91 | 0.0101 | 0.0481 |
| ENSG00000271840 | lincRNA | chr1:22100613-22101360:+ | 46.21 | 99.10 | 1.10 | 0.0101 | 0.0481 |
| ENSG00000260743 | lincRNA | chr3:179340322-179341887:+ | 13.73 | 5.37 | -1.35 | 0.0101 | 0.0481 |
| MSTRG.116838 | linc | chr3:53279116-53280396:+ | 0.18 | 2.49 | 3.75 | 0.0102 | 0.0482 |
| ENSG00000257715 | antisense | chr12:96025323-96027971:+ | 4.25 | 12.74 | 1.58 | 0.0102 | 0.0483 |
| MSTRG.121342 | linc | chr3:129641029-129647614:- | 83.15 | 244.69 | 1.56 | 0.0102 | 0.0483 |
| ENSG00000230454 | lincRNA | chr3:50260303-50263358:+ | 55.91 | 115.78 | 1.05 | 0.0102 | 0.0484 |
| ENSG00000254295 | lincRNA | chr5:172954907-172957162:+ | 13.86 | 31.74 | 1.20 | 0.0102 | 0.0484 |
| ENSG00000261745 | sense_overlapping | chr6:53125644-53126146:- | 0.46 | 3.30 | 2.84 | 0.0103 | 0.0484 |
| MSTRG.173920 | linc | chr8:89886567-89887245:- | 35.69 | 83.32 | 1.22 | 0.0103 | 0.0485 |
| MSTRG.104503 | antisense | chr20:36639130-36671471:+ | 14.12 | 32.55 | 1.20 | 0.0103 | 0.0485 |
| ENSG00000269867 | sense_intronic | chr19:57867038-57868172:- | 2.95 | 9.43 | 1.67 | 0.0104 | 0.0487 |
| MSTRG.71750 | antisense | chr17:45431635-45432932:+ | 0.33 | 2.90 | 3.12 | 0.0104 | 0.0487 |
| MSTRG.18596 | linc | chr10:6412953-6415456:- | 50.13 | 24.24 | -1.05 | 0.0104 | 0.0487 |
| ENSG00000214708 | antisense | chr17:32141226-32143135:- | 1.75 | 6.25 | 1.84 | 0.0105 | 0.0491 |
| MSTRG.82046 | linc | chr19:26777418-26822476:+ | 2.69 | 0.32 | -3.09 | 0.0105 | 0.0491 |
| MSTRG.9139 | linc | chr1:116091095-116149212:+ | 32.05 | 88.00 | 1.46 | 0.0106 | 0.0492 |
| ENSG00000280145 | lincRNA | chr21:6630182-6670695:- | 22.56 | 7.34 | -1.62 | 0.0106 | 0.0493 |
| ENSG00000260470 | lincRNA | chr12:54145069-54147225:- | 3.39 | 10.43 | 1.62 | 0.0106 | 0.0493 |
| MSTRG.105346 | linc | chr20:50207065-50213890:+ | 22.21 | 114.74 | 2.37 | 0.0106 | 0.0493 |
| ENSG00000259483 | lincRNA | chr14:56303490-56310761:- | 89.45 | 36.47 | -1.29 | 0.0106 | 0.0493 |
| MSTRG.2206 | linc | chr1:27315128-27319009:+ | 14.64 | 34.07 | 1.22 | 0.0106 | 0.0493 |
| MSTRG.28819 | antisense | chr11:35246663-35258058:+ | 140.58 | 59.17 | -1.25 | 0.0106 | 0.0494 |
| MSTRG.743 | linc | chr1:8923126-8939681:+ | 1.59 | 5.90 | 1.89 | 0.0107 | 0.0495 |
| MSTRG.2067 | antisense | chr1:26171013-26174592:+ | 17.91 | 7.37 | -1.28 | 0.0107 | 0.0495 |
| MSTRG.54226 | linc | chr14:72964207-72966907:- | 21.74 | 47.25 | 1.12 | 0.0107 | 0.0495 |
| MSTRG.139359 | antisense | chr5:56175999-56281130:+ | 24.60 | 8.22 | -1.58 | 0.0108 | 0.0495 |
| MSTRG.13210 | linc | chr1:185341048-185374885:+ | 24.53 | 50.57 | 1.04 | 0.0108 | 0.0496 |
| ENSG00000254694 | antisense | chr11:126208611-126209027:- | 19.37 | 8.34 | -1.21 | 0.0108 | 0.0497 |
| MSTRG.112882 | antisense | chr3:5245611-5261006:+ | 2.16 | 7.87 | 1.86 | 0.0108 | 0.0498 |
| ENSG00000248510 | lincRNA | chr4:96310701-96818864:+ | 10.69 | 2.92 | -1.87 | 0.0109 | 0.0498 |
| MSTRG.105243 | linc | chr20:49108359-49111363:+ | 19.81 | 50.93 | 1.36 | 0.0109 | 0.0498 |
| ENSG00000233968 | antisense | chr10:19710328-19728550:- | 125.91 | 42.56 | -1.56 | 0.0109 | 0.0499 |
| MSTRG.44421 | antisense | chr12:121839219-121849862:+ | 8.01 | 19.18 | 1.26 | 0.0109 | 0.0499 |
| ENSG00000246016 | antisense | chr5:10479371-10482309:+ | 4.84 | 23.81 | 2.30 | 0.0109 | 0.0499 |

Table S4 The lncRNAs with predicted genes in the 14 validation positive lncRNAs and their predicted genes. “#” means the gene is correlated with the lncRNA; “*” means the gene is within 50kb of the lncRNA.

| **LncRNA** | **Gene** | **log2 Fold Change** | **Up/**  **Down** | **Gene Name** | **Position** |
| --- | --- | --- | --- | --- | --- |
| ENSG00000234389 | ENSG00000115604* | 0.31 | up | IL18R1 | chr2:102311529-102398775:+ |
| ENSG00000234389 | ENSG00000180251* | - | up | SLC9A4 | chr2:102473303-102533972:+ |
| ENSG00000246263 | ENSG00000003402# | 1.11 | up | CFLAR | chr2:201116104-201176687:+ |
| ENSG00000246263 | ENSG00000008086# | 0.73 | up | CDKL5 | chrX:18425583-18653629:+ |
| ENSG00000246263 | ENSG00000035664# | 1.54 | up | DAPK2 | chr15:63907036-64072033:- |
| ENSG00000246263 | ENSG00000048392* | 0.55 | up | RRM2B | chr8:102204502-102239118:- |
| ENSG00000246263 | ENSG00000059728# | 1.54 | up | MXD1 | chr2:69897688-69942945:+ |
| ENSG00000246263 | ENSG00000063046# | -0.37 | down | EIF4B | chr12:53006158-53042209:+ |
| ENSG00000246263 | ENSG00000073331# | 1.51 | up | ALPK1 | chr4:112285509-112442620:+ |
| ENSG00000246263 | ENSG00000073910# | 0.95 | up | FRY | chr13:32031300-32299122:+ |
| ENSG00000246263 | ENSG00000076003# | -0.83 | down | MCM6 | chr2:135839626-135876426:- |
| ENSG00000246263 | ENSG00000089505# | 1.69 | up | CMTM1 | chr16:66566393-66579137:+ |
| ENSG00000246263 | ENSG00000107099# | 0.69 | up | DOCK8 | chr9:214854-465259:+ |
| ENSG00000246263 | ENSG00000111837# | 1.25 | up | MAK | chr6:10762723-10838555:- |
| ENSG00000246263 | ENSG00000113013# | -0.57 | down | HSPA9 | chr5:138554882-138575444:- |
| ENSG00000246263 | ENSG00000120051# | 1.66 | up | CFAP58 | chr10:104353764-104455090:+ |
| ENSG00000246263 | ENSG00000120318# | 1.53 | up | ARAP3 | chr5:141653401-141682221:- |
| ENSG00000246263 | ENSG00000126261# | -0.79 | down | UBA2 | chr19:34428352-34471251:+ |
| ENSG00000246263 | ENSG00000128595# | -0.53 | down | CALU | chr7:128739292-128771807:+ |
| ENSG00000246263 | ENSG00000132155# | 0.92 | up | RAF1 | chr3:12583601-12664226:- |
| ENSG00000246263 | ENSG00000132670# | -0.32 | down | PTPRA | chr20:2864184-3039076:+ |
| ENSG00000246263 | ENSG00000133398# | -0.47 | down | MED10 | chr5:6371881-6378594:- |
| ENSG00000246263 | ENSG00000134077# | -0.40 | down | THUMPD3 | chr3:9362842-9386791:+ |
| ENSG00000246263 | ENSG00000137177# | 1.19 | up | KIF13A | chr6:17759183-17987623:- |
| ENSG00000246263 | ENSG00000139436# | 0.53 | up | GIT2 | chr12:109929792-109996389:- |
| ENSG00000246263 | ENSG00000142168# | -0.58 | down | SOD1 | chr21:31659622-31668931:+ |
| ENSG00000246263 | ENSG00000143669# | 0.60 | up | LYST | chr1:235661041-235883640:- |
| ENSG00000246263 | ENSG00000143727# | -0.69 | down | ACP1 | chr2:264140-278283:+ |
| ENSG00000246263 | ENSG00000148229# | -0.24 | down | POLE3 | chr9:113407235-113410672:- |
| ENSG00000246263 | ENSG00000153989# | -0.54 | down | NUS1 | chr6:117675502-117710640:+ |
| ENSG00000246263 | ENSG00000160593# | 1.46 | up | JAML | chr11:118193740-118225094:- |
| ENSG00000246263 | ENSG00000161040# | 1.41 | up | FBXL13 | chr7:102813230-103074843:- |
| ENSG00000246263 | ENSG00000166900# | 1.62 | up | STX3 | chr11:59755059-59805882:+ |
| ENSG00000246263 | ENSG00000174130# | 1.27 | up | TLR6 | chr4:38823715-38856817:- |
| ENSG00000246263 | ENSG00000179195# | -0.81 | down | ZNF664 | chr12:123971845-124015439:+ |
| ENSG00000246263 | ENSG00000181220# | 1.15 | up | ZNF746 | chr7:149472794-149497817:- |
| ENSG00000246263 | ENSG00000182022# | 1.19 | up | CHST15 | chr10:124007666-124093607:- |
| ENSG00000246263 | ENSG00000198223# | 1.30 | up | CSF2RA | chrX:1268800-1310381:+ |
| ENSG00000246263 | ENSG00000198668# | -0.39 | down | CALM1 | chr14:90396502-90408261:+ |
| ENSG00000246263 | ENSG00000256537# | -0.47 | down | SMIM10L1 | chr12:11171222-11176016:+ |
| ENSG00000246263 | ENSG00000273841# | -0.42 | down | TAF9 | chr5:69364743-69370013:- |
| ENSG00000276649 | ENSG00000003402# | 1.11 | up | CFLAR | chr2:201116104-201176687:+ |
| ENSG00000276649 | ENSG00000006125# | -0.15 | down | AP2B1 | chr17:35578046-35726409:+ |
| ENSG00000276649 | ENSG00000008086# | 0.73 | up | CDKL5 | chrX:18425583-18653629:+ |
| ENSG00000276649 | ENSG00000035664# | 1.54 | up | DAPK2 | chr15:63907036-64072033:- |
| ENSG00000276649 | ENSG00000064547# | 1.51 | up | LPAR2 | chr19:19623668-19628930:- |
| ENSG00000276649 | ENSG00000073910# | 0.95 | up | FRY | chr13:32031300-32299122:+ |
| ENSG00000276649 | ENSG00000077157# | 1.03 | up | PPP1R12B | chr1:202348699-202592706:+ |
| ENSG00000276649 | ENSG00000078668# | -0.47 | down | VDAC3 | chr8:42391624-42405897:+ |
| ENSG00000276649 | ENSG00000096384# | -0.51 | down | HSP90AB1 | chr6:44246166-44253888:+ |
| ENSG00000276649 | ENSG00000100296# | 0.90 | up | THOC5 | chr22:29505879-29555216:- |
| ENSG00000276649 | ENSG00000102753# | -0.63 | down | KPNA3 | chr13:49699307-49792921:- |
| ENSG00000276649 | ENSG00000105339# | 1.38 | up | DENND3 | chr8:141117278-141195808:+ |
| ENSG00000276649 | ENSG00000107099# | 0.69 | up | DOCK8 | chr9:214854-465259:+ |
| ENSG00000276649 | ENSG00000109118# | 0.72 | up | PHF12 | chr17:28905250-28951771:- |
| ENSG00000276649 | ENSG00000113328# | -1.04 | down | CCNG1 | chr5:163437569-163446151:+ |
| ENSG00000276649 | ENSG00000114656# | 1.52 | up | KIAA1257 | chr3:128909866-129002690:- |
| ENSG00000276649 | ENSG00000119335# | -0.49 | down | SET | chr9:128683424-128696400:+ |
| ENSG00000276649 | ENSG00000120318# | 1.53 | up | ARAP3 | chr5:141653401-141682221:- |
| ENSG00000276649 | ENSG00000136371# | 1.62 | up | MTHFS | chr15:79833585-79897379:- |
| ENSG00000276649 | ENSG00000139436# | 0.53 | up | GIT2 | chr12:109929792-109996389:- |
| ENSG00000276649 | ENSG00000142168# | -0.58 | down | SOD1 | chr21:31659622-31668931:+ |
| ENSG00000276649 | ENSG00000142552# | 1.63 | up | RCN3 | chr19:49527618-49546962:+ |
| ENSG00000276649 | ENSG00000144746# | -0.82 | down | ARL6IP5 | chr3:69084944-69106066:+ |
| ENSG00000276649 | ENSG00000148200# | 1.67 | up | NR6A1 | chr9:124517275-124771310:- |
| ENSG00000276649 | ENSG00000148229# | -0.24 | down | POLE3 | chr9:113407235-113410672:- |
| ENSG00000276649 | ENSG00000160131# | -0.62 | down | VMA21 | chrX:151396515-151409364:+ |
| ENSG00000276649 | ENSG00000166579# | 0.85 | up | NDEL1 | chr17:8413131-8490411:+ |
| ENSG00000276649 | ENSG00000166900# | 1.62 | up | STX3 | chr11:59755059-59805882:+ |
| ENSG00000276649 | ENSG00000166987# | 1.43 | up | MBD6 | chr12:57520710-57530148:+ |
| ENSG00000276649 | ENSG00000167874# | 1.54 | up | TMEM88 | chr17:7855065-7856099:+ |
| ENSG00000276649 | ENSG00000168010# | 1.30 | up | ATG16L2 | chr11:72814308-72843674:+ |
| ENSG00000276649 | ENSG00000174238# | 0.99 | up | PITPNA | chr17:1517718-1562816:- |
| ENSG00000276649 | ENSG00000177169# | 1.18 | up | ULK1 | chr12:131894651-131923167:+ |
| ENSG00000276649 | ENSG00000180817# | -0.77 | down | PPA1 | chr10:70202830-70233911:- |
| ENSG00000276649 | ENSG00000181220# | 1.15 | up | ZNF746 | chr7:149472794-149497817:- |
| ENSG00000276649 | ENSG00000183323# | 1.13 | up | CCDC125 | chr5:69280175-69332809:- |
| ENSG00000276649 | ENSG00000198668# | -0.39 | down | CALM1 | chr14:90396502-90408261:+ |
| ENSG00000276649 | ENSG00000213281# | -0.47 | down | NRAS | chr1:114704469-114716894:- |
| ENSG00000276649 | ENSG00000250254# | 1.24 | up | PTTG2 | chr4:37960435-37961125:+ |
| ENSG00000280832 | ENSG00000008516# | 2.14 | up | MMP25 | chr16:3046681-3060726:+ |
| ENSG00000280832 | ENSG00000012779# | 1.26 | up | ALOX5 | chr10:45374176-45446119:+ |
| ENSG00000280832 | ENSG00000033327# | 1.32 | up | GAB2 | chr11:78215297-78418348:- |
| ENSG00000280832 | ENSG00000070731# | 1.57 | up | ST6GALNAC2 | chr17:76565379-76586956:- |
| ENSG00000280832 | ENSG00000077420# | 0.91 | up | APBB1IP | chr10:26438203-26567803:+ |
| ENSG00000280832 | ENSG00000101336# | 1.43 | up | HCK | chr20:32052188-32101856:+ |
| ENSG00000280832 | ENSG00000110080#/* | 1.27 | up | ST3GAL4 | chr11:126355640-126440344:+ |
| ENSG00000280832 | ENSG00000110080#/* | 1.27 | up | ST3GAL4 | chr11:126355640-126440344:+ |
| ENSG00000280832 | ENSG00000112685# | -0.52 | down | EXOC2 | chr6:485133-693111:- |
| ENSG00000280832 | ENSG00000113749# | 1.51 | up | HRH2 | chr5:175658030-175710756:+ |
| ENSG00000280832 | ENSG00000113851# | -0.68 | down | CRBN | chr3:3148992-3179710:- |
| ENSG00000280832 | ENSG00000118900# | 1.20 | up | UBN1 | chr16:4846665-4882360:+ |
| ENSG00000280832 | ENSG00000130638# | -0.50 | down | ATXN10 | chr22:45671798-45845307:+ |
| ENSG00000280832 | ENSG00000132510# | 1.51 | up | KDM6B | chr17:7839904-7854796:+ |
| ENSG00000280832 | ENSG00000137145# | -0.69 | down | DENND4C | chr9:19230435-19373545:+ |
| ENSG00000280832 | ENSG00000140332# | 1.40 | up | TLE3 | chr15:70047790-70098176:- |
| ENSG00000280832 | ENSG00000142166# | 0.91 | up | IFNAR1 | chr21:33324477-33359862:+ |
| ENSG00000280832 | ENSG00000146094# | 1.63 | up | DOK3 | chr5:177501907-177511274:- |
| ENSG00000280832 | ENSG00000150455* | 0.49 | up | TIRAP | chr11:126283065-126298845:+ |
| ENSG00000280832 | ENSG00000152213# | 1.39 | up | ARL11 | chr13:49628299-49633872:+ |
| ENSG00000280832 | ENSG00000160785# | 1.37 | up | SLC25A44 | chr1:156193932-156212796:+ |
| ENSG00000280832 | ENSG00000173281# | 1.74 | up | PPP1R3B | chr8:9136255-9151574:- |
| ENSG00000280832 | ENSG00000176788# | 1.97 | up | BASP1 | chr5:17065598-17276843:+ |
| ENSG00000280832 | ENSG00000180871# | 1.83 | up | CXCR2 | chr2:218125289-218137253:+ |
| ENSG00000280832 | ENSG00000181274# | 1.92 | up | FRAT2 | chr10:97332497-97334709:- |
| ENSG00000280832 | ENSG00000187676# | -0.85 | down | B3GLCT | chr13:31199936-31332276:+ |
| ENSG00000280832 | ENSG00000196663# | 1.66 | up | TECPR2 | chr14:102362963-102502481:+ |
| ENSG00000280832 | ENSG00000243646# | 1.13 | up | IL10RB | chr21:33266358-33310187:+ |
| MSTRG.149099 | ENSG00000112799* | -0.33 | down | LY86 | chr6:6588108-6654983:+ |
| MSTRG.166799 | ENSG00000059378* | 0.25 | up | PARP12 | chr7:140023744-140063721:- |
| MSTRG.172533 | ENSG00000003402# | 1.11 | up | CFLAR | chr2:201116104-201176687:+ |
| MSTRG.172533 | ENSG00000063046# | -0.37 | down | EIF4B | chr12:53006158-53042209:+ |
| MSTRG.172533 | ENSG00000063177# | -0.37 | down | RPL18 | chr19:48615328-48619536:- |
| MSTRG.172533 | ENSG00000065427# | -0.31 | down | KARS | chr16:75627474-75648643:- |
| MSTRG.172533 | ENSG00000065615# | 0.52 | up | CYB5R4 | chr6:83859643-83967424:+ |
| MSTRG.172533 | ENSG00000073331# | 1.51 | up | ALPK1 | chr4:112285509-112442620:+ |
| MSTRG.172533 | ENSG00000073910# | 0.95 | up | FRY | chr13:32031300-32299122:+ |
| MSTRG.172533 | ENSG00000077157# | 1.03 | up | PPP1R12B | chr1:202348699-202592706:+ |
| MSTRG.172533 | ENSG00000078668# | -0.47 | down | VDAC3 | chr8:42391624-42405897:+ |
| MSTRG.172533 | ENSG00000083223# | 0.84 | up | ZCCHC6 | chr9:86287733-86354454:- |
| MSTRG.172533 | ENSG00000083845# | -0.63 | down | RPS5 | chr19:58386400-58394806:+ |
| MSTRG.172533 | ENSG00000087157# | 0.93 | up | PGS1 | chr17:78378640-78425114:+ |
| MSTRG.172533 | ENSG00000089009# | -0.68 | down | RPL6 | chr12:112405190-112418838:- |
| MSTRG.172533 | ENSG00000089157# | -0.48 | down | RPLP0 | chr12:120196686-120201235:- |
| MSTRG.172533 | ENSG00000089289# | -0.43 | down | IGBP1 | chrX:70133449-70166324:+ |
| MSTRG.172533 | ENSG00000092201# | -0.16 | down | SUPT16H | chr14:21351472-21384266:- |
| MSTRG.172533 | ENSG00000096063# | 0.88 | up | SRPK1 | chr6:35832966-35921342:- |
| MSTRG.172533 | ENSG00000096384# | -0.51 | down | HSP90AB1 | chr6:44246166-44253888:+ |
| MSTRG.172533 | ENSG00000100138# | -0.29 | down | SNU13 | chr22:41673930-41690504:- |
| MSTRG.172533 | ENSG00000105339# | 1.38 | up | DENND3 | chr8:141117278-141195808:+ |
| MSTRG.172533 | ENSG00000109118# | 0.72 | up | PHF12 | chr17:28905250-28951771:- |
| MSTRG.172533 | ENSG00000109466# | 0.86 | up | KLHL2 | chr4:165207618-165323156:+ |
| MSTRG.172533 | ENSG00000112062# | 0.89 | up | MAPK14 | chr6:36027677-36111236:+ |
| MSTRG.172533 | ENSG00000115484# | -0.48 | down | CCT4 | chr2:61868089-61888804:- |
| MSTRG.172533 | ENSG00000118263# | 0.90 | up | KLF7 | chr2:207074137-207167267:- |
| MSTRG.172533 | ENSG00000120318# | 1.53 | up | ARAP3 | chr5:141653401-141682221:- |
| MSTRG.172533 | ENSG00000122203# | -0.34 | down | KIAA1191 | chr5:176346061-176361968:- |
| MSTRG.172533 | ENSG00000124767# | -0.67 | down | GLO1 | chr6:38675925-38703141:- |
| MSTRG.172533 | ENSG00000132341# | -0.43 | down | RAN | chr12:130871879-130877678:+ |
| MSTRG.172533 | ENSG00000132383# | -0.35 | down | RPA1 | chr17:1829702-1900082:+ |
| MSTRG.172533 | ENSG00000135365# | 0.76 | up | PHF21A | chr11:45929323-46121178:- |
| MSTRG.172533 | ENSG00000136247# | -0.43 | down | ZDHHC4 | chr7:6577434-6589374:+ |
| MSTRG.172533 | ENSG00000138185# | 0.87 | up | ENTPD1 | chr10:95711779-95869695:+ |
| MSTRG.172533 | ENSG00000139436# | 0.53 | up | GIT2 | chr12:109929792-109996389:- |
| MSTRG.172533 | ENSG00000142168# | -0.58 | down | SOD1 | chr21:31659622-31668931:+ |
| MSTRG.172533 | ENSG00000143797# | 1.11 | up | MBOAT2 | chr2:8852690-9003813:- |
| MSTRG.172533 | ENSG00000145293# | -0.58 | down | ENOPH1 | chr4:82430562-82461091:+ |
| MSTRG.172533 | ENSG00000147454# | 1.69 | up | SLC25A37 | chr8:23528805-23575463:+ |
| MSTRG.172533 | ENSG00000148229# | -0.24 | down | POLE3 | chr9:113407235-113410672:- |
| MSTRG.172533 | ENSG00000148303# | -0.35 | down | RPL7A | chr9:133348214-133351426:+ |
| MSTRG.172533 | ENSG00000154589* | 1.22 | up | LY96 | chr8:73991352-74029087:+ |
| MSTRG.172533 | ENSG00000155744# | 0.70 | up | FAM126B | chr2:200973718-201071671:- |
| MSTRG.172533 | ENSG00000166579# | 0.85 | up | NDEL1 | chr17:8413131-8490411:+ |
| MSTRG.172533 | ENSG00000166900# | 1.62 | up | STX3 | chr11:59755059-59805882:+ |
| MSTRG.172533 | ENSG00000167658# | -0.25 | down | EEF2 | chr19:3976056-3985469:- |
| MSTRG.172533 | ENSG00000168010# | 1.30 | up | ATG16L2 | chr11:72814308-72843674:+ |
| MSTRG.172533 | ENSG00000169567# | -0.57 | down | HINT1 | chr5:131159027-131171735:- |
| MSTRG.172533 | ENSG00000171490# | -0.37 | down | RSL1D1 | chr16:11833850-11851585:- |
| MSTRG.172533 | ENSG00000171867# | -0.56 | down | PRNP | chr20:4686236-4701590:+ |
| MSTRG.172533 | ENSG00000174444# | -0.63 | down | RPL4 | chr15:66498015-66524532:- |
| MSTRG.172533 | ENSG00000174748# | -0.55 | down | RPL15 | chr3:23916545-23923692:+ |
| MSTRG.172533 | ENSG00000175606* | 0.24 | up | TMEM70 | chr8:73972437-73982783:+ |
| MSTRG.172533 | ENSG00000184983# | -0.38 | down | NDUFA6 | chr22:42085525-42090955:- |
| MSTRG.172533 | ENSG00000188906# | 1.05 | up | LRRK2 | chr12:40196744-40369285:+ |
| MSTRG.172533 | ENSG00000196262# | -0.41 | down | PPIA | chr7:44796680-44824564:+ |
| MSTRG.172533 | ENSG00000197818# | 0.80 | up | SLC9A8 | chr20:49812713-49892242:+ |
| MSTRG.172533 | ENSG00000204628# | -0.33 | down | RACK1 | chr5:181236909-181248096:- |
| MSTRG.63013 | ENSG00000004700# | -0.66 | down | RECQL | chr12:21468911-21501669:- |
| MSTRG.63013 | ENSG00000006327* | 2.68 | up | TNFRSF12A | chr16:3018445-3022383:+ |
| MSTRG.63013 | ENSG00000008517* | -0.08 | down | IL32 | chr16:3065297-3082192:+ |
| MSTRG.63013 | ENSG00000065150# | -0.76 | down | IPO5 | chr13:97953658-98024297:+ |
| MSTRG.63013 | ENSG00000067248# | -0.62 | down | DHX29 | chr5:55256245-55307722:- |
| MSTRG.63013 | ENSG00000102753# | -0.63 | down | KPNA3 | chr13:49699307-49792921:- |
| MSTRG.63013 | ENSG00000103145* | 0.69 | up | HCFC1R1 | chr16:3022620-3024286:- |
| MSTRG.63013 | ENSG00000106591# | -0.72 | down | MRPL32 | chr7:42932200-42948958:+ |
| MSTRG.63013 | ENSG00000115233# | -0.88 | down | PSMD14 | chr2:161308038-161411717:+ |
| MSTRG.63013 | ENSG00000122068# | -0.64 | down | FYTTD1 | chr3:197737179-197787596:+ |
| MSTRG.63013 | ENSG00000122958# | -0.33 | down | VPS26A | chr10:69123512-69172861:+ |
| MSTRG.63013 | ENSG00000130182* | 0.76 | up | ZSCAN10 | chr16:3088890-3099317:- |
| MSTRG.63013 | ENSG00000131652* | -0.04 | down | THOC6 | chr16:3024027-3027755:+ |
| MSTRG.63013 | ENSG00000136758# | -0.24 | down | YME1L1 | chr10:27110112-27155266:- |
| MSTRG.63013 | ENSG00000137040# | -0.83 | down | RANBP6 | chr9:6011043-6015625:- |
| MSTRG.63013 | ENSG00000137492# | -0.50 | down | THAP12 | chr11:76349956-76380971:- |
| MSTRG.63013 | ENSG00000138757# | -0.50 | down | G3BP2 | chr4:75642782-75724525:- |
| MSTRG.63013 | ENSG00000162069* | 3.56 | up | BICDL2 | chr16:3027682-3036926:- |
| MSTRG.63013 | ENSG00000163320# | -0.57 | down | CGGBP1 | chr3:88051944-88149885:- |
| MSTRG.63013 | ENSG00000168385# | -0.39 | down | SEPT2 | chr2:241315100-241354027:+ |
| MSTRG.63013 | ENSG00000168724# | -0.52 | down | DNAJC21 | chr5:34929593-34958964:+ |
| MSTRG.63013 | ENSG00000172115# | -1.12 | down | CYCS | chr7:25120091-25125361:- |
| MSTRG.63013 | ENSG00000184697* | - | up | CLDN6 | chr16:3014712-3020071:- |
| MSTRG.63013 | ENSG00000213937* | 2.27 | up | CLDN9 | chr16:3012456-3014505:+ |
| MSTRG.64433 | ENSG00000003402# | 1.11 | up | CFLAR | chr2:201116104-201176687:+ |
| MSTRG.64433 | ENSG00000012171# | 1.73 | up | SEMA3B | chr3:50267558-50277546:+ |
| MSTRG.64433 | ENSG00000064547# | 1.51 | up | LPAR2 | chr19:19623668-19628930:- |
| MSTRG.64433 | ENSG00000077157# | 1.03 | up | PPP1R12B | chr1:202348699-202592706:+ |
| MSTRG.64433 | ENSG00000078668# | -0.47 | down | VDAC3 | chr8:42391624-42405897:+ |
| MSTRG.64433 | ENSG00000103035# | -0.40 | down | PSMD7 | chr16:74296775-74306288:+ |
| MSTRG.64433 | ENSG00000105339# | 1.38 | up | DENND3 | chr8:141117278-141195808:+ |
| MSTRG.64433 | ENSG00000107099# | 0.69 | up | DOCK8 | chr9:214854-465259:+ |
| MSTRG.64433 | ENSG00000125977# | -0.47 | down | EIF2S2 | chr20:34088298-34112332:- |
| MSTRG.64433 | ENSG00000133943# | 1.08 | up | C14orf159 | chr14:91060333-91225632:+ |
| MSTRG.64433 | ENSG00000136758# | -0.24 | down | YME1L1 | chr10:27110112-27155266:- |
| MSTRG.64433 | ENSG00000139436# | 0.53 | up | GIT2 | chr12:109929792-109996389:- |
| MSTRG.64433 | ENSG00000141337# | 0.90 | up | ARSG | chr17:68259182-68422731:+ |
| MSTRG.64433 | ENSG00000142192# | -0.87 | down | APP | chr21:25880550-26171128:- |
| MSTRG.64433 | ENSG00000142552# | 1.63 | up | RCN3 | chr19:49527618-49546962:+ |
| MSTRG.64433 | ENSG00000147400# | -0.70 | down | CETN2 | chrX:152826973-152830777:- |
| MSTRG.64433 | ENSG00000168010# | 1.30 | up | ATG16L2 | chr11:72814308-72843674:+ |
| MSTRG.64433 | ENSG00000169180* | 1.32 | up | XPO6 | chr16:28097979-28211920:- |
| MSTRG.64433 | ENSG00000169181* | 2.57 | up | GSG1L | chr16:27787535-28063509:- |
| MSTRG.64433 | ENSG00000181220# | 1.15 | up | ZNF746 | chr7:149472794-149497817:- |
| MSTRG.64433 | ENSG00000184900# | -0.26 | down | SUMO3 | chr21:44805617-44818779:- |
| MSTRG.64433 | ENSG00000204305# | 0.99 | up | AGER | chr6:32180968-32184324:- |
| MSTRG.64433 | ENSG00000250254# | 1.24 | up | PTTG2 | chr4:37960435-37961125:+ |

Table S5 The differentially expressed lncRNAs screened from T1D and T2D.

| **Gene** | **Biotype** | **Position** | **T1D**  **_normalize** | **T2D**  **_normalize** | **log2**  **Fold**  **Change** | **p-val** | **FDR** |
| --- | --- | --- | --- | --- | --- | --- | --- |
| ENSG00000224515 | antisense | chr1:161556290-161557078:- | 11.99 | 0.65 | 4.21 | 3.83E-06 | 8.81E-03 |
| MSTRG.22725 | linc | chr10:87486843-87489249:+ | 9.04 | 0.79 | 3.52 | 1.01E-05 | 1.16E-02 |
| ENSG00000250437 | lincRNA | chr5:89581209-89677701:+ | 21.97 | 2.46 | 3.16 | 1.64E-05 | 1.26E-02 |
| ENSG00000257764 | antisense | chr12:69353493-69354225:- | 1.04 | 19.88 | -4.25 | 8.34E-05 | 2.13E-02 |
| MSTRG.166799 | antisense | chr7:139951772-139984965:- | 3.32 | 22.27 | -2.75 | 3.72E-05 | 2.14E-02 |
| MSTRG.130492 | linc | chr4:77819789-77820296:- | 31.89 | 171.72 | -2.43 | 8.22E-05 | 2.36E-02 |
| MSTRG.128697 | linc | chr4:39817649-39818260:- | 5.48 | 0.36 | 3.91 | 7.95E-05 | 2.61E-02 |
| MSTRG.111149 | antisense | chr22:32808734-32811415:- | 6.54 | 0.73 | 3.17 | 1.23E-04 | 2.83E-02 |
| MSTRG.63104 | linc | chr16:4540405-4541587:+ | 4.18 | 0.08 | 5.69 | 6.30E-05 | 2.90E-02 |
| MSTRG.23167 | linc | chr10:90051364-90100406:+ | 28.13 | 6.35 | 2.15 | 7.94E-05 | 3.04E-02 |

Table S6 The differentially expressed mRNAs screened from type 1 diabetes and type 2 diabetes.

| **Gene** | **Gene name** | **Position** | **T1D**  **_normalize** | **T2D**  **_normalize** | **log2**  **Fold**  **Change** | **p-val** | **FDR** |
| --- | --- | --- | --- | --- | --- | --- | --- |
| ENSG00000279576 | AP000769.1 | chr11:65502034-65503622:- | 7.48 | 296.95 | -5.31 | 5.15E-20 | 6.55E-17 |
| ENSG00000115155 | OTOF | chr2:26457203-26558698:- | 18.32 | 263.78 | -3.85 | 3.35E-16 | 2.13E-13 |
| ENSG00000147206 | NXF3 | chrX:103075810-103093125:- | 2.4 | 72.9 | -4.92 | 4.89E-15 | 2.07E-12 |
| ENSG00000165949 | IFI27 | chr14:94104836-94116698:+ | 4.99 | 63.84 | -3.68 | 2.04E-10 | 6.49E-08 |
| ENSG00000183844 | FAM3B | chr21:41304212-41357431:+ | 42.72 | 1.78 | 4.59 | 1.77E-07 | 4.51E-05 |
| ENSG00000146950 | SHROOM2 | chrX:9786456-9949443:+ | 2.97 | 80.69 | -4.77 | 1.43E-06 | 3.04E-04 |
| ENSG00000213931 | HBE1 | chr11:5268345-5505617:- | 29.67 | 1.33 | 4.48 | 1.69E-05 | 2.68E-03 |
| ENSG00000110203 | FOLR3 | chr11:72114869-72139892:+ | 182.27 | 534.28 | -1.55 | 1.52E-05 | 2.76E-03 |
| ENSG00000167034 | NKX3-1 | chr8:23678693-23682927:- | 121.94 | 25.56 | 2.25 | 2.39E-05 | 3.38E-03 |
| ENSG00000166831 | RBPMS2 | chr15:64739892-64775587:- | 155.02 | 34.58 | 2.16 | 2.95E-05 | 3.75E-03 |
| ENSG00000143297 | FCRL5 | chr1:157513377-157552520:- | 754.2 | 274.17 | 1.46 | 4.64E-05 | 5.35E-03 |
| ENSG00000168917 | SLC35G2 | chr3:136818647-136855892:+ | 4.25 | 25.09 | -2.56 | 5.40E-05 | 5.72E-03 |
| ENSG00000172058 | SERF1A | chr5:70900665-70918530:+ | 13.26 | 1.59 | 3.06 | 6.76E-05 | 6.60E-03 |
| ENSG00000147647 | DPYS | chr8:104330324-104467053:- | 0.15 | 5.95 | -5.35 | 2.10E-04 | 1.90E-02 |
| ENSG00000130748 | TMEM160 | chr19:47045907-47048630:- | 13.39 | 51.46 | -1.94 | 2.65E-04 | 2.24E-02 |

Table S7 The lncRNAs validated in expanded cohorts of type 1 diabetes (n=11) and type 2 diabetes (n=56). “*” means the gene is significant difference in RNA-seq.

| **LncRNA** | **Novel/known** | **The log2 value in the RNA-seq data** | **Significant difference in validation cohort** | | **Orthologous sequence with mouse** |
| --- | --- | --- | --- | --- | --- |
| ENSG00000224515 | known | -4.21* | | no | Not found |
| ENSG00000269902 | known | 1.09 | | yes | Not found |
| MSTRG.128697 | novel | -3.91* | | yes | Not found |
| MSTRG.128958 | novel | - | | no | chr5:66,016,825-66,017,169 |
| MSTRG.166799 | novel | 2.75* | | no | chr6:39,016,934-39,045,231 |
| MSTRG.23167 | novel | -2.15* | | no | chr19:35,211,979-35,262,091 |
| MSTRG.63013 | novel | 0.11 | | yes | chr17:23,644,824-23,660,240 |
| MSTRG.72098 | novel | -1.68 | | no | chr11:95,397,976-95,398,838 |
| MSTRG.74858 | novel | 1.48 | | yes | chr11:121,806,938-121,808,036 |

Table S8 The positive validation lncRNAs with predicted mRNA and their targeted genes.

| **LncRNA** | **Gene Name** | **Up/Down** |
| --- | --- | --- |
| ENSG00000234389 | IL18R1 | up |
|  | SLC9A4 | up |
|  | CFLAR | up |
|  | CDKL5 | up |
|  | DAPK2 | up |
|  | RRM2B | up |
|  | MXD1 | up |
|  | EIF4B | down |
|  | ALPK1 | up |
|  | FRY | up |
|  | MCM6 | down |
|  | CMTM1 | up |
|  | DOCK8 | up |
|  | MAK | up |
|  | HSPA9 | down |
|  | CFAP58 | up |
|  | ARAP3 | up |
| ENSG00000246263 | UBA2 | down |
|  | CALU | down |
|  | RAF1 | up |
|  | PTPRA | down |
|  | MED10 | down |
|  | THUMPD3 | down |
|  | KIF13A | up |
|  | GIT2 | up |
|  | SOD1 | down |
|  | LYST | up |
|  | ACP1 | down |
|  | POLE3 | down |
|  | NUS1 | down |
|  | JAML | up |
|  | FBXL13 | up |
|  | STX3 | up |
|  | TLR6 | up |
|  | ZNF664 | down |
|  | ZNF746 | up |
|  | CHST15 | up |
|  | CSF2RA | up |
|  | CALM1 | down |
|  | SMIM10L1 | down |
|  | TAF9 | down |
|  | CFLAR | up |
|  | AP2B1 | down |
|  | CDKL5 | up |
|  | DAPK2 | up |
|  | LPAR2 | up |
|  | FRY | up |
|  | PPP1R12B | up |
|  | VDAC3 | down |
|  | HSP90AB1 | down |
|  | THOC5 | up |
|  | KPNA3 | down |
|  | DENND3 | up |
|  | DOCK8 | up |
|  | PHF12 | up |
|  | CCNG1 | down |
|  | KIAA1257 | up |
|  | SET | down |
| ENSG00000276649 | ARAP3 | up |
|  | MTHFS | up |
|  | GIT2 | up |
|  | SOD1 | down |
|  | RCN3 | up |
|  | ARL6IP5 | down |
|  | NR6A1 | up |
|  | POLE3 | down |
|  | VMA21 | down |
|  | NDEL1 | up |
|  | STX3 | up |
|  | MBD6 | up |
|  | TMEM88 | up |
|  | ATG16L2 | up |
|  | PITPNA | up |
|  | ULK1 | up |
|  | PPA1 | down |
|  | ZNF746 | up |
|  | CCDC125 | up |
|  | CALM1 | down |
|  | NRAS | down |
|  | PTTG2 | up |
| MSTRG.166799 | PARP12 | up |
|  | CFLAR | up |
|  | EIF4B | down |
|  | RPL18 | down |
|  | KARS | down |
|  | CYB5R4 | up |
|  | ALPK1 | up |
|  | FRY | up |
|  | PPP1R12B | up |
|  | VDAC3 | down |
|  | ZCCHC6 | up |
|  | RPS5 | down |
|  | PGS1 | up |
|  | RPL6 | down |
|  | RPLP0 | down |
|  | IGBP1 | down |
|  | SUPT16H | down |
|  | SRPK1 | up |
|  | HSP90AB1 | down |
|  | SNU13 | down |
|  | DENND3 | up |
|  | PHF12 | up |
|  | KLHL2 | up |
|  | MAPK14 | up |
|  | CCT4 | down |
|  | KLF7 | up |
|  | ARAP3 | up |
|  | KIAA1191 | down |
| MSTRG.172533 | GLO1 | down |
|  | RAN | down |
|  | RPA1 | down |
|  | PHF21A | up |
|  | ZDHHC4 | down |
|  | ENTPD1 | up |
|  | GIT2 | up |
|  | SOD1 | down |
|  | MBOAT2 | up |
|  | ENOPH1 | down |
|  | SLC25A37 | up |
|  | POLE3 | down |
|  | RPL7A | down |
|  | LY96 | up |
|  | FAM126B | up |
|  | NDEL1 | up |
|  | STX3 | up |
|  | EEF2 | down |
|  | ATG16L2 | up |
|  | HINT1 | down |
|  | RSL1D1 | down |
|  | PRNP | down |
|  | RPL4 | down |
|  | RPL15 | down |
|  | TMEM70 | up |
|  | NDUFA6 | down |
|  | LRRK2 | up |
|  | PPIA | down |
|  | SLC9A8 | up |
|  | RACK1 | down |
|  | RECQL | down |
|  | TNFRSF12A | up |
|  | IL32 | down |
|  | IPO5 | down |
|  | DHX29 | down |
|  | KPNA3 | down |
|  | HCFC1R1 | up |
|  | MRPL32 | down |
|  | PSMD14 | down |
|  | FYTTD1 | down |
| MSTRG.63013 | VPS26A | down |
|  | ZSCAN10 | up |
|  | THOC6 | down |
|  | YME1L1 | down |
|  | RANBP6 | down |
|  | THAP12 | down |
|  | G3BP2 | down |
|  | BICDL2 | up |
|  | CGGBP1 | down |
|  | 2-Sep | down |
|  | DNAJC21 | down |
|  | CYCS | down |
|  | CLDN6 | up |
|  | CLDN9 | up |
|  | CFLAR | up |
|  | SEMA3B | up |
|  | LPAR2 | up |
|  | PPP1R12B | up |
|  | VDAC3 | down |
|  | PSMD7 | down |
|  | DENND3 | up |
|  | DOCK8 | up |
|  | EIF2S2 | down |
|  | C14orf159 | up |
| MSTRG.64433 | YME1L1 | down |
|  | GIT2 | up |
|  | ARSG | up |
|  | APP | down |
|  | RCN3 | up |
|  | CETN2 | down |
|  | ATG16L2 | up |
|  | XPO6 | up |
|  | GSG1L | up |
|  | ZNF746 | up |
|  | SUMO3 | down |
|  | AGER | up |
|  | PTTG2 | up |
